# Supplementary material for: Knowledge translation strategies for policy and action focused on sexual, reproductive, maternal, newborn, child and adolescent health and well-being: a rapid scoping review
Source: BMJ Open. 2022 Jan 17;12(1):e053919. doi: 10.1136/bmjopen-2021-053919 (PMC8765012; doi:10.1136/bmjopen-2021-053919)
Supplement: Supplementary data [file bmjopen-2021-053919supp001.pdf]

Supplementary File 1- Search Strategy

1 exp Diffusion of Innovation/
2 Information Dissemination/
3 Translational Medical Research/
4 ((disseminat* or implement* or share? or sharing or translat*) adj2 (activities or activity or approach? or model? or plan? or planning or program* or strategies or strategy or advanc* or better* or boost* or enhanc* or forward* or further* or improv* or increas* or bolster* or sustain* or uphold*)).ti,ab,kw,kf.
5 (evidence* adj2 (adapt* or adopt* or diffus* or disseminat* or exchang* or implement* or mobili* or share? or sharing or transfer* or translat* or utilis* or utiliz* or uptake)).ti,ab,kw,kf.
6 (information adj2 (adapt* or adopt* or diffus* or disseminat* or exchang* or mobili* or propagat* or share? or sharing or spread* or transfer* or translat*)).ti,ab,kw,kf.
7 (innovation? adj2 (adapt* or adopt* or diffus* or disseminat* or exchang* or implement* or mobili* or propagat* or share? or sharing or spread* or transfer* or translat* or transmi* or uptake)).ti,ab,kw,kf.
8 (knowledge adj2 (adapt* or adopt* or diffus* or disseminat* or exchang* or implement* or mobili* or propagat* or share? or sharing or spread* or transfer* or translat* or transmi* or uptake)).ti,ab,kw,kf.
9 (research adj2 (adapt* or adopt* or diffus* or disseminat* or exchang* or implement* or mobili* or propagat* or share? or sharing or spread* or transfer* or translat* or transmi* or utilis* or utiliz* or uptake)).ti,ab,kw,kf.
10 or/1-9 [KT]
11 Cartoons as Topic/
12 exp Mass Media/
13 Pamphlets/
14 Newspapers as Topic/
15 Webcasts as Topic/
16 Guidelines as Topic/
17 ((knowledge or evidence) adj (product? or summary or summaries or brief* or report? or portal? or repositor* or database? or center? or centre?)).ti,ab,kw,kf.
18 (advocacy brief* or strategy brief*).ti,ab,kw,kf.
19 (position statement? or consensus statement? or policy statement?).ti,ab,kw,kf.
20 (booklet? or brochure? or cartoon* or comic? or flyer? or infograph* or pamphlet? or poster?).ti,ab,kw,kf.
21 (media adj2 (communicat* or diffus* or disseminat* or exchang* or mobili* or populari* or propagat* or publici* or share? or sharing or spread*)).ti,ab,kw,kf.
22 (briefing? or news or newspaper? or press release?).ti,ab,kw,kf.
23 (thematic report? or accountability report? or evaluation report?).ti,ab,kw,kf.
24 (strategic plan? or annual report? or progress report?).ti,ab,kw,kf.
25 (online communication? or web communication? or video? or webcast* or podcast*).ti,ab,kw,kf.
26 (toolkit? or tool or tools or roadmap? or road map?).ti,ab,kw,kf.
27 (guideline? or standards).ti,ab,kw,kf.

28 or/11-27 [products]
29 10 and 28 [KT products]
30 ((evidence or information or innovation? or knowledge or research) adj2 (adapt* or adopt* or diffus* or disseminat* or exchang* or implement* or mobili* or propagat* or share? or sharing or spread* or transfer* or translat* or transmi* or uptake) adj2 (activities or activity or approach? or initiative? or plan? or policies or policy or procedure? or program* or scheme? or strategies or strategy)).ti,ab,kw,kf.
31 or/1-3
32 (activities or activity or approach? or initiative? or plan? or policies or policy or procedure? or program* or scheme? or strategies or strategy).ti.
33 31 and 32 [KT activities]
34 30 or 33 [KT activities or strategies]
35 29 or 34 [KT products, activities, or strategies (INTERVENTION)]
36 exp Administrative Personnel/
37 exp Policy Making/
38 (administrator? or decision maker? or policy maker? or policymaker?).ti,ab.
39 (knowledge user? or stakeholder?).ti,ab.
40 (advisory or board or committee? or task force?).ti,ab.
41 (((policy or policies) adj making) or policymaking or ((policy or policies) adj2 develop*) or ((policy or policies) adj2 analys?s) or (health adj (policy or policies))).ti,ab.
42 (legislator? or legislation?).ti,ab.
43 (health system? or health care system? or healthcare system? or system? level or system? manage*).ti,ab.
44 (civil societ* or third sector or non government* organi?ation? or nongovernment* organi?ation? or ngo or ngos).ti,ab.
45 (donor? or funder? or funding agen* or grant* agen*).ti,ab.
46 or/36-45 [POPULATION]
47 Reproductive Health/ or Reproductive Health Services/ or Family Planning Services/ or Sexual Health/ or Circumcision, Female/ or Menstruation/ or exp Puberty/ or exp Contraception/ or exp Sexually Transmitted Diseases/ or exp Sex Offenses/ or Intimate Partner Violence/ or Gender-Based Violence/ or Sex Work/ or exp Human papillomavirus 16/ or Human papillomavirus 18/ or Uterine Cervical Neoplasms/ or (reproductive or reproduction or family planning or sexual* or early marriage or child marriage or child bride? or female genital mutilation or female circumcision or menstruation or menstrual or puberty or contraception or contraceptive* or condom? or sexually transmitted infection* or sexually transmitted disease* or hiv or aids or coerc* or rape or forced sex or abortion? or intimate partner violence or gender based violence or sex work* or transactional sex or hpv or cervical cancer?).ti,ab,kw,kf.
48 exp Women's Health/ or exp Women's Health Services/ or Women/ or (wom#n? or female? or mother? or maternal or widow?).ti,ab,kw,kf.
49 exp Maternal Health Services/ or Pregnant Women/ or exp Pregnancy/ or (birth or childbirth or mother* or maternal* or maternity or preconception or prenatal* or perinatal* or antepartum or ante-partum or intrapartum or intra-partum or neonatal* or neo-natal* or postnatal* or post-natal* or postpartum or post-partum or pregnan*).ti,ab,kw,kf.

50 exp Infant/ or (fetus* or foetus* or fetal* or foetal* or baby or babies or neonate* or neo-nate* or newborn* or new-born* or infant*).ti,ab,kw,kf.

51 exp Child/ or exp Pediatrics/ or (child* or kid or kids or girl or girls or boy or boys or preschool* or pre-school* or kindergarten* or school age* or elementary school* or juvenile* or minors or p?ediatric? or first-grader* or second-grader* or third-grader* or fourth-grader* or fifth-grader* or sixth-grader* or seventh-grader* or eighth-grader* or middle school* or junior high*).ti,ab,kw,kf.

52 Adolescent/ or Young Adult/ or (teen* or youth* or adolescen* or juvenile* or (young adj2 (adult* or person* or individual* or people* or population*)) or youngster* or highschool* or ((secondary or high*) adj2 (school* or education)) or pubescen*).ti,ab,kw,kf.

53 or/47-52 [RMNCAH filter]

54 35 and 46 and 53
