# Supplementary material for: Knowledge translation strategies for policy and action focused on sexual, reproductive, maternal, newborn, child and adolescent health and well-being: a rapid scoping review
Source: BMJ Open. 2022 Jan 17;12(1):e053919. doi: 10.1136/bmjopen-2021-053919 (PMC8765012; doi:10.1136/bmjopen-2021-053919)
Supplement: Supplementary data [file bmjopen-2021-053919supp002.pdf]

Supplementary File 2- Description of Included Studies

Author & Year	Country	Country Income Level	Primary Objective	Health Topic	SRMNCAH Priority	Study Design	Sample Characteristics	BCW Intervention Functions Summary	BCW Policy Categories Summary	Direction of Effect
Abney-Roberts et al., 2015[1]	USA	High	To prevent sleep-related infant deaths by applying recommendations from the American Academy of Pediatrics	Newborn sleep	Newborn health/wellbeing or stillbirths	Quasi-experimental	150 healthcare providers (e.g., nurses and physicians from obstetrics, pediatrics and family medicine)	Education		Positive
Agapidaki et al., 2013[2]	Greece	High	To improve pediatricians' ability to identify and manage maternal depression by implementing a targeted education program	Maternal depression	Maternal health/wellbeing	Experimental	43 pediatricians	Education<br><br>Environmental Restructuring<br><br>Persuasion	Guidelines	Positive
Agency for Healthcare Research and Quality, 2016[3]	USA	High	To decrease maternal and neonatal adverse events, and to improve patient safety, team communication, and quality of care	Perinatal care	Maternal health/wellbeing<br><br>Newborn health/wellbeing or stillbirths	Observational	Labour & delivery units	Education<br><br>Environmental Restructuring<br><br>Training	Service Provision	Positive
Ainsworth et al., 2014[4]	USA	High	To reduce and prevent newborn falls	Newborn fall prevention	Newborn health/wellbeing or stillbirths	Quasi-experimental	Healthcare providers, mothers and their infants	Education<br><br>Environmental Restructuring	Regulation	Positive
Akter et al., 2009[5]	Bangladesh	Middle	To identify changes in antimicrobial use following the implementation of an educational intervention targeting physicians	Antimicrobial use	Child health/wellbeing	Quasi-experimental	All physicians working in pediatric wards at the participating hospital	Education		Positive
Al-Rafay & Al-Sharkawy, 2012[6]	Egypt	Middle	To evaluate changes in nurses' knowledge following an education program to address total parenteral nutrition guidelines	Newborn nutrition	Newborn health/wellbeing or stillbirths	Quasi-experimental	40 nurses	Education<br><br>Training	Guidelines	Positive
Alkon et al., 2014[7]	USA	High	To evaluate a nutrition and physical activity program implemented at childcare centres	Child nutrition and physical activity	Child health/wellbeing	Experimental	137 childcare providers from 17 childcare centres. Centres included a total of 552 children who were in the program (aged 3-5 years)	Education<br><br>Environmental Restructuring	Guidelines	Positive

Allen & Jeffery, 2006[8]	Nepal	Middle	To design and implement an educational newborn care program targeting healthcare providers to decrease infant morbidity and mortality	Newborn care	Newborn health/wellbeing or stillbirths	Quasi-experimental	30 healthcare providers (e.g., junior doctors, nurses, community health workers)	Education		Positive
Allen & Schafer, 2015[9]	USA	High	To implement practices which promote optimal infant feeding	Infant nutrition	Newborn health/wellbeing or stillbirths	Quasi-experimental	Healthcare providers, pregnant women, and new mothers and their infants	Education<br><br>Environmental Restructuring<br><br>Enablement	Service provision<br><br>Communication/ Marketing<br><br>Environmental/ Social Planning	Positive
Alton et al. 2006[10]	USA	High	To describe a pediatric patient safety program	Pediatric patient safety	Child health/wellbeing	Observational	Healthcare providers, health systems managers	Education<br><br>Environmental Restructuring	Service provision<br><br>Communication/ Marketing<br><br>Environmental/ Social Planning<br><br>Regulation	Positive
Alvarez et al., 2019[11]	Peru and Uganda	Low and middle	To determine the useability and usefulness of a workbook to provide support and guidance in global health systems	Maternal and newborn health	Maternal Health/Wellbeing<br><br>Newborn health/wellbeing or stillbirths	Qualitative	8 participants (e.g., researchers, policymakers or other stakeholders)	Education	Guidelines	Positive
Anaby et al., 2015[12]	Canada	High	To improve healthcare providers' knowledge and to change practice to support children with disabilities	Activities with children and youth with physical disabilities	Child health/wellbeing	Qualitative	14 healthcare providers (e.g., occupational therapists, physiotherapists, speech language pathologist)	Education	Service Provision	Positive
Ansbro et al., 2015[13]	Zambia	Middle	To explore healthcare providers' experience of using rapid syphilis testing	Syphilis	Sexual or reproductive health/rights	Qualitative	40 healthcare providers		Guidelines	Positive
Baer et al., 2011[14]	USA	High	To determine the effectiveness of a NICU blood transfusion and monitoring system 12 months following implementation	Neonatal blood transfusions	Newborn health/wellbeing or stillbirths	Quasi-experimental	Four NICUs	Environmental Restructuring<br><br>Enablement<br><br>Coercion<br><br>Restriction	Service Provision	Positive
Barnard et al., 2017[15]	USA	High	To identify barriers and enablers to establishing standing orders to vaccinate during pregnancy	Vaccination during pregnancy	Maternal health/wellbeing	Qualitative	38 healthcare providers and staff members (e.g., medical directors,	Education	Service Provision	Positive

							practice administrators, nurses, medical assistants)			
Becker-Haimes et al., 2017[16]	USA	High	To explore the acceptability and feasibility of a toolkit designed for healthcare providers working across mental health settings.	Youth mental health	Adolescent health/wellbeing	Mixed methods	Quantitative: 70 clinicians<br><br>Qualitative: Six randomly selected clinicians from the quantitative study	Education		Positive
Berglund et al., 2010[17]	Ukraine	Middle	To determine the effectiveness of the W.H.O's Effective Perinatal Care package implemented at maternity hospitals	Perinatal care	Maternal Health/Wellbeing	Quasi-experimental	Healthcare providers at three maternity hospitals (e.g., obstetricians, neonatologists, pediatricians, anesthesiologists, midwives, pediatric nurses)	Environmental Restructuring<br><br>Training<br><br>Enablement		Positive
Boyko et al., 2016[18]	Canada	High	To explore the usefulness of deliberative dialogue utilized by the Preventing Violence Across the Lifespan (PreVAiL) Research Network during a biennial team meeting	Child maltreatment	Child Health/Wellbeing	Mixed methods	44 participants in the deliberative dialogue (e.g., researchers, knowledge users, policymakers)	Education		Positive
Braddick et al., 2016[19]	Uganda	Low	To assess compliance with postpartum hemorrhage clinical guideline recommendations, and identify barriers and enablers to using the recommendations	Postpartum hemorrhage	Maternal Health/Wellbeing	Mixed methods	Quantitative: 154 births<br>Qualitative: 18 healthcare provider interviews (e.g., physicians, midwives)	Education	Guidelines	Neutral
Brennan et al., 2013[20]	Ghana	Middle	To increase nurses' knowledge and skills in pediatric resuscitation through the WHO Emergency Triage and Assessment Treatment program	Pediatric resuscitation	Child Health/Wellbeing	Quasi-experimental	41 nurses	Education<br><br>Training<br><br>Modelling		Positive
Brown et al., 2005[21]	UK	High	To identify the barriers creating delays in surgery and develop guidelines for management of undescended tests	Undescended testes	Newborn Health/Wellbeing or Stillbirths	Observational	Healthcare providers and hospital staff	Education<br><br>Environmental Restructuring	Service Provision<br><br>Guidelines	Positive

Burgoine et al., 2018[22]	Uganda	Low	To reduce neonatal mortality through the implementation of two neonatal health interventions	Neonatal mortality	Newborn Health/Wellbeing or Stillbirths	Quasi-experimental	57 healthcare providers (e.g., midwives, nurses, interns)	Education<br><br>Environmental Restructuring<br><br>Training<br><br>Enablement	Guidelines<br><br>Environmental/ Social Planning	Positive
Cameron et al., 2011[23]	Canada	High	To determine healthcare administrators' perceptions of the barriers and enablers in using a knowledge broker to support implementing measures aimed at children with cerebral palsy needs	Cerebral palsy	Child Health/Wellbeing	Qualitative	Healthcare administrators from 27 pediatric healthcare centres	Education<br><br>Environmental Restructuring	Service Provision	Positive
Carlo et al., 2009[24]	Zambia	Middle	To improve nurse midwives' knowledge and skills following completion of an American Academy of Pediatrics Neonatal Resuscitation Program	Neonatal resuscitation	Newborn Health/Wellbeing or Stillbirths	Observational	127 nurse midwives	Education<br><br>Training		Positive
Carmona et al., 2015[25]	USA	High	To improve healthcare providers' ability to provide high quality adolescent healthcare	Teen health	Adolescent Health/Wellbeing	Mixed methods	109 healthcare providers from 15 sites	Training	Guidelines	Positive
Close et al., 2016[26]	Madagascar	Low	To determine the effectiveness of a knowledge translation and implementation model aimed at training Peace Corps Volunteers to then train healthcare staff in rural communities	Neonatal resuscitation	Newborn Health/Wellbeing or Stillbirths	Quasi-experimental	10 Peace Corps Volunteers trained 42 healthcare providers in 10 communities	Training<br><br>Enablement		Positive
Crone et al., 2006[27]	Netherlands	High	To determine long-term use of a smoking cessation program to prevent passive smoking around children	Baby exposure to smoke in the household	Newborn Health/Wellbeing or Stillbirths	Observational	39 baby health clinic managers, 255 nurses and 68 physicians	Education<br><br>Training	Communication/ Marketing	Neutral
Cunningham & Cardy, 2020[28]	Canada	High	To improve speech language pathologists' knowledge and intention to utilize a tool designed to support participation-focused outcome measurements	Child speech	Child Health/Wellbeing	Quasi-experimental	46 speech language pathologists	Education<br><br>Persuasion		Positive
Daniels et al., 2008[29]	South Africa	Middle	To determine perceived and actual utilization of research in policy and legislation development	Eclampsia and pre-eclampsia	Maternal Health/Wellbeing	Qualitative	15 interviews with local researchers and policy makers	Education	Legislation	Positive

Davies et al., 2002[30]	Canada	High	To determine the effectiveness of an interactive, educational workshop to support nurses to implement fetal health surveillance guidelines	Labour and delivery	Maternal Health/Wellbeing	Mixed methods	Four hospitals sites (2 intervention, 2 control). Between 14-35 nurses from the intervention hospitals participated in each of the four education modules	Education<br><br>Environmental Restructuring<br><br>Training		Positive
Deorari et al., 2001[31]	India	Middle	To improve newborn health outcome and reduce incidence of newborn resuscitation by providing healthcare providers with neonatal resuscitation training	Neonatal resuscitation	Newborn Health/Wellbeing or Stillbirths	Quasi-experimental	Physicians and nurses at 14 hospitals	Environmental Restructuring<br><br>Training	Regulation	Positive
Di Noia et al., 2003[32]	USA	High	To promote a youth substance abuse prevention program through pamphlets, CD-ROMs, and the Internet to encourage use and recommendations for the program	Adolescent substance abuse prevention	Adolescent Health/Wellbeing	Experimental	188 staff members employed at junior high schools, youth-focused non-profit organizations, and government bodies aimed at preventing youth substance abuse	Education<br><br>Environmental Restructuring		Positive
Ding et al., 2008[33]	China	Middle	To reduce inappropriate antibiotic prescribing in the pediatric intensive care unit (PICU)	Antibiotic prescribing	Child Health/Wellbeing	Quasi-experimental	Pediatricians	Education<br><br>Environmental Restructuring	Guidelines<br><br>Environmental/ Social Planning	Positive
Dobbins et al., 2009[34]	Canada	High	To evaluate three KT strategies aimed at supporting healthy childhood bodyweight	Childhood obesity	Child Health/Wellbeing	Experimental	108 public health departments	Education<br><br>Environmental Restructuring<br><br>Training		Neutral
Edwards et al., 2016[35]	Jamaica, Kenya, Uganda and South Africa	Low and Middle	To improve HIV care by implementing nurse leadership hubs	HIV	Sexual or reproductive health/rights	Quasi-experimental	167 participants (e.g., nurses, staff nurses, managers, researchers, decision makers, community representatives)	Education<br><br>Environmental Restructuring<br><br>Training<br><br>Enablement	Communication/ Marketing	Neutral
English et al., 2010[36]	USA	High	To outline the design and implementation of a perinatal tobacco cessation program	Perinatal tobacco cessation	Maternal Health/Wellbeing	Mixed methods	11 lay case managers attended the first training session, 8 participated in the second session	Education<br><br>Training<br><br>Modelling		Neutral

Eriksson et al., 2017[37]	Vietnam	Middle	To identify barriers and enablers to implementation and sustainability of a knowledge translation strategy, the Neonatal Knowledge Into Practice (NeoKIP) trial	Neonatal mortality	Maternal Health/Wellbeing<br><br>Newborn Health/Wellbeing or Stillbirths	Qualitative	Interviews: 6 interviews with healthcare directors or representatives from healthcare facilities<br><br>Focus groups: 6 focus groups with previous stakeholders in the NeoKIP trial	Environmental Restructuring<br><br>Training		Positive
Farner et al., 2014[38]	USA	High	To improve healthcare providers' screening for critical congenital heart disease through the design, implement and evaluate an educational program	Newborn screening for critical congenital heart disease	Newborn Health/Wellbeing or Stillbirths	Quasi-experimental	215 nurses participated across 13 hospitals	Training<br><br>Modelling<br><br>Environmental restructuring<br><br>Education	Regulation	Positive
Finch et al., 2012[39]	Australia	High	To increase use of an intervention to encourage physical activity for children at childcare centres, and to improve childcare managers' knowledge of physical activity recommendations for children and perceived acceptability of the intervention	Childhood physical activity	Child Health/Wellbeing	Quasi-experimental	228 childcare centres participated in the intervention, 164 childcare centres were the control	Training<br><br>Enablement<br><br>Incentivisation		Positive
Finch et al., 2019[40]	Australia	High	To determine effectiveness of an intervention aimed at improving compliance with childcare nutrition guidelines in childcare centres	Childhood nutrition	Child Health/Wellbeing	Experimental	24 childcare centres participated in the intervention, 20 participated in the control from baseline data collection	Education<br><br>Training<br><br>Environmental Restructuring	Regulation	Neutral
George et al., 2018[41]	Sri Lanka	Middle	To hold a consultation with regional users of the WHO safe childbirth checklist to promote knowledge exchange, support improve maternal health outcomes, and offer high quality care during childbirth	Labour and delivery	Maternal Health/Wellbeing	Qualitative	60 participants (e.g., obstetricians, gynecologists, nurses, researchers, implementers, government officials, and 12 funders from low, middle, and high-income countries)	Education		Positive
Gera et al., 2019[42]	India	Middle	To evaluate an intervention aimed at improving newborn vaccination uptake in health facilities	Newborn vaccination	Newborn Health/Wellbeing or Stillbirths	Mixed Methods	141 public health facilities participated in the intervention<br><br>Qualitative: interviews with health managers	Training		Positive

Gichane et al., 2019[43]	Zambia	Middle	To outline the process of advocating for policy changes to allow for non-clinical healthcare workers to provide injectable contraception	Contraception/ Family planning	Sexual or reproductive health/rights	Editorial	Not reported	Education<br><br>Environmental Restructuring<br><br>Modelling	Legislation	Positive
Gilkey et al., 2019[44]	USA	High	To improve uptake of the HPV vaccine through use of a quality improvement program	HPV vaccination	Sexual or reproductive health/rights	Experimental	13 clinics received the intervention, while 12 were placed in the wait-list control group. 43 pediatric physicians participated across the intervention clinics, while 35 participated across the control clinics	Training<br><br>Incentivisation		Neutral
Goering & Wilson, 2002[45]	USA	High	To decrease variance and improve management of pre-term labour through the guideline implementation	Preterm labour	Maternal Health/Wellbeing	Observational	Nursing staff	Education<br><br>Enablement	Guidelines<br><br>Environmental/ Social planning	Positive
Goyet et al., 2014[46]	Cambodia	Middle	To identify barriers to KT strategies aimed at pneumonia	Pneumonia	Child Health/Wellbeing	Observational	Interventions targeted clinicians, health managers, policymakers	Education<br><br>Environmental Restructuring<br><br>Enablement		Neutral
Grady et al., 2019[47]	Australia	High	To determine effectiveness of a strategy aimed at improving compliance with dietary guidelines and daycare centres	Childhood nutrition	Child Health/Wellbeing	Quasi-experimental	27 daycare centres participated in the intervention services, 19 centres participated in the control services	Environmental Restructuring<br><br>Training<br><br>Enablement<br><br>Persuasion	Service Provision	Positive
Guillory, et al., 2017[48]	USA	High	To identify an appropriate implementation strategy for newborn screening for critical congenital heart disease across multiple hospitals	Newborn screening for critical congenital heart disease	Newborn Health/Wellbeing or Stillbirths	Quasi-experimental	117 physicians, hospital administrators and other healthcare professionals	Education<br><br>Environmental Restructuring<br><br>Training<br><br>Modelling		Positive

Hardee et al., 2019[49]	Nigeria and Uganda	Low and Middle	To determine effectiveness of two interventions which utilize a voluntary, rights-based family planning (VRBFP) approach to measure health and rights outcomes	Family planning	Sexual or reproductive health/rights	Mixed Methods	15 intervention healthcare facilities<br><br>Qualitative: 16 interviews with healthcare providers, health facility managers, supervisors, and representatives from collaborating human rights organizations<br><br>Focus Groups: male champions for the project, and facility and community healthcare workers	Education<br><br>Environmental Restructuring<br><br>Training<br><br>Enablement		Positive
Hulton et al., 2014[50]	Ethiopia, Ghana, Malawi, Nigeria, Sierra Leone, and Tanzania	Low and Middle	To outline and describe the Evidence for Action (E4A) program and its contributions to reduce rates of maternal and newborn mortality	Maternal and newborn mortality	Maternal Health/Wellbeing<br><br>Newborn health/wellbeing or stillbirths	Observational	Case studies of each of the E4A countries: Ethiopia, Ghana, Malawi, Nigeria, Sierra Leone, and Tanzania	Persuasion<br><br>Coercion	Communication/ Marketing<br><br>Guidelines<br><br>Regulation	Positive
Jeffery et al., 2004[51]	Macedonia	Middle	To outline the design and implementation of an education intervention aimed at improving perinatal health practices	Maternal and newborn mortality	Maternal Health/Wellbeing<br><br>Newborn health/wellbeing or stillbirths	Quasi-experimental	115 physicians and nurses	Education<br><br>Training		Positive
Jones et al., 2015[52]	Australia	High	To determine effectiveness of a complex intervention aimed at improving childcare nutrition and physical activity practices in childcare centres	Childhood nutrition and physical activity	Child Health/Wellbeing	Experimental	62 childcare centres participated in the intervention, while 60 centres served as the control	Education<br><br>Environmental Restructuring<br><br>Training<br><br>Enablement	Communication/ Marketing	Neutral
Kapungu et al., 2013[53]	Ghana	Middle	To reduce postpartum hemorrhage by designing and implementing a community-based misoprostol distribution intervention	Postpartum hemorrhage	Maternal Health/Wellbeing	Observational	74 healthcare providers	Education<br><br>Environmental Restructuring<br><br>Training<br><br>Enablement		Positive
Kingsnorth et al., 2020[54]	Canada	High	To address barriers and enablers to a knowledge translation intervention to improve clinic care	Children with physical disabilities	Child Health/Wellbeing	Observational	Healthcare providers	Education<br><br>Environmental Restructuring		Positive

			provided at a pediatric rehabilitation hospital	and complex health needs				Enablement<br><br>Persuasion		
Kraft et al., 2018 [55]	Ethiopia and Senegal	Low and Middle	To describe the perceived use and impact of using the WHO family planning guidance and tools	Family planning	Sexual or reproductive health/rights	Qualitative	Interviews with: 5 WHO regional advisers, 6 policy makers, 7 disseminators, 4 implementers and 4 end-users	Education	Guidelines	Positive
Lang et al., 2015[56]	USA	High	To improve healthcare providers' use of trauma-focused cognitive behavioural therapy and improve outcomes in children through the use of evidence-based practice	Mental health	Child Health/Wellbeing	Mixed methods	Across 16 participating agencies, 77 clinicians, 33 supervisors, and 23 senior leaders participated in training. 734 children across the agencies used the therapy approach	Education<br><br>Training<br><br>Persuasion		Positive
Makkar et al., 2016[57]	Australia	High	To test strategies to improve policymakers' use of Web Centre for Informing Policy in Health with Evidence from Research (CIPHER) usage and engagement with research evidence	Various sexual health topics (e.g., sexually transmissible infections, menopause, breast and cervical screening, and men's sexual health)	Sexual or reproductive health/rights	Observational	223 policymakers from 27 organisations accessed the platform during the study period	Education<br><br>Environmental Restructuring<br><br>Persuasion	Communication/ Marketing	Positive
Margolis et al., 2001[58]	USA	High	To improve the processes of health services and care delivery, particularly between healthcare providers and families, to support child health outcomes	Child health outcomes	Child Health/Wellbeing	Observational	8 pediatric and family healthcare practices participated	Education<br><br>Environmental Restructuring<br><br>Training<br><br>Enablement	Service Provision<br><br>Guidelines<br><br>Regulation	Positive
Mello et al., 2013[59]	USA	High	To identify adoption and implementation strategies for integrating an alcohol screening tool into regular practice for pediatric trauma patients	Alcohol use	Adolescent Health/Wellbeing	Mixed methods	7 pediatric trauma centres. A key informant was identified at each site<br><br>Qualitative: monthly check-in and feedback conversations with project managers at each site	Education<br><br>Training<br><br>Enablement	Service Provision	Positive

Melnyk et al., 2010[60]	USA	High	To implement the evidence-based Creating Opportunities for Personal Empowerment program and determine its effectiveness on nurses' beliefs of evidence-based practice	Premature birth	Newborn Health/Wellbeing or Stillbirths	Experimental	81 nurses (48 participated in implementation group and 33 participated in the control group)	Education<br><br>Training	Service Provision	Positive
Mody et al., 2013[61]	Bangladesh, China, Indonesia, Myanmar, Mongolia, Solomon Islands, Tonga, Vanuatu and Vietnam	Middle	To assess the use of family guidelines and associated tools as part of the Strategic Partnership Program	Family planning	Sexual or reproductive health/rights	Mixed methods	Workshop participants: program managers, Ministry of Health staff, clinicians and regional and country office staff from WHO/UNFPA.<br><br>Questionnaire: provided to Ministries of Health	Education<br><br>Training	Communication/ Marketing<br><br>Guidelines	Positive
Okonofua et al., 2011[62]	Nigeria	Middle	To describe the findings from multiple advocacy activities designed to provide free health services to pregnant women and children	Maternal and child mortality	Maternal Health/Wellbeing Child Health/Wellbeing	Observational	Interviews with 6 State Governors and/or Commissioners of Health	Education<br><br>Modelling<br><br>Persuasion	Communication/ Marketing<br><br>Guidelines	Positive
Ongolo-Zogo et al., 2018[63]	Cameroon and Uganda	Low and Middle	To assess how KT platforms have supported Millennium Development Goals through health system policy-making and decision making processes	Maternal and child health	Maternal Health/Wellbeing<br><br>Child Health/Wellbeing	Observational	Stakeholders and decision makers	Education<br><br>Environmental Restructuring<br><br>Training		Positive
Pariyo et al., 2005[64]	Uganda	Low	To determine the effects of scaling-up an intervention aimed at the integrated management of childhood illnesses to improve health outcomes in young children (under 5)	Childhood illness	Child Health/Wellbeing	Observational	316 healthcare facilities across 10 study districts. 427 healthcare workers participated across these facilities	Education<br><br>Training<br><br>Enablement		Neutral
Rowe et al., 2009[65]	Benin	Middle	To evaluate the effectiveness of additional training supports developed for healthcare providers who completed training in Integrated Management of Childhood Illness on child health outcomes	Childhood illness	Child Health/Wellbeing	Experimental	1244 consultations for any illness across 16 communes. Consultations were conducted by 267 healthcare workers at 114 health facilities.	Education<br><br>Environmental Restructuring<br><br>Training<br><br>Incentivisation		Neutral
Russell et al., 2010[66]	Canada	High	To evaluate knowledge brokers and their impact in a knowledge translation intervention and measurement tools to understand motor function in children with cerebral palsy	Cerebral palsy	Child Health/Wellbeing	Mixed methods	122 physiotherapists across three provinces	Education<br><br>Environmental Restructuring	Service Provision	Positive

Schreiber et al., 2009[67]	USA	High	The purpose of this study was to identify, implement, and evaluate strategies to support integrating scientific research evidence into clinical decision making	Physiotherapy	Child Health/Wellbeing	Observational	5 pediatric physiotherapists	Education<br><br>Environmental Restructuring<br><br>Training<br><br>Incentivisation		Positive
Schreiber et al., 2015[68]	USA	High	To outline a KT program designed to increase physiotherapists' knowledge and use of standardized outcome measures	Physiotherapy	Child Health/Wellbeing	Observational	17 physiotherapists from a pediatric outpatient facility	Education<br><br>Environmental Restructuring<br><br>Training<br><br>Modelling<br><br>Enablement<br><br>Persuasion		Positive
Schwartz et al., 2015[69]	USA	High	To use a multi-stakeholder partnership to develop and implement evidence-based breastfeeding support and training strategies	Breastfeeding	Maternal Health/Wellbeing	Quasi-experimental	8 health centres with predominantly culturally diverse patients implemented the 10 steps to support breastfeeding	Education<br><br>Environmental Restructuring<br><br>Training	Service Provision<br><br>Communication/ Marketing,<br><br>Guidelines<br><br>Environmental/ Social Planning	Positive
Segre et al., 2011[70]	USA	High	To describe the development and implementation of the train-the-trainer program aimed at maternal depression screening	Perinatal depression	Maternal Health/Wellbeing	Observational	42 individuals were trained from 32 maternal and child health agencies	Environmental Restructuring<br><br>Training		Positive
Segre et al., 2018[71]	USA	High	To describe a research and government partnership implementing 'listening visits' in health centres to support maternal mental health	Postpartum depression	Maternal Health/Wellbeing	Editorial	18 maternal health clinics	Education<br><br>Training<br><br>Enablement	Guidelines	Positive
Senarath et al., 2007[72]	Sri Lanka	Middle	To evaluate a program aimed at training healthcare providers to improve newborn care practices	Newborn care	Newborn Health/Wellbeing or Stillbirths	Experimental	120 healthcare providers. 59 participated in the intervention (27 midwives, 19 nurses and 13 physicians) while 61 participated in the control (26 midwives, 19 nurses and 16 physicians)	Education<br><br>Training		Positive

Seward et al., 2018[73]	Australia	High	To evaluate the effectiveness of a complex intervention aimed at improving compliance with child nutrition guidelines and children's dietary intake	Childhood nutrition	Child Health/Wellbeing	Experimental	45 childcare centres- 25 participated in the intervention and 20 participated in the control	Education<br><br>Environmental Restructuring<br><br>Persuasion	Guidelines	Positive
Simioni et al., 2017[74]	Argentina	Middle	To support the regionalization of a perinatal health strategy policy as a to improve health outcomes	Maternal and premature/ newborn health	Maternal Health/Wellbeing<br><br>Newborn Health/Wellbeing or Stillbirths	Mixed methods	Participants completed the first workshop and 49 participated in the second workshop	Training	Environmental/ Social Planning	Positive
Simmons, 2007[75] (Chapter 2)	Viet Nam	Middle	To describe the process of scaling up an intervention to provide injectable contraceptives to improve quality of care and family planning services	Contraception/ family planning	Sexual or reproductive health/rights	Observational	21 provinces in Viet Nam - Each provincial team included provincial health and family planning sector representatives	Education	Guidelines	Neutral
Simmons, 2007[76] (Chapter 8)	Brazil, Bolivia, Chile	Middle and High	To implement training the trainer methods to build capacity and provide innovative training in the area of reproductive health services	Reproductive health services	Sexual or reproductive health/rights	Mixed methods	Brazil: 9 training centre teams. 98 trainers were trained, and 1921 providers trained<br><br>Bolivia: 8 training centre teams. 34 trainers were trained, and 741 providers trained<br><br>Chile: 3 training centre teams. 21 trainers were trained, and 395 providers trained	Education<br><br>Environmental Restructuring<br><br>Training	Service Provision	Positive
Singh et al., 2013[77]	Ghana	Middle	To test local change ideas which could be scaled up later as part of a national child survival improvement project	Maternal and infant health	Maternal Health/Wellbeing<br><br>Newborn Health/Wellbeing or Stillbirths	Quasi-experimental	27 healthcare facilities	Education	Environmental/. Social Planning	Positive
Snelgrove-Clarke, 2010[78]	Canada	High	To evaluate two educational interventions to improve nurses' use of clinical guidelines during labour and delivery	Labour and delivery	Maternal Health/Wellbeing	Experimental	93 nurses participated in the first intervention and 62 nurses participated in the second intervention	Education<br><br>Enablement	Guidelines	Neutral

Sobel et al., 2011[79]	Philippines	Middle	To evaluate the impact of a training workshop for healthcare providers to provide newborns with a Hepatitis B vaccination, in line with a new vaccination policy	Newborn Hepatitis B vaccination	Newborn Health/Wellbeing or Stillbirths	Mixed methods	45 health centre staff, 120 community healthcare workers and 23 healthcare providers from 9 healthcare centers	Education<br><br>Environmental Restructuring	Guideline	Positive
Sundaram et al., 2015[80]	Ghana	Middle	To determine the effectiveness of a multi-stakeholder partnership program developed to provide comprehensive abortion and post-abortion care services	Abortion	Sexual or reproductive health/rights	Quasi-experimental	457 healthcare providers (116 physicians and 341 midwives, nurses, medical assistants) participated across 166 healthcare facilities.<br><br>The intervention group comprised 197 participants from 64 healthcare facilities. The first control group included 148 participants from 58 healthcare facilities, and the second control group included 112 participants across 44 healthcare facilities.	Environmental Restructuring<br><br>Training	Service Provision<br><br>Guidelines<br><br>Legislation	Positive
Tarasoff et al., 2014[81]	Canada	High	To evaluate the effectiveness of a theatre-based KT intervention design to improve healthcare providers' knowledge and attitudes of assisted human reproduction for LGBTQ people	Family planning	Sexual or reproductive health/rights	Qualitative	30 health professionals attended the workshop (e.g., physicians, nurses, social workers, health managers)	Training	Service Provision	Positive
Twum-Danso et al., 2014[82]	Ghana	Middle	To test the feasibility and scale up of a postnatal care policy to improve care	Postnatal care	Newborn Health/Wellbeing or Stillbirths	Quasi-experimental	30 teams from 27 rural health facilities	Education	Guidelines	Positive
Uneke et al., 2017[83]	Nigeria	Middle	To improve researchers and policymakers' the knowledge and capacity to use KT to promote evidence-informed policymaking in maternal, newborn and child health and wellbeing	Maternal, newborn and child health	Child Health/Wellbeing<br><br>Maternal Health/Wellbeing	Quasi-experimental	38 participants (e.g., researchers, government members) attended the workshop	Education		Positive

					Newborn Health/Wellbeing or Stillbirths					
Uneke et al., 2018[84]	Nigeria	Middle	To improve researchers and policymakers’ competence to apply KT to promote evidence-informed policymaking in maternal, newborn and child health and wellbeing	Maternal, newborn and child health	Child Health/Wellbeing<br><br>Maternal Health/Wellbeing<br><br>Newborn Health/Wellbeing or Stillbirths	Quasi-experimental	45 participants (e.g., research team members, project managers or steering committee members, government health agency members, NGO and charity members)	Education<br><br>Environmental Restructuring		Positive
Uskun et al., 2008[85]	Turkey	Middle	To improve healthcare providers’ knowledge of immunization and increase immunization coverage through an education intervention	Childhood vaccination	Child Health/Wellbeing	Quasi-experimental	229 primary healthcare workers (89 physicians,88 midwives, 38 health officers, 14 nurse)	Education		Positive
Vlad et al., 2016[86]	India	Middle	To describe a multi-stakeholder partnership to develop comprehensive clinical guidelines to reduce maternal mortality	Postpartum haemorrhage	Maternal Health/Wellbeing	Observational	400 healthcare staff working in 8 maternity wards	Environmental Restructuring<br><br>Training<br><br>Enablement	Environmental/ Social Planning	Positive
Warren et al., 2010[87]	Kenya	Middle	To evaluate the impact of a new postnatal intervention to improve healthcare workers’ counselling on maternal and newborn health	Maternal and newborn health	Maternal Health/Wellbeing<br><br>Newborn Health/Wellbeing or Stillbirths	Quasi-experimental	76 healthcare providers from four health facilities	Education	Service Provision	Positive
Wolfe et al., 2014[88]	USA	High	To develop evidence-based guidelines and complementary training for healthcare providers to support sharing life-altering information in pediatric settings	Health communication	Child Health/Wellbeing	Quasi-experimental	142 healthcare providers (54 physicians, 32 fellows and residents, 18 medical students, 14 health assistants, 15 nurses, 6 physiotherapists, 3 social workers)	Education<br><br>Environmental Restructuring	Guidelines<br><br>Environmental/ Social planning	Positive

Wolfenden et al., 2017[89]	Australia	High	To evaluate the implementation and compliance with a healthy school canteen policy, nutrient quality of foods provided at the canteens	Childhood nutrition	Child Health/Wellbeing	Experimental	70 primary schools with canteens (35 assigned to both intervention and control groups)	Education<br><br>Environmental Restructuring<br><br>Training<br><br>Enablement<br><br>Persuasion<br><br>Incentivisation	Communication/ Marketing<br><br>Environmental/ Social Planning	Positive
Yoong et al., 2016[90]	Australia	High	This study aimed to examine the impact of providing printed educational materials on childcare service cooks' intentions to use nutritional guidelines and provide fruit and vegetables on their menu	Childhood nutrition	Child Health/Wellbeing	Experimental	77 childcare centres (38 acted as the control and 39 participated in the intervention)	Education	Communication/ Marketing	Positive

References

1. Abney-Roberts, S. E. A Successful Quality Improvement Project to Improve Infant Safe Sleep Practice. *J. Obstet. Gynecol. Neonatal Nurs.* **44**, S43 (2015).
2. Agapidaki, E. *et al.* A theory-based educational intervention to pediatricians in order to improve identification and referral of maternal depression: a quasi-experimental study. *Ann. Gen. Psychiatry* **12**, 37 (2013).
3. Agency for Healthcare Research and Quality. *AHRQ Safety Program for Perinatal Care: Experiences From the Frontline. Prepared under contract no. 2902010000241 (RTI International). AHRQ Publication No. 17-0003-23-EF. Rockville, MD: Agency for Healthcare Research and Quality. October 30, 2016. www.ahrq.gov/perinatalsafety.* (2016).
4. Ainsworth, R. M., Mog, C. & Summerlin-Long, S. A Comprehensive Newborn Falls Initiative One Year Later. *J. Obstet. Gynecol. Neonatal Nurs.* **43**, S66 (2014).
5. Akter, S. F. U., Heller, R. D., Smith, A. J. & Milly, A. F. Impact of a training intervention on use of antimicrobials in teaching hospitals. *J. Infect. Dev. Ctries.* **3**, 447–451 (2009).
6. Al-Rafay, S. S. & Al-Sharkawy, S. S. Educational outcomes associated with providing a comprehensive guidelines program about nursing care of preterm neonates receiving total parenteral nutrition. *Clin. Nurs. Res.* **21**, 142–158 (2012).
7. Alkon, A. *et al.* Nutrition and physical activity randomized control trial in child care centers improves knowledge, policies, and children's body mass index. *BMC Public Health* **14**, 215 (2014).
8. Allen, C. W. & Jeffery, H. Implementation and evaluation of a neonatal educational program in rural Nepal. *J. Trop. Pediatr.* **52**, 218–222 (2006).
9. Allen, M. & Schafer, D. J. Nurses Improving the Health of Mothers and Infants by Dancing the 10 Steps to Successful Breastfeeding. *J. Obstet. Gynecol. Neonatal Nurs.* **44**, S52–S52 (2015).
10. Alton, M., Frush, K., Brandon, D. & Mericle, J. Development and implementation of a pediatric patient safety program. *Adv. Neonatal Care Off. J. Natl. Assoc. Neonatal Nurses* **6**, 104–111 (2006).
11. Alvarez, E. *et al.* Developing evidence briefs for policy: a qualitative case study comparing the process of using a guidance-contextualization workbook in Peru and Uganda. *Health Res. Policy Syst.* **17**, 89 (2019).
12. Anaby, D., Korner-Bitensky, N., Law, M. & Cormier, I. Focus on participation for children and youth with disabilities: Supporting therapy practice through a guided knowledge translation process. *Br. J. Occup. Ther.* **78**, 440–449 (2015).
13. Ansbro, É. M. *et al.* Introduction of Syphilis Point-of-Care Tests, from Pilot Study to National Programme Implementation in Zambia: A Qualitative Study of Healthcare Workers' Perspectives on Testing, Training and Quality Assurance. *PloS One* **10**, e0127728 (2015).
14. Baer, V. L. *et al.* Implementing a program to improve compliance with neonatal intensive care unit transfusion guidelines was accompanied by a reduction in transfusion rate: a pre-post analysis within a multihospital health care system. *Transfusion (Paris)* **51**, 264–269 (2011).
15. Barnard, J. G. *et al.* Facilitators and barriers to the use of standing orders for vaccination in obstetrics and gynecology settings. *Am. J. Obstet. Gynecol.* **216**, 69.e1-69.e7 (2017).
16. Becker-Haimes, E. M., Franklin, M., Bodie, J. & Beidas, R. S. Feasibility and Acceptability of a Toolkit to Facilitate Clinician Use of Exposure Therapy for Youth. *Evid.-Based Pract. Child Adolesc. Ment. Health* **2**, 165–178 (2017).
17. Berglund, A., Lefevre-Cholay, H., Bacci, A., Blyumina, A. & Lindmark, G. Successful implementation of evidence-based routines in Ukrainian maternities. *Acta Obstet. Gynecol. Scand.* **89**, 230–237 (2010).

18. Boyko, J. A., Kothari, A. & Wathen, C. N. Moving knowledge about family violence into public health policy and practice: a mixed method study of a deliberative dialogue. *Health Res. Policy Syst.* **14**, 31 (2016).
19. Braddick, L. *et al.* A mixed-methods study of barriers and facilitators to the implementation of postpartum hemorrhage guidelines in Uganda. *Int. J. Gynaecol. Obstet. Off. Organ Int. Fed. Gynaecol. Obstet.* **132**, 89–93 (2016).
20. Brennan, M. M. *et al.* Paediatric resuscitation for nurses working in Ghana: an educational intervention. *Int. Nurs. Rev.* **60**, 136–143 (2013).
21. Brown, J. J., Wacogne, I., Fleckney, S., Jones, L. & Bhrolchain, C. N. Achieving early surgery for undescended testes: quality improvement through a multifaceted approach to guideline implementation. *Child Care Health Dev.* **31**, 119–119 (2005).
22. Burgoine, K. *et al.* Staged implementation of a two-tiered hospital-based neonatal care package in a resource-limited setting in Eastern Uganda. *BMJ Glob. Health* **3**, (2018).
23. Cameron, D., Russell, D. J., Rivard, L., Darrah, J. & Palisano, R. Knowledge brokering in children's rehabilitation organizations: perspectives from administrators. *J. Contin. Educ. Health Prof.* **31**, 28–33 (2011).
24. Carlo, W. A. *et al.* Educational impact of the neonatal resuscitation program in low-risk delivery centers in a developing country. *J. Pediatr.* **154**, 504-508.e5 (2009).
25. Carmona J.M.; Howe E.; Zapata R.; Stevens D.; Murphy J.K. The teen health improvement program: A system-wide initiative to improve the quality of care for adolescents served by the country's largest public hospital system. in (2015).
26. Close, K., Karel, M. & White, M. A pilot program of knowledge translation and implementation for newborn resuscitation using US Peace Corps Volunteers in rural Madagascar. *Glob. Health* **12**, (2016).
27. Crone, M. R. *et al.* Sustainability of the Prevention of Passive Infant Smoking Within Well-Baby Clinics. *Health Educ. Behav.* **33**, 178–196 (2006).
28. Cunningham, B. J. & Cardy, J. O. Using implementation science to engage stakeholders and improve outcome measurement in a preschool speech-language service system. *Speech Lang. Hear.* **23**, 17–24 (2020).
29. Daniels, K., Lewin, S., & Practice Policy Group. Translating research into maternal health care policy: a qualitative case study of the use of evidence in policies for the treatment of eclampsia and pre-eclampsia in South Africa. *Health Res. Policy Syst.* **6**, 12 (2008).
30. Davies, B. *et al.* Fetal health surveillance: a community-wide approach versus a tailored intervention for the implementation of clinical practice guidelines. *CMAJ Can. Med. Assoc. J. J. Assoc. Medicale Can.* **167**, 469–474 (2002).
31. Deorari, A. K., Paul, V. K., Singh, M., Vidyasagar, D., & Medical Colleges Network. Impact of education and training on neonatal resuscitation practices in 14 teaching hospitals in India. *Ann. Trop. Paediatr.* **21**, 29–33 (2001).
32. Di Noia, J., Schwinn, T. M., Dastur, Z. A. & Schinke, S. P. The relative efficacy of pamphlets, CD-ROM, and the Internet for disseminating adolescent drug abuse prevention programs: an exploratory study. *Prev. Med.* **37**, 646–653 (2003).
33. Ding, H. *et al.* Influencing the use of antibiotics in a Chinese pediatric intensive care unit. *Pharm. World Sci. PWS* **30**, 787–793 (2008).
34. Dobbins, M. *et al.* A randomized controlled trial evaluating the impact of knowledge translation and exchange strategies. *Implement. Sci.* **4**, 61 (2009).
35. Edwards, N. *et al.* The impact of leadership hubs on the uptake of evidence-informed nursing practices and workplace policies for HIV care: a quasi-experimental study in Jamaica, Kenya, Uganda and South Africa. *Implement. Sci. IS* **11**, 110 (2016).
36. English, K. C., Merzel, C. & Moon-Howard, J. Translating public health knowledge into practice: development of a lay health advisor perinatal tobacco cessation program. *J. Public Health Manag. Pract. JPHMP* **16**, E9–E19 (2010).

37. Eriksson, L., Bergström, A., Hoa, D. T. P., Nga, N. T. & Eldh, A. C. Sustainability of knowledge implementation in a low- and middle-income context: Experiences from a facilitation project in Vietnam targeting maternal and neonatal health. *PLOS ONE* **12**, e0182626 (2017).
38. Farner, R. *et al.* The nurse champion model for advancing newborn screening of critical congenital heart disease. *J. Obstet. Gynecol. Neonatal Nurs. JOGNN* **43**, 497–506 (2014).
39. Finch, M. *et al.* Impact of a population based intervention to increase the adoption of multiple physical activity practices in centre based childcare services: a quasi experimental, effectiveness study. *Int. J. Behav. Nutr. Phys. Act.* **9**, 101 (2012).
40. Finch, M. *et al.* Challenges of Increasing Childcare Center Compliance With Nutrition Guidelines: A Randomized Controlled Trial of an Intervention Providing Training, Written Menu Feedback, and Printed Resources. *Am. J. Health Promot. AJHP* **33**, 399–411 (2019).
41. George E.; Maloney F.; Kara N.; Bukoye B.; Benotti E.; Delaney M.M.; Nejad S.B.; Kumar N.D.; Semrau K. FACTORS DETERMINING THE UTILIZATION OF ANTENATAL CARE IN RURAL AND URBAN SETTINGS IN MOZAMBIQUE. *Int. J. Gynecol. Obstet.* **143**, 523 (2018).
42. Gera, R. *et al.* Implementation of "health systems approach" to improve vaccination at birth in institutional deliveries at public health facilities; experience from six states of India. *J. Fam. Med. Prim. Care* **8**, 1630–1636 (2019).
43. Gichane, M. W., Mutesa, M. & Chowa, G. Translating Evidence into Policy Change: Advocacy for Community-Based Distribution of Injectable Contraceptives in Zambia. *Glob. Soc. Welf.* **6**, 41–47 (2019).
44. Gilkey, M. B., Parks, M. J., Margolis, M. A., McRee, A.-L. & Terk, J. V. Implementing Evidence-Based Strategies to Improve HPV Vaccine Delivery. *Pediatrics* **144**, (2019).
45. Goering, M. & Wilson, W. Implementing preterm labor guidelines: a collaborative care improvement process. *J. Perinat. Neonatal Nurs.* **16**, 47–57 (2002).
46. Goyet, S. *et al.* Knowledge translation: a case study on pneumonia research and clinical guidelines in a low- income country. *Implement. Sci.* **9**, 82 (2014).
47. Grady, A. *et al.* Effectiveness of a dissemination strategy on the uptake of an online menu planning program: A controlled trial. *Health Promot. J. Aust. Off. J. Aust. Assoc. Health Promot. Prof.* **30 Suppl 1**, 20–25 (2019).
48. Guillory, C. *et al.* Texas Pulse Oximetry Project: A Multicenter Educational and Quality Improvement Project for Implementation of Critical Congenital Heart Disease Screening Using Pulse Oximetry. *Am. J. Perinatol.* **34**, 856–860 (2017).
49. Hardee, K. *et al.* Improving Voluntary, Rights-Based Family Planning: Experience From Nigeria And Uganda. *Open Access J. Contracept.* **10**, 55–67 (2019).
50. Hulton, L. *et al.* Using evidence to drive action: a 'revolution in accountability' to implement quality care for better maternal and newborn health in Africa. *Int. J. Gynaecol. Obstet. Off. Organ Int. Fed. Gynaecol. Obstet.* **127**, 96–101 (2014).
51. Jeffery, H. E. *et al.* The impact of evidence-based education on a perinatal capacity-building initiative in Macedonia. *Med. Educ.* **38**, 435–447 (2004).
52. Jones, C. O. H. *et al.* 'Even if you know everything you can forget': health worker perceptions of mobile phone text-messaging to improve malaria case-management in Kenya. *PloS One* **7**, e38636 (2012).
53. Kapungu, C. T. *et al.* A community-based continuum of care model for the prevention of postpartum hemorrhage in rural Ghana. *Int. J. Gynaecol. Obstet. Off. Organ Int. Fed. Gynaecol. Obstet.* **120**, 156–159 (2013).
54. Kingsnorth, S., Orava, T., Parker, K. & Milo-Manson, G. From knowledge translation theory to practice: developing an evidence to care hub in a pediatric rehabilitation setting. *Disabil. Rehabil.* **42**, 869–879 (2020).

55. Kraft, J. M. *et al.* Dissemination and use of WHO family planning guidance and tools: a qualitative assessment. *Health Res. Policy Syst.* **16**, 42 (2018).

56. Lang, J. M., Franks, R. P., Epstein, C., Stover, C. & Oliver, J. A. Statewide dissemination of an evidence-based practice using Breakthrough Series Collaboratives. *Child. Youth Serv. Rev.* **55**, 201–209 (2015).

57. Makkar, S. R., Howe, M., Williamson, A. & Gilham, F. Impact of tailored blogs and content on usage of Web CIPHER - an online platform to help policymakers better engage with evidence from research. *Health Res. Policy Syst.* **14**, 85 (2016).

58. Margolis, P. A. *et al.* From concept to application: the impact of a community-wide intervention to improve the delivery of preventive services to children. *Pediatrics* **108**, E42 (2001).

59. Mello, M. J. *et al.* Translation of alcohol screening and brief intervention guidelines to pediatric trauma centers. *J. Trauma Acute Care Surg.* **75**, S301-307 (2013).

60. Melnyk, B. M. *et al.* Translating the evidence-based NICU COPE program for parents of premature infants into clinical practice: impact on nurses' evidence-based practice and lessons learned. *J. Perinat. Neonatal Nurs.* **24**, 74–80 (2010).

61. Mody, S. K., Ba-Thike, K. & Gaffield, M. E. The World Health Organization-United Nations Population Fund Strategic Partnership Programme's implementation of family planning guidelines and tools in Asia-Pacific countries. *J. Obstet. Gynaecol. Res.* **39**, 825–830 (2013).

62. Okonofua, F., Lambo, E., Okeibunor, J. & Agholor, K. Advocacy for free maternal and child health care in Nigeria—Results and outcomes. *Health Policy* **99**, 131–138 (2011).

63. Ongolo-Zogo, P., Lavis, J. N., Tomson, G. & Sewankambo, N. K. Assessing the influence of knowledge translation platforms on health system policy processes to achieve the health millennium development goals in Cameroon and Uganda: a comparative case study. *Health Policy Plan.* **33**, 539–554 (2018).

64. Pariyo, G. W., Gouws, E., Bryce, J., Burnham, G., & Uganda IMCI Impact Study Team. Improving facility-based care for sick children in Uganda: training is not enough. *Health Policy Plan.* **20 Suppl 1**, i58–i68 (2005).

65. Rowe, A. K. *et al.* A multifaceted intervention to improve health worker adherence to integrated management of childhood illness guidelines in Benin. *Am. J. Public Health* **99**, 837–846 (2009).

66. Russell, D. J. *et al.* Using knowledge brokers to facilitate the uptake of pediatric measurement tools into clinical practice: a before-after intervention study. *Implement. Sci. IS* **5**, 92 (2010).

67. Schreiber, J., Stern, P., Marchetti, G. & Provident, I. Strategies to promote evidence-based practice in pediatric physical therapy: a formative evaluation pilot project. *Phys. Ther.* **89**, 918–933 (2009).

68. Schreiber, J., Marchetti, G. F., Racicot, B. & Kaminski, E. The use of a knowledge translation program to increase use of standardized outcome measures in an outpatient pediatric physical therapy clinic: administrative case report. *Phys. Ther.* **95**, 613–629 (2015).

69. Schwartz, R. *et al.* Washington 'Steps' Up: A 10-Step Quality Improvement Initiative to Optimize Breastfeeding Support in Community Health Centers. *J. Hum. Lact. Off. J. Int. Lact. Consult. Assoc.* **31**, 651–659 (2015).

70. Segre, L. S., Brock, R. L., O'Hara, M. W., Gorman, L. L. & Engeldinger, J. Disseminating perinatal depression screening as a public health initiative: a train-the-trainer approach. *Matern. Child Health J.* **15**, 814–821 (2011).

71. Segre, L. S., Trusty, S., Gullickson, R., Chuffo Davila, R. & O'Hara, M. W. Brokering the Evidence-Practice Gap: A Strategy for Moving Evidence Into Clinical Practice. *Psychiatr. Serv. Wash. DC* **69**, 852–854 (2018).

72. Senarath, U., Fernando, D. N. & Rodrigo, I. Effect of training for care providers on practice of essential newborn care in hospitals in Sri Lanka. *J. Obstet. Gynecol. Neonatal Nurs. JOGNN* **36**, 531–541 (2007).

73. Seward, K. *et al.* Improving the implementation of nutrition guidelines in childcare centres improves child dietary intake: findings of a randomised trial of an implementation intervention. *Public Health Nutr.* **21**, 607–617 (2018).

74. Simioni, A. T. *et al.* [Regionalization of perinatal health care in the province of Santa Fe, Argentina]. *Rev. Panam. Salud Publica Pan Am. J. Public Health* **41**, e38 (2017).

75. R Simmons, Fajans, P. & Ghiron, L. Chapter 2: Strategic choices in scaling up: introducing injectable contraception and improving quality of care in Viet Nam. in *Scaling up health service delivery: from pilot innovations to policies and programmes* (World health Organization, 2007).

76. R Simmons, Fajans, P. & Ghiron, L. Chapter 7 Scaling up family planning service innovations in Brazil: the influence of politics and decentralization. in *Scaling up health service delivery: from pilot innovations to policies and programmes* (World health Organization, 2007).

77. Singh, K. *et al.* Impact evaluation of a quality improvement intervention on maternal and child health outcomes in Northern Ghana: early assessment of a national scale-up project. *Int. J. Qual. Health Care* **25**, 477–487 (2013).

78. Snelgrove-Clarke, E. E. The effects of action learning on nurses' use of a fetal health surveillance guideline with low-risk labouring women. https://escholarship.mcgill.ca/concern/theses/dz010q44w.

79. Sobel, H. L. *et al.* Implementing a national policy for hepatitis B birth dose vaccination in Philippines: Lessons for improved delivery. *Vaccine* **29**, 941–945 (2011).

80. Sundaram, A., Juarez, F., Ahiadeke, C., Bankole, A. & Blades, N. The impact of Ghana's R3M programme on the provision of safe abortions and postabortion care. *Health Policy Plan.* **30**, 1017–1031 (2015).

81. Tarasoff, L. A., Epstein, R., Green, D. C., Anderson, S. & Ross, L. E. Using interactive theatre to help fertility providers better understand sexual and gender minority patients. *Med. Humanit.* **40**, 135–141 (2014).

82. Twum-Danso, N. A. *et al.* Using quality improvement methods to test and scale up a new national policy on early post-natal care in Ghana. *Health Policy Plan.* **29**, 622–632 (2014).

83. Uneke, C. J., Sombie, I., Uro-Chukwu, H. C., Johnson, E. & Okonofua, F. Using equitable impact sensitive tool (EQUIST) and knowledge translation to promote evidence to policy link in maternal and child health: report of first EQUIST training workshop in Nigeria. *Pan Afr. Med. J.* **28**, 37 (2017).

84. Uneke, C. J., Sombie, I., Uro-Chukwu, H. C. & Johnson, E. Using equitable impact sensitive tool (EQUIST) to promote implementation of evidence informed policymaking to improve maternal and child health outcomes: a focus on six West African Countries. *Glob. Health* **14**, 104 (2018).

85. Uskun, E., Uskun, S. B., Uysalgenc, M. & Yagiz, M. Effectiveness of a training intervention on immunization to increase knowledge of primary healthcare workers and vaccination coverage rates. *Public Health* **122**, 949–958 (2008).

86. Vlad, I. *et al.* Improving quality for maternal care - a case study from Kerala, India. *F1000Research* **5**, (2016).

87. Warren, C., Mwangi, A., Oweya, E., Kamunya, R. & Koskei, N. Safeguarding maternal and newborn health: improving the quality of postnatal care in Kenya. *Int. J. Qual. Health Care* **22**, 24–30 (2010).

88. Wolfe, A. D. *et al.* Sharing life-altering information: development of pediatric hospital guidelines and team training. *J. Palliat. Med.* **17**, 1011–1018 (2014).

89. Wolfenden, L. *et al.* Multi-strategic intervention to enhance implementation of healthy canteen policy: a randomised controlled trial. *Implement. Sci. IS* **12**, (2017).

90. Yoong, S. L. *et al.* A theory-based evaluation of a dissemination intervention to improve childcare cooks' intentions to implement nutritional guidelines on their menus. *Implement. Sci.* **11**, 105 (2016).
