# Supplementary material for: Knowledge translation strategies for policy and action focused on sexual, reproductive, maternal, newborn, child and adolescent health and well-being: a rapid scoping review
Source: BMJ Open. 2022 Jan 17;12(1):e053919. doi: 10.1136/bmjopen-2021-053919 (PMC8765012; doi:10.1136/bmjopen-2021-053919)
Supplement: Author's manuscript [file bmjopen-2021-053919.draft_revisions.pdf]

BMJ Open

BMJ Open is committed to open peer review. As part of this commitment we make the peer review history of every article we publish publicly available.

When an article is published we post the peer reviewers' comments and the authors' responses online. We also post the versions of the paper that were used during peer review. These are the versions that the peer review comments apply to.

The versions of the paper that follow are the versions that were submitted during the peer review process. They are not the versions of record or the final published versions. They should not be cited or distributed as the published version of this manuscript.

BMJ Open is an open access journal and the full, final, typeset and author-corrected version of record of the manuscript is available on our site with no access controls, subscription charges or pay-per-view fees (http://bmjopen.bmj.com).

If you have any questions on BMJ Open's open peer review process please email info.bmjopen@bmj.com

BMJ Open

Effective Knowledge Translation Strategies for Policy and Action Focused on Sexual, Reproductive, Maternal, Newborn, Child & Adolescent Health and Wellbeing: A Rapid Scoping Review

Journal:	*BMJ Open*
Manuscript ID	bmjopen-2021-053919
Article Type:	Original research
Date Submitted by the Author:	30-May-2021
Complete List of Authors:	Curran, Janet; Dalhousie University, Nursing; IWK Health Centre, Pediatrics<br>Gallant, Allyson; Dalhousie University, Faculty of Health<br>Wong, Helen ; Dalhousie University, Faculty of Health<br>Shin, Hwayeon Danielle; Dalhousie University, School of Nursing<br>Urquhart, Robin; Dalhousie University, Department of Community Health and Epidemiology<br>Kontak, Julia; Maritime SPOR SUPPORT Unit<br>Wozney, Lori; Nova Scotia Health<br>Boulos, Leah; Maritime SPOR SUPPORT Unit<br>Bhutta, Zulfiqar; The Hospital for Sick Children, Centre for Global Child Health<br>Langlois, Etienne V.; World Health Organization, The Partnership for Maternal, Newborn & Child Health
Keywords:	Community child health < PAEDIATRICS, Quality in health care < HEALTH SERVICES ADMINISTRATION & MANAGEMENT, PUBLIC HEALTH

SCHOLARONE™
Manuscripts

I, the Submitting Author has the right to grant and does grant on behalf of all authors of the Work (as defined in the below author licence), an exclusive licence and/or a non-exclusive licence for contributions from authors who are: i) UK Crown employees; ii) where BMJ has agreed a CC-BY licence shall apply, and/or iii) in accordance with the terms applicable for US Federal Government officers or employees acting as part of their official duties; on a worldwide, perpetual, irrevocable, royalty-free basis to BMJ Publishing Group Ltd ("BMJ") its licensees and where the relevant Journal is co-owned by BMJ to the co-owners of the Journal, to publish the Work in this journal and any other BMJ products and to exploit all rights, as set out in our licence.

The Submitting Author accepts and understands that any supply made under these terms is made by BMJ to the Submitting Author unless you are acting as an employee on behalf of your employer or a postgraduate student of an affiliated institution which is paying any applicable article publishing charge ("APC") for Open Access articles. Where the Submitting Author wishes to make the Work available on an Open Access basis (and intends to pay the relevant APC), the terms of reuse of such Open Access shall be governed by a Creative Commons licence – details of these licences and which Creative Commons licence will apply to this Work are set out in our licence referred to above.

Other than as permitted in any relevant BMJ Author's Self Archiving Policies, I confirm this Work has not been accepted for publication elsewhere, is not being considered for publication elsewhere and does not duplicate material already published. I confirm all authors consent to publication of this Work and authorise the granting of this licence.

For peer review only

Effective Knowledge Translation Strategies for Policy and Action Focused on Sexual, Reproductive, Maternal, Newborn, Child & Adolescent Health and Wellbeing: A Rapid Scoping Review

Janet A. Curran*[1,2] (jacurran@dal.ca) *Corresponding author https://orcid.org/0000-0001-9977-0467

Allyson J. Gallant[3] https://orcid.org/0000-0002-2933-7470

Helen Wong[3] https://orcid.org/0000-0001-6827-0662

Hwayeon Danielle Shin[1] https://orcid.org/0000-0003-4037-4464

Robin Urquhart[4] https://orcid.org/0000-0001-8864-5716

Julia Kontak[5] https://orcid.org/0000-0002-9104-0678

Lori Wozney[6] https://orcid.org/0000-0003-4280-3322

Leah Boulos[5] https://orcid.org/0000-0002-9849-383X

Zulfiqar A. Bhutta[7] https://orcid.org/0000-0003-0637-599X

Etienne V. Langlois[8] https://orcid.org/0000-0002-4853-2511

AFFILIATIONS:

[1] School of Nursing, Dalhousie University, 5869 University Avenue, Halifax, Nova Scotia, Canada B3H 4R2

[2] IWK Health Centre, Halifax, Nova Scotia, Canada

[3] Faculty of Health, Dalhousie University, Halifax, Nova Scotia, Canada

[4] Department of Community Health and Epidemiology, Dalhousie University, Halifax, Nova Scotia, Canada

[5] Maritime SPOR SUPPORT Unit, Halifax, Nova Scotia, Canada

[6] Nova Scotia Health, Nova Scotia, Canada

For peer review only

[7] Centre for Global Child Health, The Hospital for Sick Children (SickKids), Toronto, Ontario, Canada

[8] The Partnership for Maternal, Newborn & Child Health (PMNCH), World Health Organisation, Geneva, Switzerland

For peer review only

ABSTRACT

Objective: The aim of this study was to identify knowledge translation (KT) strategies aimed at improving sexual, reproductive, maternal, newborn, child and adolescent health (SRMNCAH) and wellbeing.

Design: Rapid scoping review.

Search Strategy: A comprehensive and peer-reviewed search strategy was developed and applied to four electronic databases: MEDLINE ALL, Embase, CINAHL, and Web of Science. An additional search of grey literature was conducted to identify KT strategies aimed at supporting SRMNCAH. KT strategies and policies published in English from January 2000 onwards were eligible for inclusion.

Results: Only 4% of included 90 studies were conducted in low-income countries with the majority (52%) conducted in high-income countries. Studies primarily focused on maternal newborn or child health and wellbeing. *Education* (81%), including staff workshops and education modules, was the most commonly identified intervention component from the KT interventions. Low- and middle-income countries were more likely to include civil society organizations, government and policy makers as stakeholders compared to high-income countries. Reported barriers to KT strategies included limited resources and time constraints, while enablers included supportive stakeholder involvement throughout the KT process.

Conclusion: Gaps exist among KT strategies for SRMNCAH policy and action, including limited focus on adolescent, sexual and reproductive health and rights and SRMNCAH financing strategies. There is a need to support stakeholder engagement in KT interventions across the continuum of SRMNCAH services, especially in low-income countries. Future efforts in SRMNCAH should prioritize working with multi-sectoral stakeholders to implement successful KT strategies and policies.

Registration: The rapid scoping review protocol was registered on Open Science Framework

For peer review only

on June 16th, 2020 (https://osf.io/xpf2k).

KEYWORDS: knowledge translation, sexual health, reproductive health, maternal health, newborn health, child health, adolescent health

Strengths and Limitations

- Our comprehensive literature and grey review of the literature search identified a broad range of KT strategies and policies aimed at improving sexual, reproductive, maternal, newborn, child and adolescent health (SRMNCAH) and wellbeing published since January 2020
- KT strategies and policies were mapped to the Behaviour Change Wheel to identify and describe intervention and policy elements included in the strategies
- Narrative and tabular descriptions were produced to summarize KT strategies and policies, stakeholder engagement, outcome measures, and barriers and enablers
- The terminology around knowledge translation varies across countries and health areas

For peer review only

INTRODUCTION

Progress towards sexual, reproductive, maternal, newborn, child and adolescent health (SRMNCAH) has been highly inequitable to date.[1,2] With the current COVID-19 pandemic, there have been substantive and unprecedented disruptions in essential SRMNCAH services,[3] including emerging data on increased maternal mortality, stillbirth rates, ruptured ectopic pregnancies, unintended pregnancies, maternal depression and limited access to contraceptives.[4,5] The greatest disruptions to essential healthcare services are witnessed in low-income countries.[6] As such, special efforts are needed to support effective evidence-based interventions to prevent further harm, reduce preventable deaths and morbidity, and promote equitable distribution of essential interventions for SRMNCAH.

There is a lack of equitable distribution of human resources and essential policy adoptions for SRMNCAH in most countries.[7] COVID-19 has also brought to the forefront the need to develop and implement multi-sectoral interventions using a whole-of-government approach.[8]or Improving SRMNCAH across countries of different income levels will require collective action in terms of generating, sharing, brokering, and implementing new knowledge through cross-sectoral and interdisciplinary initiatives.[9] Interventions shown to be effective by scientific endeavours require efforts to integrate evidence into policy and action. Knowledge translation (KT) is "a dynamic and iterative process that includes synthesis, dissemination, exchange and ethically-sound application of knowledge to improve the health, provide more effective health services, and products and strengthen the healthcare system."[10] KT interventions can support this process by facilitating the uptake of evidence into policy and practice targeting change at the level professional, institutional or policy level. There has been a growing number of KT interventions, as well as frameworks,

For peer review only - http://bmjopen.bmj.com/site/about/guidelines.xhtml

theories, and models to guide the selection of KT interventions.[11–13] However, the range of effective KT strategies related to SRMNCAH improvements remains unknown.

No review to date has explored the range of KT interventions utilized at the level of health system, policy, or practice specifically addressing the continuum of SRMNCAH. In light of the global call for action to sustain SRMNCAH, it is critical to understand the effectiveness and implementation of KT strategies that promote evidence-based policy and practice for SRMNCAH.

Objectives:

The aim of this rapid scoping review was to identify existing literature related to KT strategies that promote the uptake of evidence into policy and action focused on improving SRMNCAH and wellbeing. To achieve this aim, four questions were addressed:

1. What are the common KT strategies and activities used to promote the use of evidence to inform policy and action to improve SRMNCAH and wellbeing?
2. How are stakeholders involved in designing or implementing these KT strategies and activities?
3. What are the commonly reported outcomes of KT strategies and activities to promote the use of evidence in SRMNCAH and wellbeing?
4. What are the commonly reported barriers and enablers for using KT strategies and activities to promote the use of evidence in SRMNCAH and wellbeing?

METHODS

This rapid scoping review follows the methodological guidance developed by the Joanna Briggs Institute and is reported according to the Preferred Reporting Items for

For peer review only - http://bmjopen.bmj.com/site/about/guidelines.xhtml

158 Systematic Reviews and Meta-Analyses - Extension for Scoping Reviews (PRISMA-ScR). [14,15]

159 The rapid scoping review protocol was registered on Open Science Framework on June 16th,

160 2020 (https://osf.io/xpf2k).

161 **Inclusion and Exclusion Criteria**

162 All study designs were eligible for inclusion in the review. Studies were excluded if

163 they focused on basic science or clinical management of women, newborn, child or

164 adolescent aspects of health or wellbeing. Systematic reviews were also excluded; however,

165 the reference lists of relevant reviews were examined to identify additional potential

166 studies for inclusion. Studies published from 2000 onwards and published in English were

167 eligible for inclusion.

168 Studies reporting a KT strategy aimed at supporting or improving health systems or

169 policy decisions to support SRMNCAH and wellbeing were eligible for inclusion. KT strategies

170 aimed at patients, caregivers, healthcare providers, healthcare management, health

171 systems, policy makers, civil society organizations and funder or donors, within or outside

172 the health sector, were also eligible as long as it was in the context of SRMNCAH and

173 wellbeing. Studies that targeted these stakeholders outside of SRMNCAH and wellbeing

174 were excluded. KT strategies addressing HIV were excluded, unless specifically focused on

175 SRMNCAH. Study outcomes relating to the effectiveness or implementation of the KT

176 strategy or activity and SRMNCAH were included. Studies that did not report primary

177 outcomes relevant to KT or SRMNCAH were excluded.

178 **Search Strategy and Information Sources**

179 A comprehensive search strategy was developed with support from an experienced

180 library scientist. The search strategy was peer-reviewed by a second library scientist using

181 the Peer Review of Electronic Search Strategy (PRESS) guidelines to ensure a comprehensive

For peer review only - http://bmjopen.bmj.com/site/about/guidelines.xhtml

and high-quality search strategy was developed.[16] An electronic database search of MEDLINE ALL (Ovid), Embase (Elsevier Embase.com), CINAHL with Full Text (EBSCOhost), and Web of Science (SCI-EXPANDED, SSCI, A&HCI, CPCI-S, CPCI-SSH, ESCI; Clarivate) was executed on May 29, 2020, and results were limited from January 2000 to the search date (see Supplementary File 1). No search filters or other limits were applied. Search strategy citations were imported into Covidence, an online systematic review management software, and duplicates were removed automatically in Covidence prior to screening.[17] Reference lists of all included studies, as well as those of any relevant systematic reviews, were screened by one reviewer and verified by a second. Additionally, a search of grey literature was undertaken by a reviewer in July 2020. Search terms were applied in Google and relevant website links were clicked through to identify any reports or literature. The reviewer clicked through each relevant website and used reference chaining within the website to ensure any and all relevant literature was identified. Google results were browsed until the reviewer went two pages (20 results) without clicking on a potentially relevant result. The website URL links were compiled and verified for inclusion by another team member.

Selection of Sources of Evidence

Reviewers independently screened titles and abstracts against the inclusion criteria, and all conflicts were resolved by a third reviewer. Full-text articles were then reviewed and assessed against the inclusion criteria by one reviewer then verified by the second reviewer. Uncertainties at this stage were resolved through discussions with the research team. The reference lists of included full-text articles were then reviewed to identify other potential studies for inclusion.

Data Charting & Data Items

For peer review only - http://bmjopen.bmj.com/site/about/guidelines.xhtml

Data was extracted and mapped to four categories: Descriptive details of the study (e.g., authors and year, country, sample characteristics, study design, decision-making level, and SRMNCAH priority), characteristics of KT strategy of activity, KT strategy details, mode of KT delivery, stakeholder involvement), outcomes and direction of effect, and identified barriers and enablers identified by study authors. Study data were extracted using the data extraction tool by one reviewer and was verified by another to ensure all relevant data were captured. Critical appraisal of individual sources of evidence were not conducted.

Synthesis of Results

Following data extraction, the income level of each country identified from the studies was determined using the World Bank classification.[18] Study details were grouped into four categories that aligned with each of the research questions: KT strategy, stakeholder engagement, reported outcomes, and types of barriers and enablers identified. Quantitative summaries and thematic analysis were then applied to each grouping to identify potential trends across country income levels and SRMNCAH thematic areas. Study data were also mapped on to the Partnership for Maternal, Newborn and Child Health (PMNCH) high-level outcomes of interest identified in the 2021-2025 Strategy (e.g., policy, service delivery, financing)[19] and narrative summaries were produced.

To facilitate summarizing content associated with the KT strategies, details of each KT strategy were mapped to the Behaviour Change Wheel (BCW).[20] The BCW provides a synthesis of 19 behaviour change theories in a comprehensive, theory-based tool that can be used to identify important behaviour change elements to consider in intervention design.[21] The BCW includes nine intervention functions that can be used to guide intervention content and design. It also includes seven policy categories to guide implementation of behaviour change interventions and policies. [20] KT strategies were

For peer review only

For peer review only - http://bmjopen.bmj.com/site/about/guidelines.xhtml

mapped to relevant BCW intervention functions and policy categories by two independent researchers. Researchers met to review BCW coding and discrepancies were resolved through discussion.

Patient and Public Involvement

This scoping review was conducted without the involvement of patients or members of the public.

RESULTS

Selection of Sources of Evidence

The search strategy returned 11,190 studies for screening. After removing 3,626 duplicates, 7,564 titles and abstracts were screened by reviewers. This stage identified 212 full-text studies to review. Following full-text analysis, 154 studies were excluded, resulting in 58 studies included in the review. A review of the reference lists of included studies identified 26 additional studies that met the inclusion criteria. The grey literature search identified one study for inclusion; five additional studies were included from screening the reference list of a relevant systematic review. This resulted in a final total of 90 included studies in the scoping review. The selection process and sources of evidence are summarized in a PRISMA-ScR flow diagram (Figure 1).[22]

* Insert Figure 1*

Characteristics of Sources of Evidence

A summary of the characteristics of the 90 included studies can be found in Table 1, with a comprehensive description of studies found in Supplementary File 2. All studies were published between 2000-2020, with an increase in relevant publications since 2006. Just

over a third (34%) where published between 2011-2015[23–53] and another 34% between 2016-2020.[54–84] Thirty-two percent of studies were quasi-experimental designs,[23,25,26,28,31,32,34,35,44,50,51,59–61,67,68,75,76,83,85–92] with observational (22%),[36,37,39,43,45,52,71,72,77,80,93–102] mixed methods (17%),[40–42,47,53,56–58,64,69,82,103–106] experimental (16%),[24,27,38,62,66,74,78,79,107–112] and qualitative (11%)[29,30,33,49,54,55,63,70,81,113] designs also identified. Two editorials were also included.[65,73]

Country Income Levels

The majority of the studies (52%) were conducted in high-income countries,[23–25,27–29,31,33–35,38,40,41,43–45,49,53,56,57,62,66–68,71,73,74,74,80,83,87,93–95,97,98,100,102–105,107,108,108,109,112] including Canada, the United States, and Australia. Middle-income countries were the setting for 36% studies,[26,30,32,36,39,42,46–48,50,52,63–65,75–77,81,82,85,86,88–92,96,101,110,111,113] including Nepal, Egypt, and Zambia, with most middle-income countries considered low-middle income (81%) and 19% considered upper-middle income. Only 4% of included studies were located in low-income countries, such as Uganda and Madagascar [58–60,99]. Seven countries (i.e., Bangladesh, Cameroon, Ethiopia, Myanmar, Nigeria, Solomon Islands and Uganda) across 14% of studies represented humanitarian or highly fragile settings,[37,42,54,58,59,61,70,72,85,99] according to Organisation for Economic Co-operation and Development.[114] Eight percent of studies included multi-income countries, with six targeting both low- and middle-income countries (e.g., Uganda and Peru), [37,54,61,69,70,72] and one targeting middle and high-income countries (e.g., Brazil and Chile).[106] As results were reported by country in these seven studies, we analyzed these studies at each of the appropriate income levels.

SRMNCAH priorities

Child health and wellbeing was the most commonly identified priority, with 30%

targeting these concerns.[27,29,32,33,35,36,38,40,43,46,47,51,57,62,67,74,78–80,83,85,89,91,94,98–100,105,108,110] Twenty-eight percent addressed newborn health and wellbeing or stillbirths,[23,25,26,28,31,34,50,59,60,60,64,68,81,82,86,88,90,95–97,109,111] and a quarter of studies addressed maternal health and wellbeing.[24,37,39,44,45,52,54,55,58,63,72,73,75–77,87,92,93,103,104,112,113] Studies addressing sexual and reproductive health and rights (SRHR [12%]) [30,38,42,61,65,66,69–71,101,106] and adolescent health and wellbeing (4%)[33,41,56,107] priorities were identified, but in a lower volume compared to studies addressing maternal, child and newborn or stillbirth priorities. Some studies (13%) also included multiple SRMNCAH priorities, with eight targeting both maternal and newborn health and wellbeing,[37,46,54,81,82,84,90,92] two targeting maternal and child health and wellbeing,[52,72] and two targeting maternal, child and newborn health and wellbeing.[75,76] These studies were analyzed at each of the appropriate priority levels.

PMNCH High-Level Outcomes (2021-2025)

Across the priority high-level outcomes of the PMNCH 2021-2025 Strategy, the majority of included studies (81%) addressed *service delivery*. *Policymaking* was also commonly identified (19%). None of the included KT strategies addressed *financing* as a central focus.

Settings

KT strategies were primarily implemented in hospitals (39%), community (25%) or primary care (8%) settings. Three percent of strategies were conducted within government health departments or agencies.[71,73,108] There were rarely enough details provided to explore specific departmental settings in hospitals, although four hospital studies were conducted in newborn and pediatric intensive care units (NICUs/PICUs),[26,31,89,109] one in a labour and delivery department,[93] and one specified the hospital was in a rural setting.[86] Community settings were also described in limited detail, however eight studies were

conducted in childcare centres,[27,35,38,62,67,74,79,83] two in schools,[78,107] and one in a mental health community clinic.[56]

Insert Table 1

Synthesis of KT Strategies and Activities:

BCW Intervention Functions: Mapping the KT strategies to the BCW intervention functions identified that all but one study contained at least one intervention function. All nine BCW intervention functions were identified across the KT strategies, *with Education, Training,* and *Environmental Restructuring* the most common (Table 2).

Education was the most commonly identified element from the included studies, identified in 81% of studies. Types of *Education* provided included modules, staff workshops, slides and other resources. This function was identified in 73 KT strategies across country income levels (88% in high-income countries, 80% of low-income countries, and 77% of middle-income countries). At the SRMNCAH priority level, 91% of child health and wellbeing strategy included an *Education* function. *Education* was also identified in 81% of the maternal health interventions, as well as 75% of each of the SRHR, and adolescent and wellbeing interventions.

Training was identified in 51% of strategies. *Training* typically included conveying skills to staff members and healthcare providers. This element was included in 60% of the KT strategies aimed at low-income countries, 52% of high-income countries and 41% of middle-income countries. Strategies stratified by SRMNCAH priority identified *Training* was utilized consistently across each priority.

Environmental Restructuring was identified in 50% of the KT strategies. This element included reorganizing how services were provided, how health centers were set up, and adding additional resources (e.g., tools, team members) in the health centre or health system to facilitate use of the KT strategy. This element was identified at all three income levels but was more likely to be used in high-income countries (58%), compared to middle (41%) and low-income (40%) countries. Sixty-nine percent of child health and wellbeing strategies included this function, as well as 50% of the sexual and reproductive health and rights strategies. *Environmental Restructuring* was only applied in one of the four (25%) adolescent health and wellbeing strategies.

BCW Policy Categories: Sixty percent of studies included policy category content, with six of the seven BCW policy categories were identified across the strategies (Table 2). No strategies applied content related to the *Fiscal* category option. *Guidelines* and *Service Provision* were the most commonly identified, with *Guidelines* identified in 28% of KT strategies and *Service Provision* identified in 20% of studies. *Guidelines* were implemented in 50% of the low-income countries, compared to only 31% of middle-income and 25% of the high-income countries. *Guidelines* were most frequently used in 50% of KT strategies related to sexual or reproductive health and rights, and 36% of maternal health and well-being strategies.

Service Provision was implemented in 89% of the high-income countries, compared to only 5% of middle-income countries and none of the low-income countries. This policy category was divided across SRMNCAH priorities. SRHR included *Service Provision* in 25% of strategies, and 25% of strategies targeting adolescent health and wellbeing included this category.

For peer review only

Insert Table 2

Mode of KT Delivery: Seventy-two percent of KT strategies identified the mode of KT delivery, with the majority using one mode of delivery, while 27% used two or more modes. The majority of these studies used in-person delivery (59%) as the sole mode of delivery. Of the multi-modal delivery strategies, in-person delivery was also included as a mode in all but one study.[111] High-income countries used more multi-mode interventions, such as in-person with additional online or web-based components, compared to those in low- and middle-income countries. Studies published between 2018-2020 have used evolving trends in technology, such as webinars and social media,[73,83] as modes of delivery.

Synthesis of Stakeholder Involvement

There was an overall lack of description provided on how stakeholders were involved in designing or implementing KT strategies, with 31% of studies not providing any description of stakeholder engagement. Of the remaining studies that did provide details, the level of detail varied by article, with some simply acknowledging stakeholders were engaged, while others provided a more comprehensive view of the stakeholder groups involved and their roles. Commonly identified stakeholder groups included: government and policy makers, healthcare providers, civil society organizations, members of the public, and members of the research community (Table 3). Engagement with these groups was distributed across country income level, with two notable exceptions. First, civil society organizations were more likely to be engaged in low (60%) and middle-income (48%) countries compared to high-income (27%) countries. Additionally, government and policymakers were engaged by low (50%) and middle-income (62%) countries much more often than in high-income (8%) countries.

For peer review only

Stakeholder engagement was dispersed across the SRMNCAH priorities. Priorities addressing SRHR were more likely to include policymakers and government as well as civil society organizations compared to other priorities. Half of strategies addressing adolescent health and wellbeing, while 25% of SRHR engaged healthcare providers including clinicians, nurses and allied healthcare professionals. Involvement of researcher communities was identified across all six priorities but was rarely used in newborn health and wellbeing or stillbirth strategies (7%).

Insert Table 3

Synthesis of Outcomes:

Nearly 80% of KT strategy outcomes were reported at only a single outcome level (e.g., patient or healthcare provider), with 20% studies reporting multiple outcome levels (e.g., healthcare provider and system). At the single outcome level, 38% of outcomes were measured at the healthcare provider level (e.g., increased knowledge) and 29% at system level (e.g., reductions in safety incidents). Only 5% of strategies included patient level outcomes (e.g., improved newborn sleep). Patient outcomes were more likely to be included in multi-level outcomes, along with healthcare provider and system level (e.g., immunization rates, quality of care provided and healthcare system use) outcomes. Of the multi-level outcome studies, 17% had outcomes at two levels (e.g., patient and healthcare providers, healthcare providers and system, or patient and system) and 4% of studies included three level outcomes (e.g., patient, healthcare provider and system).

Healthcare provider outcomes were mostly reported in high-income (44%) and low-income (40%) countries, compared to 26% of middle-income countries, while system level outcomes were more common in low-income (40%) and middle-income (41%) countries. Healthcare provider outcomes were identified across the SRMNCAH priorities, ranging from

For peer review only

23-75% for maternal health and wellbeing strategies to those for adolescent wellbeing, respectively. While fewer maternal health and wellbeing strategies included healthcare provider level outcomes, nearly half of these strategies (48%) were aimed at system level outcomes. Childhood health and wellbeing strategies were also most likely to address multi-level outcomes compared to all other priorities.

Synthesis of Barriers and Enablers:

Few studies reported barriers and enablers to using KT strategies to promote the use of evidence in SRMNCAH and wellbeing. Fewer than half of the studies (43%) outlined barriers and even fewer identified enablers (40%) to their KT strategies. When studies included barriers and enablers, the level of detail provided varied across the studies, with some studies providing a brief list of these factors while other studies provided more detailed descriptions and how each affected the KT strategy. A summary of identified barriers and enablers can be found in Table 4.

Identified Barriers: Limited resources was the most commonly reported barrier for using KT strategies across countries of all income levels and SRMNCAH priorities. In 56% of strategies, limited resources referred to physical (e.g., funding) or human resources (e.g., healthcare staff) constraints. Low-income countries were more likely to report limited resources (40%) compared to high-income countries (19%). In terms of SRMNCAH priority, 50% of SRHR strategies identified limited resources as barriers to using KT strategies.

Secondly, time constraints were reported in 21% of high-income countries. This type of barrier delayed the implementation process, including fidelity of KT strategies. Lastly, negative attitudes was the third commonly reported barrier, reported by 30% of low-

For peer review only - http://bmjopen.bmj.com/site/about/guidelines.xhtml

For peer review only

income countries. Examples of negative attitudes included resistance to change, lack of confidence, and poor 'buy-in' for using KT strategies.

Identified Enablers: Supportive stakeholder involvement was the most commonly identified enabler to KT strategies. Two-thirds of studies which reported enablers identified the importance of developing supportive relationships with stakeholders, and that the partnerships forged supported the implementation of the KT strategy. Supportive stakeholder involvement included successful collaboration and partnerships with, but not limited to, healthcare providers, government bodies, or non-profit organizations. This enabler was identified across all country income levels but was most common in low-income countries (40%). In terms of SRMNCAH priority, 67% of SRHR studies reported supportive stakeholder involvement as enablers.

Secondly, access to resources and knowledge were the next commonly reported enablers for using KT strategies. Resources in these articles included financial as well as human resources. This enabler was more common in low-income countries (20%) compared to middle-income (8%) or high-income (5%) countries. This enabler was identified across the SRMNCAH priorities, with the exception of adolescents' health and well-being. Finally, only high-income countries reported skills and training as enablers for using KT strategies.

Insert Table 4

DISCUSSION

This rapid scoping review identified 90 studies published since 2000 which utilized KT strategies and policies to support the use of evidence for improving SRMNCAH. While a wide range of studies across country income levels and SRMNCAH priorities were identified, most KT strategies were implemented in high-income countries and focused on maternal, newborn or stillbirths, or child health and wellbeing topics. The review identified key gaps in

For peer review only

For peer review only - http://bmjopen.bmj.com/site/about/guidelines.xhtml

effective KT interventions to support evidence-based decision-making for SRHR, adolescent health and wellbeing and SRMNCAH financing. Our findings illustrate the majority of KT strategies included an *education* component and strategies were commonly aimed at addressing healthcare provider and system-level level outcomes. Across PMNCH outcomes of interest, most strategies address *service delivery* or *policymaking,* with none addressing SRMNCAH financing. While few details were typically provided on stakeholder engagement, or on barriers and enablers in the KT process, it was noted that collaboration with stakeholders and building partnerships with local actors, such as government or health authorities, facilitated use of KT strategies.

Low- and middle-income countries identified a lack of resources (e.g., funding, staff, physical resources) as the most common barrier to implementing and sustaining KT strategies. While lack of resources were also identified in high-income countries, these barriers look different based on country income level and are more often pronounced in low- and middle-income countries.[113] As only ten of the identified KT strategies were implemented and evaluated in low-income countries, this may suggest more work is needed in these countries to move evidence into practice to improve SRMNCAH outcomes while addressing the economic, resource, and health system barriers they experience.[115] Using a tailored approach to specifically address the unique barriers in low-income countries may help support the successful implementation of KT strategies and improve maternal, child and adolescent health outcomes within these countries.[115]

Engaging with stakeholders was viewed as a key enabler to KT strategies included in this review. Among the groups of stakeholders involved, low- and middle-income countries were more likely to include members of civil societies and non-governmental organizations

For peer review only - http://bmjopen.bmj.com/site/about/guidelines.xhtml

compared to high-income countries. Additionally, government and policymakers were engaged by low- and middle-income countries much more often than in high-income countries. Involving civil societies in knowledge translation, especially among low-income countries, is crucial as these organizations often have the capacity and resources required to implement recommendations.[116] Drawing on civil society stakeholder groups throughout the design and implementation of KT strategies may provide vital support to help facilitate the implementation and evaluation of KT strategies.

SRMNCAH and Wellbeing

Maternal health and wellbeing strategies addressed pre-term labour management, labour and delivery outcomes and perinatal care, although primarily in high-income countries.[63,84,102,103,112] These strategies typically included utilizing clinical guidelines and education for nurses to help support improvements in these maternal outcomes. Our review has identified a critical gap around effective KT approaches to support evidence based SRMNCAH interventions in low-income countries. Yet, these women and communities experience more barriers to high quality healthcare, including trained healthcare providers during childbirth.[117,118] More investments and capacity strengthening efforts are needed to support KT interventions in low-income settings, thus advancing evidence-based policy and practice in settings where the needs are the most dire. This includes humanitarian and fragile settings, which bear a disproportionate burden of poor SRMNCAH outcomes. For instance, maternal mortality increases by 11% on average in conflict zones and by 28% in the worst-hit areas.[119] Additionally, more than 10 million deaths in children younger than 5 years can be attributed to conflict between 1995 and

For peer review only - http://bmjopen.bmj.com/site/about/guidelines.xhtml

2015 globally.[119] In addition, up to a third of girls living in a humanitarian setting report that their first sexual encounter was forced.[120]

Many of the identified strategies were aimed at improving newborn, childhood and maternal mortality rates, particularly in low and middle-income countries.[23,37,52,59,72,81,86,90] Strategies included education sessions targeting healthcare providers to ensure quality care is provided to support health outcomes among these groups, and one strategy outlined an advocacy campaign targeting government members to support the provision of free health services for pregnant women and children to help reduce mortality rates.[52] Providing educational and training opportunities to healthcare providers is vital to targeting health system barriers often experienced in low- and middle-income countries.[118,121] However it is recommended to include stakeholder groups within and beyond the health system in these strategies, and these multi-sectoral stakeholder strategies will need to be sustainable to ensure continuous access to high quality healthcare for children.

Our review identified an overall lack of relevant KT strategies addressing adolescent health and wellbeing. This is a significant gap in the literature as adolescents represent over 1.2 billion of today's population, and 90% of these youth living in low and middle-income countries.[122,123] While the adolescent studies included in this review addressed issues including substance use, mental health and overall health needs,[41,53,56,107] there is a lack of research into supporting dietary and lifestyle choices and SRHR in this population. Focusing on developing KT programs and policies to address the health needs of adolescents is essential to supporting the transition into healthy adults. Future KT programs and policies should be co-designed with adolescents and youth to harness their capacity and advocacy skills and to ensure their unique health needs are being addressed.[123]

SRMNCAH Financing

Our review identified a key gap in effective KT interventions to support evidence-based decision-making on SRMNCAH financing. This finding highlights the need for greater efforts to ensure that robust health financing evidence is used to strengthen public financial management systems pertaining to SRMNCAH.[124] This is critical to support efficient spending in times of COVID-19, whereby governments are struggling with shrinking fiscal spaces and major disruptions to essential SRMNCAH services.[125]

Implications for Future KT Strategies

Findings from this scoping review identified educational interventions aimed at changing healthcare provider behaviours to improve provision of care are implemented across all countries and SRMNCAH priorities. Future work should build on these good practices to address issues around SRHR, adolescent health and wellbeing, and SRMNCAH financing. It will also be essential for teams designing and implementing KT strategies to integrate stakeholder groups early on in the process and co-design KT interventions to optimize the success of work.[126] Depending on the complexity and scalability of the KT strategies, especially in low and middle-income countries, there is an opportunity to identify and address barriers to optimal implementation. Utilizing models, such as the Ottawa Model for Research Use recommended by Santesso & Tugwell, throughout the KT process may prove useful to support effective implementation of KT strategies and positive outcomes.[115,127]

Limitations

Due to the rapid nature of this scoping review, it is possible we may have missed relevant strategies in the search. Another potential limitation is only identifying one piece of

grey literature in the scoping review, which may have been due to our search strategy and strict inclusion criteria. However, our comprehensive literature and grey literature searches identified a broad range of relevant strategies across countries and SRMNCAH priorities. The challenges of identifying relevant KT strategies and policies may also be due to how varied the terminology is around knowledge translation (e.g., knowledge transfer, knowledge exchange, knowledge mobilization) and how it is applied across different countries and health areas.[128] Additionally, while few KT strategies included in the review were implemented and evaluated in low-income countries, this may not reflect all of the KT strategies being implementing across these settings. Finally, findings from the review are up to date as of July 2020 and it is possible additional relevant strategies may have been published between running the search strategy and report writing. However, this review provides a comprehensive view of KT strategies which have been published over the past 20 years to address the health and wellbeing of women, children and adolescents.

CONCLUSION

Most KT strategies for SRMNCAH and wellbeing are being implemented in high-income countries and aimed at maternal, newborn and child health and wellbeing. We identified a critical gap in literature of KT approaches in low-income countries and humanitarian and fragile settings. Meaningful engagement of stakeholders in KT is key to enhancing people-centered and gender-responsive SRMNCAH policy, service delivery and financing. Effective KT approaches are required to support the implementation and impact of multi-sectoral policies and interventions. As SRMNCAH outcomes worsen as a consequence of the COVID-19 pandemic, it is critical to support effective SRMNCAH interventions and protect the progress made to date. Effective knowledge translation

For peer review only - http://bmjopen.bmj.com/site/about/guidelines.xhtml

approaches have a key role to play to ensure that strategies to mitigate the disruptions to SRMNCAH services are effective, feasible and acceptable, while addressing the equity gap and ensuring that vulnerable communities are not left behind.

ABBREVIATIONS:

BCW: Behaviour Change Wheel

KT: Knowledge Translation

PMNCH: Partnership for Maternal, Newborn & Child Health

PRESS: Peer Review of Electronic Search Strategy

PRISMA-ScR: Preferred Reporting Items for Systematic Reviews and Meta-Analyses - Extension for Scoping Reviews

SPOR: Strategy for Patient-Oriented Research

SRHR: Sexual and Reproductive Health and Rights

SRMNCAH: Sexual, Reproductive, Maternal, Newborn, Child, and Adolescent Health

WHO: World Health Organization

CONTRIBUTIONS: EVL, ZB, JAC, HW, RU, LB and LW developed the scoping review protocol. JAC, AJG, HDS and JK contributed to developing the initial draft of the manuscript. All authors provided feedback and revisions to the manuscript.

FUNDING: This work was funded by the SPOR Evidence Alliance in collaboration with the Partnership for Maternal, Newborn & Child Health (PMNCH) at the World Health Organization (Grant Number: Not Applicable)

COMPETING INTERESTS: The authors have no conflicts to declare

ETHICS STATEMENT: Not Applicable

DATA SHARING: Data sharing not applicable as no datasets generated and/or analysed for this study

For peer review only

REFERENCES

1. Victora, C. G. *et al.* Countdown to 2015: a decade of tracking progress for maternal, newborn, and child survival. *Lancet Lond. Engl.* **387**, 2049–2059 (2016).
2. Protect the progress: rise, refocus and recover. 2020 progress report on the Every Woman Every Child Global Strategy for Women's, Children's and Adolescents' Health (2016–2030). Geneva: World Health Organization and the United Nations Children's Fund (UNICEF); 2020.. https://data.unicef.org/resources/protect-the-progress-rise-refocus-recover-ewec-2020/.
3. Moynihan, R. *et al.* Pandemic impacts on healthcare utilisation: a systematic review. *medRxiv* 2020.10.26.20219352 (2020) doi:10.1101/2020.10.26.20219352.
4. Chmielewska, B. *et al.* Effects of the COVID-19 pandemic on maternal and perinatal outcomes: a systematic review and meta-analysis. *Lancet Glob. Health* **0**, (2021).
5. United Nations Population Fund (UNPFA). Impact of COVID-19 on Family Planning: What we know one year into the pandemic. /resources/impact-covid-19-family-planning-what-we-know-one-year-pandemic (2021).
6. WHO. *Pulse survey on continuity of essential health services during the COVID-19 pandemic: interim report, 27 August 2020*. https://www.who.int/publications-detail-redirect/WHO-2019-nCoV-EHS_continuity-survey-2020.1 (2020).
7. Countdown 2015 Maternal, Newborn & Child Survival – A Decade of Tracking Progress for Maternal, Newborn and Child Survival: The 2015 Report. http://countdown2030.org/2015/2015-final-report.
8. Organisation for Economic Co-operation and Development. The territorial impact of COVID-19: Managing the crisis across levels of government. *OECD* https://www.oecd.org/coronavirus/policy-responses/theterritorial-impact-of-covid-19-managing-the-crisis-across-levels-ofgovernment-d3e314e1/ (2020).

For peer review only

9. Jha, A., Kickbusch, I., Taylor, P., Abbasi, K., & SDGs Working Group. Accelerating achievement of the sustainable development goals. *BMJ* **352**, i409 (2016).

10. Canadian Institute for Health Research. About Knowledge Translation. https://cihr-irsc.gc.ca/e/29418.html#2 (2016).

11. Milat, A. J. & Li, B. Narrative review of frameworks for translating research evidence into policy and practice. *Public Health Res. Pract.* **27**, (2017).

12. Tabak, R. G., Khoong, E. C., Chambers, D. A. & Brownson, R. C. Bridging research and practice: models for dissemination and implementation research. *Am. J. Prev. Med.* **43**, 337–350 (2012).

13. Slaughter, S. E. *et al.* Classification schemes for knowledge translation interventions: a practical resource for researchers. *BMC Med. Res. Methodol.* **17**, 161 (2017).

14. Tricco, A. C. *et al.* PRISMA Extension for Scoping Reviews (PRISMA-ScR): Checklist and Explanation. *Ann. Intern. Med.* **169**, 467 (2018).

15. Peters, M. D. J. *et al.* Guidance for conducting systematic scoping reviews. *Int. J. Evid. Based Healthc.* **13**, 141–146 (2015).

16. McGowan, J. *et al.* PRESS Peer Review of Electronic Search Strategies: 2015 Guideline Statement. *J. Clin. Epidemiol.* **75**, 40–46 (2016).

17. Covidence. Covidence systematic review software, Veritas Health Innovation, Melbourne, Australia. Available at www.covidence.org.

18. The World Bank. World Bank Country and Lending Groups. https://datahelpdesk.worldbank.org/knowledgebase/articles/906519-world-bank-country-and-lending-groups.

19. The Partnership for Maternal, Newborn and Child Health (PMNCH). *PMNCH 2021-2025 Strategy*.

For peer review only

https://www.who.int/pmnch/knowledge/publications/pmnch_strategic_plan_2021_2025.pdf?ua=1 (2021).

20. Michie, S., Atkins, L. & West, R. *The Behaviour Change Wheel: A Guide to Designing Interventions*. (Silverback Publishing, 2014).
21. Michie, S., van Stralen, M. M. & West, R. The behaviour change wheel: A new method for characterising and designing behaviour change interventions. *Implement. Sci. IS* **6**, 42 (2011).
22. Page, M. J. *et al.* The PRISMA 2020 statement: an updated guideline for reporting systematic reviews. *BMJ* **372**, n71 (2021).
23. Abney-Roberts, S. E. A Successful Quality Improvement Project to Improve Infant Safe Sleep Practice. *J. Obstet. Gynecol. Neonatal Nurs.* **44**, S43 (2015).
24. Agapidaki, E. *et al.* A theory-based educational intervention to pediatricians in order to improve identification and referral of maternal depression: a quasi-experimental study. *Ann. Gen. Psychiatry* **12**, 37 (2013).
25. Ainsworth, R. M., Mog, C. & Summerlin-Long, S. A Comprehensive Newborn Falls Initiative One Year Later. *J. Obstet. Gynecol. Neonatal Nurs.* **43**, S66 (2014).
26. Al-Rafay, S. S. & Al-Sharkawy, S. S. Educational outcomes associated with providing a comprehensive guidelines program about nursing care of preterm neonates receiving total parenteral nutrition. *Clin. Nurs. Res.* **21**, 142–158 (2012).
27. Alkon, A. *et al.* Nutrition and physical activity randomized control trial in child care centers improves knowledge, policies, and children's body mass index. *BMC Public Health* **14**, 215 (2014).
28. Allen, M. & Schafer, D. J. Nurses Improving the Health of Mothers and Infants by Dancing the 10 Steps to Successful Breastfeeding. *J. Obstet. Gynecol. Neonatal Nurs.* **44**, S52–S52 (2015).

For peer review only

29. Anaby, D., Korner-Bitensky, N., Law, M. & Cormier, I. Focus on participation for children and youth with disabilities: Supporting therapy practice through a guided knowledge translation process. *Br. J. Occup. Ther.* **78**, 440–449 (2015).

30. Ansbro, É. M. *et al.* Introduction of Syphilis Point-of-Care Tests, from Pilot Study to National Programme Implementation in Zambia: A Qualitative Study of Healthcare Workers' Perspectives on Testing, Training and Quality Assurance. *PloS One* **10**, e0127728 (2015).

31. Baer, V. L. *et al.* Implementing a program to improve compliance with neonatal intensive care unit transfusion guidelines was accompanied by a reduction in transfusion rate: a pre-post analysis within a multihospital health care system. *Transfusion (Paris)* **51**, 264–269 (2011).

32. Brennan, M. M. *et al.* Paediatric resuscitation for nurses working in Ghana: an educational intervention. *Int. Nurs. Rev.* **60**, 136–143 (2013).

33. Cameron, D., Russell, D. J., Rivard, L., Darrah, J. & Palisano, R. Knowledge brokering in children's rehabilitation organizations: perspectives from administrators. *J. Contin. Educ. Health Prof.* **31**, 28–33 (2011).

34. Farner, R. *et al.* The nurse champion model for advancing newborn screening of critical congenital heart disease. *J. Obstet. Gynecol. Neonatal Nurs. JOGNN* **43**, 497–506 (2014).

35. Finch, M. *et al.* Impact of a population based intervention to increase the adoption of multiple physical activity practices in centre based childcare services: a quasi experimental, effectiveness study. *Int. J. Behav. Nutr. Phys. Act.* **9**, 101 (2012).

36. Goyet, S. *et al.* Knowledge translation: a case study on pneumonia research and clinical guidelines in a low- income country. *Implement. Sci.* **9**, 82 (2014).

For peer review only

37. Hulton, L. *et al.* Using evidence to drive action: a 'revolution in accountability' to implement quality care for better maternal and newborn health in Africa. *Int. J. Gynaecol. Obstet. Off. Organ Int. Fed. Gynaecol. Obstet.* **127**, 96–101 (2014).

38. Jones, C. O. H. *et al.* 'Even if you know everything you can forget': health worker perceptions of mobile phone text-messaging to improve malaria case-management in Kenya. *PloS One* **7**, e38636 (2012).

39. Kapungu, C. T. *et al.* A community-based continuum of care model for the prevention of postpartum hemorrhage in rural Ghana. *Int. J. Gynaecol. Obstet. Off. Organ Int. Fed. Gynaecol. Obstet.* **120**, 156–159 (2013).

40. Lang, J. M., Franks, R. P., Epstein, C., Stover, C. & Oliver, J. A. Statewide dissemination of an evidence-based practice using Breakthrough Series Collaboratives. *Child. Youth Serv. Rev.* **55**, 201–209 (2015).

41. Mello, M. J. *et al.* Translation of alcohol screening and brief intervention guidelines to pediatric trauma centers. *J. Trauma Acute Care Surg.* **75**, S301-307 (2013).

42. Mody, S. K., Ba-Thike, K. & Gaffield, M. E. The World Health Organization-United Nations Population Fund Strategic Partnership Programme's implementation of family planning guidelines and tools in Asia-Pacific countries. *J. Obstet. Gynaecol. Res.* **39**, 825–830 (2013).

43. Schreiber, J., Marchetti, G. F., Racicot, B. & Kaminski, E. The use of a knowledge translation program to increase use of standardized outcome measures in an outpatient pediatric physical therapy clinic: administrative case report. *Phys. Ther.* **95**, 613–629 (2015).

44. Schwartz, R. *et al.* Washington 'Steps' Up: A 10-Step Quality Improvement Initiative to Optimize Breastfeeding Support in Community Health Centers. *J. Hum. Lact. Off. J. Int. Lact. Consult. Assoc.* **31**, 651–659 (2015).

For peer review only

45. Segre, L. S., Brock, R. L., O'Hara, M. W., Gorman, L. L. & Engeldinger, J. Disseminating perinatal depression screening as a public health initiative: a train-the-trainer approach. *Matern. Child Health J.* **15**, 814–821 (2011).

46. Singh, K. *et al.* Impact evaluation of a quality improvement intervention on maternal and child health outcomes in Northern Ghana: early assessment of a national scale-up project. *Int. J. Qual. Health Care* **25**, 477–487 (2013).

47. Sobel, H. L. *et al.* Implementing a national policy for hepatitis B birth dose vaccination in Philippines: Lessons for improved delivery. *Vaccine* **29**, 941–945 (2011).

48. Sundaram, A., Juarez, F., Ahiadeke, C., Bankole, A. & Blades, N. The impact of Ghana's R3M programme on the provision of safe abortions and postabortion care. *Health Policy Plan.* **30**, 1017–1031 (2015).

49. Tarasoff, L. A., Epstein, R., Green, D. C., Anderson, S. & Ross, L. E. Using interactive theatre to help fertility providers better understand sexual and gender minority patients. *Med. Humanit.* **40**, 135–141 (2014).

50. Twum-Danso, N. A. *et al.* Using quality improvement methods to test and scale up a new national policy on early post-natal care in Ghana. *Health Policy Plan.* **29**, 622–632 (2014).

51. Wolfe, A. D. *et al.* Sharing life-altering information: development of pediatric hospital guidelines and team training. *J. Palliat. Med.* **17**, 1011–1018 (2014).

52. Okonofua, F., Lambo, E., Okeibunor, J. & Agholor, K. Advocacy for free maternal and child health care in Nigeria—Results and outcomes. *Health Policy* **99**, 131–138 (2011).

53. Carmona J.M.; Howe E.; Zapata R.; Stevens D.; Murphy J.K. The teen health improvement program: A system-wide initiative to improve the quality of care for adolescents served by the country's largest public hospital system. in (2015).

For peer review only

54. Alvarez, E. *et al.* Developing evidence briefs for policy: a qualitative case study comparing the process of using a guidance-contextualization workbook in Peru and Uganda. *Health Res. Policy Syst.* **17**, 89 (2019).

55. Barnard, J. G. *et al.* Facilitators and barriers to the use of standing orders for vaccination in obstetrics and gynecology settings. *Am. J. Obstet. Gynecol.* **216**, 69.e1-69.e7 (2017).

56. Becker-Haimes, E. M., Franklin, M., Bodie, J. & Beidas, R. S. Feasibility and Acceptability of a Toolkit to Facilitate Clinician Use of Exposure Therapy for Youth. *Evid.-Based Pract. Child Adolesc. Ment. Health* **2**, 165–178 (2017).

57. Boyko, J. A., Kothari, A. & Wathen, C. N. Moving knowledge about family violence into public health policy and practice: a mixed method study of a deliberative dialogue. *Health Res. Policy Syst.* **14**, 31 (2016).

58. Braddick, L. *et al.* A mixed-methods study of barriers and facilitators to the implementation of postpartum hemorrhage guidelines in Uganda. *Int. J. Gynaecol. Obstet. Off. Organ Int. Fed. Gynaecol. Obstet.* **132**, 89–93 (2016).

59. Burgoine, K. *et al.* Staged implementation of a two-tiered hospital-based neonatal care package in a resource-limited setting in Eastern Uganda. *BMJ Glob. Health* **3**, (2018).

60. Close, K., Karel, M. & White, M. A pilot program of knowledge translation and implementation for newborn resuscitation using US Peace Corps Volunteers in rural Madagascar. *Glob. Health* **12**, (2016).

61. Edwards, N. *et al.* The impact of leadership hubs on the uptake of evidence-informed nursing practices and workplace policies for HIV care: a quasi-experimental study in Jamaica, Kenya, Uganda and South Africa. *Implement. Sci. IS* **11**, 110 (2016).

62. Finch, M. *et al.* Challenges of Increasing Childcare Center Compliance With Nutrition Guidelines: A Randomized Controlled Trial of an Intervention Providing Training,

For peer review only

Written Menu Feedback, and Printed Resources. *Am. J. Health Promot. AJHP* **33**, 399–411 (2019).

63. George E.; Maloney F.; Kara N.; Bukoye B.; Benotti E.; Delaney M.M.; Nejad S.B.; Kumar N.D.; Semrau K. FACTORS DETERMINING THE UTILIZATION OF ANTENATAL CARE IN RURAL AND URBAN SETTINGS IN MOZAMBIQUE. *Int. J. Gynecol. Obstet.* **143**, 523 (2018).
64. Gera, R. *et al.* Implementation of "health systems approach" to improve vaccination at birth in institutional deliveries at public health facilities; experience from six states of India. *J. Fam. Med. Prim. Care* **8**, 1630–1636 (2019).
65. Gichane, M. W., Mutesa, M. & Chowa, G. Translating Evidence into Policy Change: Advocacy for Community-Based Distribution of Injectable Contraceptives in Zambia. *Glob. Soc. Welf.* **6**, 41–47 (2019).
66. Gilkey, M. B., Parks, M. J., Margolis, M. A., McRee, A.-L. & Terk, J. V. Implementing Evidence-Based Strategies to Improve HPV Vaccine Delivery. *Pediatrics* **144**, (2019).
67. Grady, A. *et al.* Effectiveness of a dissemination strategy on the uptake of an online menu planning program: A controlled trial. *Health Promot. J. Aust. Off. J. Aust. Assoc. Health Promot. Prof.* **30 Suppl 1**, 20–25 (2019).
68. Guillory, C. *et al.* Texas Pulse Oximetry Project: A Multicenter Educational and Quality Improvement Project for Implementation of Critical Congenital Heart Disease Screening Using Pulse Oximetry. *Am. J. Perinatol.* **34**, 856–860 (2017).
69. Hardee, K. *et al.* Improving Voluntary, Rights-Based Family Planning: Experience From Nigeria And Uganda. *Open Access J. Contracept.* **10**, 55–67 (2019).
70. Kraft, J. M. *et al.* Dissemination and use of WHO family planning guidance and tools: a qualitative assessment. *Health Res. Policy Syst.* **16**, 42 (2018).

For peer review only

71. Makkar, S. R., Howe, M., Williamson, A. & Gilham, F. Impact of tailored blogs and content on usage of Web CIPHER - an online platform to help policymakers better engage with evidence from research. *Health Res. Policy Syst.* **14**, 85 (2016).

72. Ongolo-Zogo, P., Lavis, J. N., Tomson, G. & Sewankambo, N. K. Assessing the influence of knowledge translation platforms on health system policy processes to achieve the health millennium development goals in Cameroon and Uganda: a comparative case study. *Health Policy Plan.* **33**, 539–554 (2018).

73. Segre, L. S., Trusty, S., Gullickson, R., Chuffo Davila, R. & O'Hara, M. W. Brokering the Evidence-Practice Gap: A Strategy for Moving Evidence Into Clinical Practice. *Psychiatr. Serv. Wash. DC* **69**, 852–854 (2018).

74. Seward, K. *et al.* Improving the implementation of nutrition guidelines in childcare centres improves child dietary intake: findings of a randomised trial of an implementation intervention. *Public Health Nutr.* **21**, 607–617 (2018).

75. Uneke, C. J., Sombie, I., Uro-Chukwu, H. C. & Johnson, E. Using equitable impact sensitive tool (EQUIST) to promote implementation of evidence informed policymaking to improve maternal and child health outcomes: a focus on six West African Countries. *Glob. Health* **14**, 104 (2018).

76. Uneke, C. J., Sombie, I., Uro-Chukwu, H. C., Johnson, E. & Okonofua, F. Using equitable impact sensitive tool (EQUIST) and knowledge translation to promote evidence to policy link in maternal and child health: report of first EQUIST training workshop in Nigeria. *Pan Afr. Med. J.* **28**, 37 (2017).

77. Vlad, I. *et al.* Improving quality for maternal care - a case study from Kerala, India. *F1000Research* **5**, (2016).

78. Wolfenden, L. *et al.* Multi-strategic intervention to enhance implementation of healthy canteen policy: a randomised controlled trial. *Implement. Sci. IS* **12**, (2017).

For peer review only

79. Yoong, S. L. *et al.* A theory-based evaluation of a dissemination intervention to improve childcare cooks' intentions to implement nutritional guidelines on their menus. *Implement. Sci.* **11**, 105 (2016).

80. Kingsnorth, S., Orava, T., Parker, K. & Milo-Manson, G. From knowledge translation theory to practice: developing an evidence to care hub in a pediatric rehabilitation setting. *Disabil. Rehabil.* **42**, 869–879 (2020).

81. Eriksson, L., Bergström, A., Hoa, D. T. P., Nga, N. T. & Eldh, A. C. Sustainability of knowledge implementation in a low- and middle- income context: Experiences from a facilitation project in Vietnam targeting maternal and neonatal health. *PLOS ONE* **12**, e0182626 (2017).

82. Simioni, A. T. *et al.* [Regionalization of perinatal health care in the province of Santa Fe, Argentina]. *Rev. Panam. Salud Publica Pan Am. J. Public Health* **41**, e38 (2017).

83. Cunningham, B. J. & Cardy, J. O. Using implementation science to engage stakeholders and improve outcome measurement in a preschool speech-language service system. *Speech Lang. Hear.* **23**, 17–24 (2020).

84. Agency for Healthcare Research and Quality. *AHRQ Safety Program for Perinatal Care: Experiences From the Frontline. Prepared under contract no. 2902010000241 (RTI International). AHRQ Publication No. 17-0003-23-EF. Rockville, MD: Agency for Healthcare Research and Quality. October 30, 2016. www.ahrq.gov/perinatalsafety.* (2016).

85. Akter, S. F. U., Heller, R. D., Smith, A. J. & Milly, A. F. Impact of a training intervention on use of antimicrobials in teaching hospitals. *J. Infect. Dev. Ctries.* **3**, 447–451 (2009).

86. Allen, C. W. & Jeffery, H. Implementation and evaluation of a neonatal educational program in rural Nepal. *J. Trop. Pediatr.* **52**, 218–222 (2006).

For peer review only

87. Berglund, A., Lefevre-Cholay, H., Bacci, A., Blyumina, A. & Lindmark, G. Successful implementation of evidence-based routines in Ukrainian maternities. *Acta Obstet. Gynecol. Scand.* **89**, 230–237 (2010).

88. Deorari, A. K., Paul, V. K., Singh, M., Vidyasagar, D., & Medical Colleges Network. Impact of education and training on neonatal resuscitation practices in 14 teaching hospitals in India. *Ann. Trop. Paediatr.* **21**, 29–33 (2001).

89. Ding, H. *et al.* Influencing the use of antibiotics in a Chinese pediatric intensive care unit. *Pharm. World Sci. PWS* **30**, 787–793 (2008).

90. Jeffery, H. E. *et al.* The impact of evidence-based education on a perinatal capacity-building initiative in Macedonia. *Med. Educ.* **38**, 435–447 (2004).

91. Uskun, E., Uskun, S. B., Uysalgenc, M. & Yagiz, M. Effectiveness of a training intervention on immunization to increase knowledge of primary healthcare workers and vaccination coverage rates. *Public Health* **122**, 949–958 (2008).

92. Warren, C., Mwangi, A., Oweya, E., Kamunya, R. & Koskei, N. Safeguarding maternal and newborn health: improving the quality of postnatal care in Kenya. *Int. J. Qual. Health Care* **22**, 24–30 (2010).

93. Agency for Healthcare Research and Quality. *Care Transitions from Hospital to Home: IDEAL Discharge Planning Implementation Handbook. Guide to Patient and Family Engagement.*

94. Alton, M., Frush, K., Brandon, D. & Mericle, J. Development and implementation of a pediatric patient safety program. *Adv. Neonatal Care Off. J. Natl. Assoc. Neonatal Nurses* **6**, 104–111 (2006).

95. Brown, J. J., Wacogne, I., Fleckney, S., Jones, L. & Bhrolchain, C. N. Achieving early surgery for undescended testes: quality improvement through a multifaceted approach to guideline implementation. *Child Care Health Dev.* **31**, 119–119 (2005).

For peer review only - http://bmjopen.bmj.com/site/about/guidelines.xhtml

96. Carlo, W. A. *et al.* Educational impact of the neonatal resuscitation program in low-risk delivery centers in a developing country. *J. Pediatr.* **154**, 504-508.e5 (2009).

97. Crone, M. R. *et al.* Sustainability of the Prevention of Passive Infant Smoking Within Well-Baby Clinics. *Health Educ. Behav.* **33**, 178–196 (2006).

98. Margolis, P. A. *et al.* From concept to application: the impact of a community-wide intervention to improve the delivery of preventive services to children. *Pediatrics* **108**, E42 (2001).

99. Pariyo, G. W., Gouws, E., Bryce, J., Burnham, G., & Uganda IMCI Impact Study Team. Improving facility-based care for sick children in Uganda: training is not enough. *Health Policy Plan.* **20 Suppl 1**, i58–i68 (2005).

100. Schreiber, J., Stern, P., Marchetti, G. & Provident, I. Strategies to promote evidence-based practice in pediatric physical therapy: a formative evaluation pilot project. *Phys. Ther.* **89**, 918–933 (2009).

101. R Simmons, Fajans, P. & Ghiron, L. Chapter 2: Strategic choices in scaling up: introducing injectable contraception and improving quality of care in Viet Nam. in *Scaling up health service delivery: from pilot innovations to policies and programmes* (World health Organization, 2007).

102. Goering, M. & Wilson, W. Implementing preterm labor guidelines: a collaborative care improvement process. *J. Perinat. Neonatal Nurs.* **16**, 47–57 (2002).

103. Davies, B. *et al.* Fetal health surveillance: a community-wide approach versus a tailored intervention for the implementation of clinical practice guidelines. *CMAJ Can. Med. Assoc. J. J. Assoc. Medicale Can.* **167**, 469–474 (2002).

104. English, K. C., Merzel, C. & Moon-Howard, J. Translating public health knowledge into practice: development of a lay health advisor perinatal tobacco cessation program. *J. Public Health Manag. Pract. JPHMP* **16**, E9–E19 (2010).

For peer review only

105. Russell, D. J. *et al.* Using knowledge brokers to facilitate the uptake of pediatric measurement tools into clinical practice: a before-after intervention study. *Implement. Sci. IS* **5**, 92 (2010).

106. R Simmons, Fajans, P. & Ghiron, L. Chapter 7 Scaling up family planning service innovations in Brazil: the influence of politics and decentralization. in *Scaling up health service delivery: from pilot innovations to policies and programmes* (World health Organization, 2007).

107. Di Noia, J., Schwinn, T. M., Dastur, Z. A. & Schinke, S. P. The relative efficacy of pamphlets, CD-ROM, and the Internet for disseminating adolescent drug abuse prevention programs: an exploratory study. *Prev. Med.* **37**, 646–653 (2003).

108. Dobbins, M. *et al.* A randomized controlled trial evaluating the impact of knowledge translation and exchange strategies. *Implement. Sci.* **4**, 61 (2009).

109. Melnyk, B. M. *et al.* Translating the evidence-based NICU COPE program for parents of premature infants into clinical practice: impact on nurses' evidence-based practice and lessons learned. *J. Perinat. Neonatal Nurs.* **24**, 74–80 (2010).

110. Rowe, A. K. *et al.* A multifaceted intervention to improve health worker adherence to integrated management of childhood illness guidelines in Benin. *Am. J. Public Health* **99**, 837–846 (2009).

111. Senarath, U., Fernando, D. N. & Rodrigo, I. Effect of training for care providers on practice of essential newborn care in hospitals in Sri Lanka. *J. Obstet. Gynecol. Neonatal Nurs. JOGNN* **36**, 531–541 (2007).

112. Snelgrove-Clarke, E. E. The effects of action learning on nurses' use of a fetal health surveillance guideline with low-risk labouring women. https://escholarship.mcgill.ca/concern/theses/dz010q44w.

For peer review only

113. Daniels, K., Lewin, S., & Practice Policy Group. Translating research into maternal health care policy: a qualitative case study of the use of evidence in policies for the treatment of eclampsia and pre-eclampsia in South Africa. *Health Res. Policy Syst.* **6**, 12 (2008).

114. Organisation for Economic Co-operation and Development. States of Fragility 2020. https://www.oecd-ilibrary.org/development/states-of-fragility-2020_ba7c22e7-en (2020).

115. Santesso, N. & Tugwell, P. Knowledge translation in developing countries. *J. Contin. Educ. Health Prof.* **26**, 87–96 (2006).

116. Nabyonga Orem, J. *et al.* Perspectives on the role of stakeholders in knowledge translation in health policy development in Uganda. *BMC Health Serv. Res.* **13**, 324 (2013).

117. World Health Organization. Millennium Development Goals (MDGs). https://www.who.int/news-room/fact-sheets/detail/millennium-development-goals-(mdgs) (2018).

118. Fenny, A. P., Crentsil, A. O. & Ackah, C. The health MDGs in Ghana: lessons and implications for the implementation of the sustainable development goals. *J. Public Health* **26**, 225–234 (2018).

119. Bendavid, E. *et al.* The effects of armed conflict on the health of women and children. *The Lancet* **397**, 522–532 (2021).

120. Stark, L., Seff, I. & Reis, C. Gender-based violence against adolescent girls in humanitarian settings: a review of the evidence. *Lancet Child Adolesc. Health* **5**, 210–222 (2021).

121. Oduro-Mensah, E. *et al.* Care Decision Making of Frontline Providers of Maternal and Newborn Health Services in the Greater Accra Region of Ghana. *PLOS ONE* **8**, e55610 (2013).

For peer review only

For peer review only - http://bmjopen.bmj.com/site/about/guidelines.xhtml

122. UNICEF. Adolescent health and well-being. https://www.unicef.org/health/adolescent-health-and-well-being (2021).

123. Partnership for MAternal, Newborn and Child health. Adolescent health and well-being. *WHO* http://www.who.int/pmnch/media/news/2019/Adolescent_health_and_well-being_webinars/en/ (2020).

124. World Health Organization. WHO | Aligning public financial management and health financing. *WHO* http://www.who.int/health_financing/documents/aligning-pfm-health-financing/en/ (2017).

125. World Health Organization Regional Office for South-East Asia. Continuing essential Sexual Reproductive, Maternal, Neonatal, Child and Adolescent Health services during COVID-19 pandemic. (2020).

126. Dal Mas, F., Biancuzzi, H., Massaro, M. & Miceli, L. Adopting a knowledge translation approach in healthcare co-production. A case study. *Manag. Decis.* **58**, 1841–1862 (2020).

127. Graham, I. D. & Logan, J. Innovations in knowledge transfer and continuity of care. *Can. J. Nurs. Res. Rev. Can. Rech. En Sci. Infirm.* **36**, 89–103 (2004).

128. Graham, I. D. *et al.* Lost in knowledge translation: Time for a map? *J. Contin. Educ. Health Prof.* **26**, 13–24 (2006).

129. Michie, S., Atkins, L. & West, R. *The Behaviour Change Wheel (Behavior Change Wheel) - A Guide To Designing Interventions*. (Silverback Publishing, 2014).

For peer review only - http://bmjopen.bmj.com/site/about/guidelines.xhtml

FIGURE 1. PRISMA-ScR Flow Diagram

For peer review only

Publication Date	Country by Income Level	Study Design	SRMNCAH Priorities	Examples of SRMNCAH Health Topics	Settings	PMNCH Function of Interest
2000-2005:<br>**n=8 (9%)**<br>2006-2010:<br>**n=20 (22 %)**<br>2011-2015:<br>**n=31 (34 %)**<br>2016-2020:<br>**n=31 (34 %)**	**Low:**<br>n= 4 (4%)<br>**Low& Middle:**<br>n= 6 (7%)<br>**Lower middle:** n= 26 (81%)<br>**Upper middle:**<br>n=6 (19%)<br><br>**Middle & High:**<br>n= 1 (1%)<br>**High:**<br>n= 47 (52%)	**Experimental:**<br>n=14 (16 %)<br>**Quasi-experimental:**<br>n= 29 (32%)<br>**Observational:**<br>n= 20 (22%)<br>**Qualitative:**<br>n= 10 (11%)<br>**Mixed methods:**<br>n= 15 (17%)<br>**Editorials:**<br>n= 2 (2%)	**Adolescent:**<br>n=4 (4%)<br>**Child:**<br>n=28 (31%)<br>**Maternal:**<br>n=14 (16%)<br>**Newborn or stillbirths:**<br>n=20 (22%)<br>**Maternal & newborn:**<br>n=8 (9%)<br>**Maternal & child:**<br>n=2 (2%)<br>**Maternal & child & newborn:**<br>n=2 (2%)<br>**SRHR:**<br>n=12 (13%)	**Adolescent:**<br>Substance use[107]<br>**Child:**<br>Child nutrition[27,35,38,67,74,78,79]<br>**Maternal:**<br>Postpartum depression,[45] eclampsia and pre-eclampsia[113]<br>**Newborn or stillbirths:**<br>Newborn sleep,[23] newborn vaccination[97]<br>**SRHR:**<br>Family planning[42,49,65,69,70,101]	**Hospitals:**<br>n= 35 (39%)<br>**Community:**<br>n= 23 (25%)<br>**Childcare centres or schools:**<br>n=10 (11%)<br>**Primary Care:**<br>n=7 (8%)<br>**Government departments:**<br>n=3 (3%)	**Financing:**<br>n=0<br>**Policymaking:**<br>n= 17 (19%)<br>**Service delivery:**<br>n= 73 (81%)

Table 1. Overview of characteristics of included studies (n=90)
PMNCH: the Partnership for Maternal, Newborn & Child Health
SRMNCAH: sexual, reproductive, maternal, newborn, child and adolescent health
SRHR: sexual and reproductive health and rights

For peer review only

BCW Intervention Function Mapping				
**Intervention Function and Definition** [129]	**Function Identified Across KT Strategies**	**Intervention Function Stratified by Country Income Level***	**Intervention Function Stratified by SRMNCAH priority****	**Exemplar Quote**
**Education** (Increasing knowledge or understanding)	n=73 (81%)	Low: n= 8 (80%)<br>Middle: n= 30 (77%)<br>High: n= 45 (88%)	Adolescent: n=3 (75%)<br>Child: n=29 (91%)<br>Maternal: n=21 (78%)<br>Newborn or stillbirths: n=23 (77%)<br>SRHR: n= 9 (75%)	*"To introduce the postnatal care package, a 3 day training took place for staff and their supervisors from the maternal and child health clinics and maternity units from four health facilities in one district."* [92]
**Training** (Imparting skills)	n= 46 (51%)	Low: n= 6 (60%)<br>Middle: n= 19 (41%)<br>High: n= 26 (52%)	Adolescent: n= 2 (50%)<br>Child: n= 14 (44%)<br>Maternal: n= 12 (46%)<br>Newborn or stillbirths: n= 16 (53%)<br>SRHR: n= 7 (58%)	*"Canteen managers, canteen staff and parent representatives were invited to attend a training workshop with the aim of providing education and skill development in the policy, nutrition and food label reading, canteen stock and financial management, pricing and promotion, and change management."* [78]
**Environmental Restructuring** (Changing the physical or social context)	n= 45 (50%)	Low: n= 4 (40%)<br>Middle: n= 16 (41%)<br>High: n= 30 (58%)	Adolescent: n= 1 (25%)<br>Child: n= 22 (69%)<br>Maternal: n= 10 (38%)	*"Dedicated neonatal unit with reliable water supply, hand-washing facilities, infection control policies and stable power source; Investment in appropriate technology*

For peer review only - http://bmjopen.bmj.com/site/about/guidelines.xhtml

			Newborn or stillbirths: n= 14 (47%)<br>SRHR: n= 6 (50%)	*for neonatal care; Dedicated neonatal staff including a paediatrician and neonatal nurse."* [59]
**Enablement** (Increasing means/reducing barriers)	n=22 (24%)	Low: n= 5 (50%)<br>Middle: n= 6 (15%)<br>High: n= 14 (29%)	Adolescent: n= 1 (25%)<br>Child: n= 8 (25%)<br>Maternal: n= 6 (23%)<br>Newborn or stillbirths: n= 4 (13%)<br>SRHR: n= 2 (13%)	*"Because practices and agencies face common barriers to improved care delivery, we sought to increase communication among them about how to overcome specific problems in care delivery."* [98]
**Persuasion** (Using communication to induce positive or negative feelings or stimulate action)	n= 11 (12%)	Low: n= 1 (10%)<br>Middle: n= 2 (5%)<br>High: n= 10 (21%)	Adolescent: n= 0<br>Child: n= 8 (25%)<br>Maternal: n=3 (12%)<br>Newborn or stillbirths: n= 1 (3%)<br>SRHR: n=1 (8%)	*"Posters and information leaflets for maternal depression which was placed in pediatric waiting room in order to motivate and facilitate mothers to discuss about depression."*[24]
**Modelling** (Providing an example for people to aspire to or imitate)	n= 7 (8%)	Low: n= 0<br>Middle: n= 3 (8%)<br>High: n= 4 (8%)	Adolescent: n= 0<br>Child: n= 2 (6%)<br>Maternal: n= 2 (8%)<br>Newborn or stillbirths: n= 2 (7%)<br>SRHR: n= 1 (8%)	*"Supervisors were prompted to endorse use of the program and general healthy eating within the centre to service educators and parents."*[67]
**Incentivisation** (Creating expectation of reward)	n= 5 (6%)	Low: n= 0<br>Middle: n= 1 (3%)<br>High: n= 4 (8%)	Adolescent: n= 0<br>Child: n= 4 (13%)<br>Maternal: n= 0	*"Participants received 1 hour of continuing medical education credit for their participation, and*

For peer review only

			Newborn or stillbirths: n= 0<br>SRHR: n= 1 (8%)	*physicians were awarded Maintenance of Certification Part 4 points for completion of the program."*[66]
**Coercion** (Creating expectation of punishment or cost)	n= 2 (2%)	Low: n= 1 (10%)<br>Middle: n= 1 (3%)<br>High: n=0	Adolescent: n= 0<br>Child: n= 0<br>Maternal: n= 1 (4%)<br>Newborn or stillbirths: n= 2 (7%)<br>SRHR: n=0	*"The program also included monthly compliance reporting of each NICU in the health care system to the administrative data center."* [31]
**Restriction** (Using rules to reduce the opportunity to engage in the target behaviour)	n= 1 (1%)	Low: n=0<br>Middle: n= 1 (3%)<br>High: n=0	Adolescent: n= 0<br>Child: n=0<br>Maternal: n= 0<br>Newborn or stillbirths: n=1 (3%)<br>SRHR: n= 0	*"The program involved ordering all blood products with electronic order entry. If a transfusion order did not comply with the Intermountain Healthcare transfusion guidelines, the ordering clinician could check an "other" category. If the "other" field was selected, a prompt was given to explain why the transfusion was being ordered outside of guidelines."* [31]
**BCW Policy Category Mapping**				
**Policy Category and Definition** [129]	**Policy Category Identified Across KT Strategies**	**Policy Category Stratified Country Income Level***	**Policy Category Stratified by SRMNCAH priority****	**Exemplar Quote**

For peer review only

**Guidelines** (Creating documents that recommend or mandate practice. This includes all changes to service provision)	n= 25 (28%)	Low: n= 5 (50%)<br>Middle: n= 12 (31%)<br>High: n= 12 (25%)	Adolescent: n= 1 (25%)<br>Child: n=6 (19%)<br>Maternal: n= 9 (35%)<br>Newborn or stillbirths: n=8 (27%)<br>SRHR: n= 5 (42%)	*"The researchers developed and implemented a comprehensive guidelines program about nursing care of total parenteral nutrition for preterm neonates."*[26]
**Service Provision** (Delivering a service)	n= 18 (20%)	Low: n= 0<br>Middle: n= 2 (5%)<br>High: n= 15 (31%)	Adolescent: n= 1 (25%)<br>Child: n=6 (19%)<br>Maternal: n= 4 (15%)<br>Newborn or stillbirths: n=7 (23%)<br>SRHR: n= 3 (25%)	*"Intervention services were provided with strategies to support adoption of improved childhood nutrition program."*[67]
**Communication /Marketing** (Using print, electronic, telephonic or broadcast media)	n= 12 (13%)	Low: n= 2 (20%)<br>Middle: n=4 (10%)<br>High: n= 8 (17%)	Adolescent: n= 0<br>Child: n=5 (16%)<br>Maternal: n= 3 (12%)<br>Newborn or stillbirths: n= (13%)<br>SRHR: n= 3 (25%)	*"This presentation was beamed on national television. The report was then presented to a meeting of the National Economic Advisory Group, chaired by the President, and coordinated by the Governor of the Central Bank of Nigeria, and also beamed on national television."* [52]
**Environmental/ Social Planning** (Designing and/or controlling the physical or social environment)	n= 11 (12%)	Low: n= 1 (10%)<br>Middle: n= 4 (10%)<br>High: n= 6 (12%)	Adolescent: n= 0<br>Child: n=4 (13%)<br>Maternal: n= 5 (19%)<br>Newborn or stillbirths: n=5 (17%)<br>SRHR: n=0	*"The safety program's organizational structure is designed to meet operational objectives providing: Resources and support for departmental, inpatient, and outpatient unit level safety teams."* [94]
**Regulation** (Establishing rules or principles of	n= 7 (8%)	Low: n= 1 (10%)<br>Middle: n= 2 (5%)<br>High: n= 5 (10%)	Adolescent: n= 0<br>Child: n=3 (9%)	*"Two faculty members from each hospital undertook a neonatal resuscitation*

For peer review only

behaviour or practice)			Maternal: n= 1 (3%)<br>Newborn or stillbirths: n=4 (13%)<br>SRHR: n=0	*certification course as per guidelines set out by the AAP/AHA."* [88]
**Legislation** (Making or changing laws)	n= 3 (3%)	Low: n= 0<br>Middle: n= 3(8%)<br>High: n= 0	Adolescent: n= 0<br>Child: n=0<br>Maternal: n= 1 (4%)<br>Newborn or stillbirths: n=0<br>Sexual or reproductive health and rights: n=2 (17%)	*"The Zambian government authorized the policy change in 2016."*[65]
**Fiscal** (Using the tax system to reduce or increase the financial cost)	n= 0	N/A	N/A	N/A

Table 2. Summary of BCW intervention functions and policy categories identified in KT strategies by country income level and SRMNCAH priority
* please note country income levels include seven multi-country studies (n=97)
** please note priorities include 12 multi-priority studies (n=104)
BCW: Behaviour Change Wheel
KT: knowledge translation
SRHR: sexual and reproductive health and rights
SRMNCAH: sexual, reproductive, maternal, newborn, child and adolescent health

For peer review only

Type of Stakeholders involved in KT Strategy	Stakeholders by Country Income Level*	Stakeholders by SRMNCAH Priority**
**Policymakers and Government**	Low: n= 5 (50%)<br>Middle: n= 24 (62%)<br>High: n= 4 (8%)	Adolescent: n= 1 (25%)<br>Child: n= 4 (13%)<br>Maternal: n= 9 (35%)<br>Newborn or stillbirths: n= 3 (10%)<br>SRHR: n= 7 (63%)
**Healthcare Providers and Administrators**	Low: n= 5 (50%)<br>Middle: n= 17 (44%)<br>High: n= 23 (48%)	Adolescent: n= 2 (50%)<br>Child: n= 10 (31%)<br>Maternal: n= 11 (42%)<br>Newborn or stillbirths: n= 9 (30%)<br>SRHR: n= 3 (25%)
**Civil Society Organizations**	Low: n= 6 (60%)<br>Middle: n= 19 (48%)<br>High: n= 13 (27%)	Adolescent: n= 1 (25%)<br>Child: n= 4 (13%)<br>Maternal: n= 3 (13%)<br>Newborn or stillbirths: n= 2 (7%)<br>SRHR: n= 7 (63%)
**Public**	Low: n= 2 (20%)<br>Middle: n= 1 (3%)<br>High: n= 10 (21%)	Adolescent: n= 2 (50%)<br>Child: n= 3 (9%)<br>Maternal: n= 1 (4%)<br>Newborn or stillbirths: n= 2 (7%)<br>SRHR: n= 2 (17%)
**Research Community**	Low: n= 3 (30%)<br>Middle: n= 10 (26%)<br>High: n= 17 (35%)	Adolescent: n= 1 (25%)<br>Child: n= 9 (28%)<br>Maternal: n= 10 (38%)<br>Newborn or stillbirths: n= 2 (7%)<br>SRHR: n= 3 (25%)

Table 3. Summary of stakeholder engagement by country income level and SRMNCAH priority

* please note country income levels include seven multi-country studies (n=97)

** please note priorities include 12 multi-priority studies (n=104)

KT: knowledge translation

SRHR: sexual and reproductive health and rights

SRMNCAH: sexual, reproductive, maternal, newborn, child and adolescent health

For peer review only

Barriers Identified Across Studies (n=39)			
	**Stratified Country Income Level***	**Stratified by SRMNCAH Priority****	**Exemplar Quote:**
**Limited resources (n=22)**	Low: n= 4 (40%)<br>Middle: n= 9 (23%)<br>High: n= 9 (19%)	Adolescent: n=2 (50%)<br>Child: n= 6 (19%)<br>Maternal: n= 6 (23%)<br>Newborn or stillbirths: n= 2 (7%)<br>SRHR: n= 6 (50%)	*"Funding and resource constraints are significant... Participants in Senegal noted that they do not always have sufficient materials to distribute to providers during training sessions."*[70]<br>*"Lack of funding and other necessary resources and the absence of concrete evidence of the impact of the Knowledge Brokers were identified as barriers to implementing knowledge brokering."*[33]
**Time constraints (n=12)**	Low: n= 0<br>Middle: n=2 (5%)<br>High: n=10 (21%)	Adolescent: n= 1 (25%)<br>Child: n= 5 (16%)<br>Maternal: n= 2 (8%)<br>Newborn or stillbirths: n= 2 (7%)<br>SRHR: n= 2 (13%)	*"Delays in implementing parts of the change management plan were as a result of time constraints."*[95]
**Negative attitudes (n=10)**	Low: n= 3 (30%)<br>Middle: n=3 (8%)<br>High: n=5 (10%)	Adolescent: n= 1 (25%)<br>Child: n= 2 (6%)<br>Maternal: n=3 (12%)<br>Newborn or stillbirths: n= 1 (3%)<br>SRHR: n= 3 (25%)	*"4 out of 10 Peace Corps. Volunteers reported some resistance to the teaching by older midwives in their health centres."* [60]
**Lack of knowledge (n=9)**	Low: n= 1 (10%)<br>Middle: n=3 (8%)<br>High: n=5 (10%)	Adolescent: n=1 (25%)<br>Child: n= 2 (6%)<br>Maternal: n=4 (15%)<br>Newborn or stillbirths: n= 3 (10%)<br>SRHR: n=1 (8%)	*"Limited availability of clinicians with expertise (limited number of skilled people dealing with too many issues in parallel)."*[36]
**Lack of training (n=7)**	Low: n= 0<br>Middle: n=0<br>High: n=7 (15%)	Adolescent: n= 1 (25%)<br>Child: n= 4 (13%)<br>Maternal: n= 2 (8%)<br>Newborn or stillbirths: n= 0	*"These include food service staff challenges with modifying recipes, inadequate access to updated menu planning resources, lack of training opportunities, and incorrect*

For peer review only - http://bmjopen.bmj.com/site/about/guidelines.xhtml

		SRHR: n=0	*assumptions by food service staff that menus and recipes adhere to nutrition guidelines."*[62]
**Poor engagement with stakeholders (n=7)**	Low: n= 0<br>Middle: n=4<br>High: n=3	Adolescent: n= 1 (25%)<br>Child: n= 3 (9%)<br>Maternal: n= 3 (12%)<br>Newborn or stillbirths: n= 1 (3%)<br>SRHR: n=1 (8%)	*"The project was criticised for lacking the involvement of certain stakeholders, for example, those at district level (district hospitals, district centres and district People's committees) and for providing opportunities for these parties to interact with project staff."*[81]<br>*"Engagement: the composition of the teams was highlighted as being a barrier. For example, more village health workers should have been included in the groups, and it would also have been appropriate to include other village representatives."*[81]
**Enablers Identified Across Studies (n=36)**			
	**Stratified Country Income Level***	**Stratified by SRMNCAH Priority****	**Exemplar Quote:**
**Supportive stakeholder involvement (n=24)**	Low: n= 4 (40%)<br>Middle: n= 10 (26%)<br>High: n= 10 (21%)	Adolescent: n= 2 (50%)<br>Child: n= 3 (9%)<br>Maternal: n=7 (27%)<br>Newborn or stillbirths: n= 4 (13%)<br>SRHR: n=8 (67%)	*"Engaging government officials throughout the research and advocacy processes was critical to policy advocacy efforts."*[65]<br>*"Collaboration with physician and nursing providers also was critical to the success of the initiative."*[102]
**Access to resources (n=8)**	Low: n= 2 (20%)<br>Middle: n=2 (5%)<br>High: n=4 (8%)	Adolescent: n= 0<br>Child: n= 1 (3%)<br>Maternal: n=3 (12%)<br>Newborn or stillbirths: n= 3 (10%)<br>SRHR: n= 2 (17%)	*"All clinicians reported that READY was easy to use and that they benefited from the tangible nature of the toolkit and the challenge cards. Clinicians specifically noted that having a physical resource greatly facilitated their use of READY."*[56]
**Access to knowledge (n=8)**	Low: n= 4 (40%)<br>Middle: n=4 (10%)	Adolescent: n= 0<br>Child: n= 2 (6%)	*" KT intervention provided relevant, accurate, robust,*

For peer review only

	High: n=0	Maternal: n= 3 (12%)<br>Newborn or stillbirths: n= 2 (7%)<br>SRHR: n= 3 (25%)	*comprehensive and accessible information to policymakers; Being members of the KT intervention group, the experts had privileged access to research findings before the final results were available."*[36]
**Positive attitudes, empowerment (n=7)**	Low: n= 2 (20%)<br>Middle: n=3 (8%)<br>High: n=2 (4%)	Adolescent: n= 0<br>Child: n= 2 (6%)<br>Maternal: n=4 (15%)<br>Newborn or stillbirths: n= 1 (3%)<br>SRHR: n=0	*"Empowering clinical staff to provide vaccinations before patients saw providers and increases in staff autonomy."*[55]
**Skills and training (n=6)**	Low: n= 0<br>Middle: n=0<br>High: n=6 (13%)	Adolescent: n=0<br>Child: n= 3 (9%)<br>Maternal: n=2 (8%)<br>Newborn or stillbirths: n= 0<br>SRHR: n=1 (8%)	*"Staff training was cited as another important facilitator because it provided the entire staff with a foundation of common knowledge. Following training by her practice to deliver Tdap, influenza, and HPV by standing order, one Medical Assistant told us she became quite comfortable with having immunization eligibility discussions with her patients."* [55]

Table 4. Summary of barriers and enablers identified across strategies by country income level and SRMNCAH priority

* please note country income levels include seven multi-country studies (n=97)

** please note priorities include 12 multi-priority studies (n=104)

SRHR: sexual and reproductive health and rights

SRMNCAH: sexual, reproductive, maternal, newborn, child and adolescent health

For peer review only

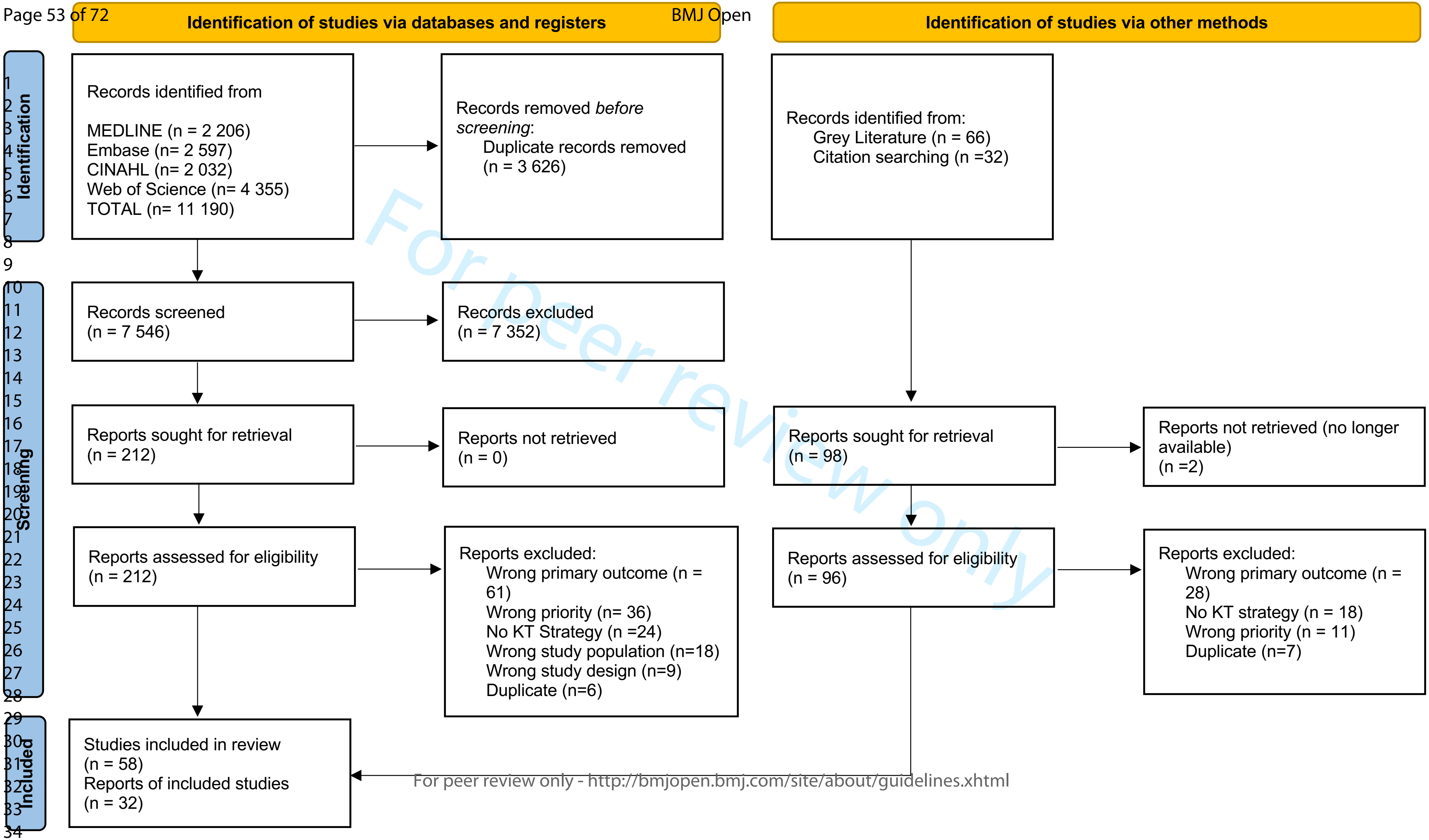

For peer review only

Supplementary File 1- Search Strategy

1 exp Diffusion of Innovation/
2 Information Dissemination/
3 Translational Medical Research/
4 ((disseminat* or implement* or share? or sharing or translat*) adj2 (activities or activity or approach? or model? or plan? or planning or program* or strategies or strategy or advanc* or better* or boost* or enhanc* or forward* or further* or improv* or increas* or bolster* or sustain* or uphold*)).ti,ab,kw,kf.
5 (evidence* adj2 (adapt* or adopt* or diffus* or disseminat* or exchang* or implement* or mobili* or share? or sharing or transfer* or translat* or utilis* or utiliz* or uptake)).ti,ab,kw,kf.
6 (information adj2 (adapt* or adopt* or diffus* or disseminat* or exchang* or mobili* or propagat* or share? or sharing or spread* or transfer* or translat*)).ti,ab,kw,kf.
7 (innovation? adj2 (adapt* or adopt* or diffus* or disseminat* or exchang* or implement* or mobili* or propagat* or share? or sharing or spread* or transfer* or translat* or transmi* or uptake)).ti,ab,kw,kf.
8 (knowledge adj2 (adapt* or adopt* or diffus* or disseminat* or exchang* or implement* or mobili* or propagat* or share? or sharing or spread* or transfer* or translat* or transmi* or uptake)).ti,ab,kw,kf.
9 (research adj2 (adapt* or adopt* or diffus* or disseminat* or exchang* or implement* or mobili* or propagat* or share? or sharing or spread* or transfer* or translat* or transmi* or utilis* or utiliz* or uptake)).ti,ab,kw,kf.
10 or/1-9 [KT]
11 Cartoons as Topic/
12 exp Mass Media/
13 Pamphlets/
14 Newspapers as Topic/
15 Webcasts as Topic/
16 Guidelines as Topic/
17 ((knowledge or evidence) adj (product? or summary or summaries or brief* or report? or portal? or repositor* or database? or center? or centre?)).ti,ab,kw,kf.
18 (advocacy brief* or strategy brief*).ti,ab,kw,kf.
19 (position statement? or consensus statement? or policy statement?).ti,ab,kw,kf.
20 (booklet? or brochure? or cartoon* or comic? or flyer? or infograph* or pamphlet? or poster?).ti,ab,kw,kf.
21 (media adj2 (communicat* or diffus* or disseminat* or exchang* or mobili* or populari* or propagat* or publici* or share? or sharing or spread*)).ti,ab,kw,kf.
22 (briefing? or news or newspaper? or press release?).ti,ab,kw,kf.
23 (thematic report? or accountability report? or evaluation report?).ti,ab,kw,kf.
24 (strategic plan? or annual report? or progress report?).ti,ab,kw,kf.
25 (online communication? or web communication? or video? or webcast* or podcast*).ti,ab,kw,kf.
26 (toolkit? or tool or tools or roadmap? or road map?).ti,ab,kw,kf.
27 (guideline? or standards).ti,ab,kw,kf.

For peer review only

28 or/11-27 [products]
29 10 and 28 [KT products]
30 ((evidence or information or innovation? or knowledge or research) adj2 (adapt* or adopt* or diffus* or disseminat* or exchang* or implement* or mobili* or propagat* or share? or sharing or spread* or transfer* or translat* or transmi* or uptake) adj2 (activities or activity or approach? or initiative? or plan? or policies or policy or procedure? or program* or scheme? or strategies or strategy)).ti,ab,kw,kf.
31 or/1-3
32 (activities or activity or approach? or initiative? or plan? or policies or policy or procedure? or program* or scheme? or strategies or strategy).ti.
33 31 and 32 [KT activities]
34 30 or 33 [KT activities or strategies]
35 29 or 34 [KT products, activities, or strategies (INTERVENTION)]
36 exp Administrative Personnel/
37 exp Policy Making/
38 (administrator? or decision maker? or policy maker? or policymaker?).ti,ab.
39 (knowledge user? or stakeholder?).ti,ab.
40 (advisory or board or committee? or task force?).ti,ab.
41 (((policy or policies) adj making) or policymaking or ((policy or policies) adj2 develop*) or ((policy or policies) adj2 analys?s) or (health adj (policy or policies))).ti,ab.
42 (legislator? or legislation?).ti,ab.
43 (health system? or health care system? or healthcare system? or system? level or system? manage*).ti,ab.
44 (civil societ* or third sector or non government* organi?ation? or nongovernment* organi?ation? or ngo or ngos).ti,ab.
45 (donor? or funder? or funding agen* or grant* agen*).ti,ab.
46 or/36-45 [POPULATION]
47 Reproductive Health/ or Reproductive Health Services/ or Family Planning Services/ or Sexual Health/ or Circumcision, Female/ or Menstruation/ or exp Puberty/ or exp Contraception/ or exp Sexually Transmitted Diseases/ or exp Sex Offenses/ or Intimate Partner Violence/ or Gender-Based Violence/ or Sex Work/ or exp Human papillomavirus 16/ or Human papillomavirus 18/ or Uterine Cervical Neoplasms/ or (reproductive or reproduction or family planning or sexual* or early marriage or child marriage or child bride? or female genital mutilation or female circumcision or menstruation or menstrual or puberty or contraception or contraceptive* or condom? or sexually transmitted infection* or sexually transmitted disease* or hiv or aids or coerc* or rape or forced sex or abortion? or intimate partner violence or gender based violence or sex work* or transactional sex or hpv or cervical cancer?).ti,ab,kw,kf.
48 exp Women's Health/ or exp Women's Health Services/ or Women/ or (wom#n? or female? or mother? or maternal or widow?).ti,ab,kw,kf.
49 exp Maternal Health Services/ or Pregnant Women/ or exp Pregnancy/ or (birth or childbirth or mother* or maternal* or maternity or preconception or prenatal* or perinatal* or antepartum or ante-partum or intrapartum or intra-partum or neonatal* or neo-natal* or postnatal* or post-natal* or postpartum or post-partum or pregnan*).ti,ab,kw,kf.

For peer review only

50 exp Infant/ or (fetus* or foetus* or fetal* or foetal* or baby or babies or neonate* or neo-nate* or newborn* or new-born* or infant*).ti,ab,kw,kf.
51 exp Child/ or exp Pediatrics/ or (child* or kid or kids or girl or girls or boy or boys or preschool* or pre-school* or kindergarten* or school age* or elementary school* or juvenile* or minors or p?ediatric? or first-grader* or second-grader* or third-grader* or fourth-grader* or fifth-grader* or sixth-grader* or seventh-grader* or eighth-grader* or middle school* or junior high*).ti,ab,kw,kf.
52 Adolescent/ or Young Adult/ or (teen* or youth* or adolescen* or juvenile* or (young adj2 (adult* or person* or individual* or people* or population*)) or youngster* or highschool* or ((secondary or high*) adj2 (school* or education)) or pubescen*).ti,ab,kw,kf.
53 or/47-52 [RMNCAH filter]
54 35 and 46 and 53

For peer review only

Supplementary File- Description of Included Studies

Author & Year	Country	Country Income Level	Primary Objective	Health Topic	SRMNCAH Priority	Study Design	Sample Characteristics	BCW Intervention Functions Summary	BCW Policy Categories Summary	Direction Of Effect
Abney-Roberts et al., 2015[28]	USA	High	To prevent sleep-related infant deaths by applying recommendations from the American Academy of Pediatrics	Newborn sleep	Newborn health/wellbeing or stillbirths	Quasi-experimental	150 healthcare providers (e.g., nurses and physicians from obstetrics, pediatrics and family medicine)	Education		Positive
Agapidaki et al., 2013[29]	Greece	High	To improve pediatricians' ability to identify and manage maternal depression by implementing a targeted education program	Maternal depression	Maternal health/wellbeing	Experimental	43 pediatricians	Education<br><br>Environmental Restructuring<br><br>Persuasion	Guidelines	Positive
Agency for Healthcare Research and Quality, 2016[89]	USA	High	To decrease maternal and neonatal adverse events, and to improve patient safety, team communication, and quality of care	Perinatal care	Maternal health/wellbeing<br><br>Newborn health/wellbeing or stillbirths	Observational	Labour & delivery units	Education<br><br>Environmental Restructuring<br><br>Training	Service Provision	Positive
Ainsworth et al., 2014[30]	USA	High	To reduce and prevent newborn falls	Newborn fall prevention	Newborn health/wellbeing or stillbirths	Quasi-experimental	Healthcare providers, mothers and their infants	Education<br><br>Environmental Restructuring	Regulation	Positive
Akter et al., 2009[90]	Bangladesh	Middle	To identify changes in antimicrobial use following the implementation of an educational intervention targeting physicians	Antimicrobial use	Child health/wellbeing	Quasi-experimental	All physicians working in pediatric wards at the participating hospital	Education		Positive
Al-Rafay & Al-Sharkawy, 2012[31]	Egypt	Middle	To evaluate changes in nurses' knowledge following an education program to address total parenteral nutrition guidelines	Newborn nutrition	Newborn health/wellbeing or stillbirths	Quasi-experimental	40 nurses	Education<br><br>Training	Guidelines	Positive
Alkon et al., 2014[32]	USA	High	To evaluate a nutrition and physical activity program implemented at childcare centres	Child nutrition and physical activity	Child health/wellbeing	Experimental	137 childcare providers from 17 childcare centres. Centres included a total of 552 children who were in the program (aged 3-5 years)	Education<br><br>Environmental Restructuring	Guidelines	Positive

Allen & Jeffery, 2006[91]	Nepal	Middle	To design and implement an educational newborn care program targeting healthcare providers to decrease infant morbidity and mortality	Newborn care	Newborn health/wellbeing or stillbirths	Quasi-experimental	30 healthcare providers (e.g., junior doctors, nurses, community health workers)	Education		Positive
Allen & Schafer, 2015[33]	USA	High	To implement practices which promote optimal infant feeding	Infant nutrition	Newborn health/wellbeing or stillbirths	Quasi-experimental	Healthcare providers, pregnant women, and new mothers and their infants	Education<br><br>Environmental Restructuring<br><br>Enablement	Service provision<br><br>Communication/ Marketing<br><br>Environmental/ Social Planning	Positive
Alton et al. 2006[99]	USA	High	To describe a pediatric patient safety program	Pediatric patient safety	Child health/wellbeing	Observational	Healthcare providers, health systems managers	Education<br><br>Environmental Restructuring	Service provision<br><br>Communication/ Marketing<br><br>Environmental/ Social Planning<br><br>Regulation	Positive
Alvarez et al., 2019[59]	Peru and Uganda	Low and middle	To determine the useability and usefulness of a workbook to provide support and guidance in global health systems	Maternal and newborn health	Maternal Health/Wellbeing<br><br>Newborn health/wellbeing or stillbirths	Qualitative	8 participants (e.g., researchers, policymakers or other stakeholders)	Education	Guidelines	Positive
Anaby et al., 2015[34]	Canada	High	To improve healthcare providers' knowledge and to change practice to support children with disabilities	Activities with children and youth with physical disabilities	Child health/wellbeing	Qualitative	14 healthcare providers (e.g., occupational therapists, physiotherapists, speech language pathologist)	Education	Service Provision	Positive
Ansbro et al., 2015[35]	Zambia	Middle	To explore healthcare providers' experience of using rapid syphilis testing	Syphilis	Sexual or reproductive health/rights	Qualitative	40 healthcare providers		Guidelines	Positive
Baer et al., 2011[36]	USA	High	To determine the effectiveness of a NICU blood transfusion and monitoring system 12 months following implementation	Neonatal blood transfusions	Newborn health/wellbeing or stillbirths	Quasi-experimental	Four NICUs	Environmental Restructuring<br><br>Enablement<br><br>Coercion<br><br>Restriction	Service Provision	Positive
Barnard et al., 2017[60]	USA	High	To identify barriers and enablers to establishing standing orders to vaccinate during pregnancy	Vaccination during pregnancy	Maternal health/wellbeing	Qualitative	38 healthcare providers and staff members (e.g., medical directors,	Education	Service Provision	Positive

							practice administrators, nurses, medical assistants)			
Becker-Haimes et al., 2017[61]	USA	High	To explore the acceptability and feasibility of a toolkit designed for healthcare providers working across mental health settings.	Youth mental health	Adolescent health/wellbeing	Mixed methods	Quantitative: 70 clinicians<br><br>Qualitative: Six randomly selected clinicians from the quantitative study	Education		Positive
Berglund et al., 2010[92]	Ukraine	Middle	To determine the effectiveness of the W.H.O's Effective Perinatal Care package implemented at maternity hospitals	Perinatal care	Maternal Health/Wellbeing	Quasi-experimental	Healthcare providers at three maternity hospitals (e.g., obstetricians, neonatologists, pediatricians, anesthesiologists, midwives, pediatric nurses)	Environmental Restructuring<br><br>Training<br><br>Enablement		Positive
Boyko et al., 2016[62]	Canada	High	To explore the usefulness of deliberative dialogue utilized by the Preventing Violence Across the Lifespan (PreVAiL) Research Network during a biennial team meeting	Child maltreatment	Child Health/Wellbeing	Mixed methods	44 participants in the deliberative dialogue (e.g., researchers, knowledge users, policymakers)	Education		Positive
Braddick et al., 2016[63]	Uganda	Low	To assess compliance with postpartum hemorrhage clinical guideline recommendations, and identify barriers and enablers to using the recommendations	Postpartum hemorrhage	Maternal Health/Wellbeing	Mixed methods	Quantitative: 154 births<br>Qualitative: 18 healthcare provider interviews (e.g., physicians, midwives)	Education	Guidelines	Neutral
Brennan et al., 2013[37]	Ghana	Middle	To increase nurses' knowledge and skills in pediatric resuscitation through the WHO Emergency Triage and Assessment Treatment program	Pediatric resuscitation	Child Health/Wellbeing	Quasi-experimental	41 nurses	Education<br><br>Training<br><br>Modelling		Positive
Brown et al., 2005[100]	UK	High	To identify the barriers creating delays in surgery and develop guidelines for management of undescended testes	Undescended testes	Newborn Health/Wellbeing or Stillbirths	Observational	Healthcare providers and hospital staff	Education<br><br>Environmental Restructuring	Service Provision<br><br>Guidelines	Positive

Burgoine et al., 2018[64]	Uganda	Low	To reduce neonatal mortality through the implementation of two neonatal health interventions	Neonatal mortality	Newborn Health/Wellbeing or Stillbirths	Quasi-experimental	57 healthcare providers (e.g., midwives, nurses, interns)	Education<br><br>Environmental Restructuring<br><br>Training<br><br>Enablement	Guidelines<br><br>Environmental/ Social Planning	Positive
Cameron et al., 2011[38]	Canada	High	To determine healthcare administrators' perceptions of the barriers and enablers in using a knowledge broker to support implementing measures aimed at children with cerebral palsy needs	Cerebral palsy	Child Health/Wellbeing	Qualitative	Healthcare administrators from 27 pediatric healthcare centres	Education<br><br>Environmental Restructuring	Service Provision	Positive
Carlo et al., 2009[101]	Zambia	Middle	To improve nurse midwives' knowledge and skills following completion of an American Academy of Pediatrics Neonatal Resuscitation Program	Neonatal resuscitation	Newborn Health/Wellbeing or Stillbirths	Observational	127 nurse midwives	Education<br><br>Training		Positive
Carmona et al., 2015[58]	USA	High	To improve healthcare providers' ability to provide high quality adolescent healthcare	Teen health	Adolescent Health/Wellbeing	Mixed methods	109 healthcare providers from 15 sites	Training	Guidelines	Positive
Close et al., 2016[65]	Madagascar	Low	To determine the effectiveness of a knowledge translation and implementation model aimed at training Peace Corps Volunteers to then train healthcare staff in rural communities	Neonatal resuscitation	Newborn Health/Wellbeing or Stillbirths	Quasi-experimental	10 Peace Corps Volunteers trained 42 healthcare providers in 10 communities	Training<br><br>Enablement		Positive
Crone et al., 2006[102]	Netherland s	High	To determine long-term use of a smoking cessation program to prevent passive smoking around children	Baby exposure to smoke in the household	Newborn Health/Wellbeing or Stillbirths	Observational	39 baby health clinic managers, 255 nurses and 68 physicians	Education<br><br>Training	Communication/ Marketing	Neutral
Cunningham & Cardy, 2020[88]	Canada	High	To improve speech language pathologists' knowledge and intention to utilize a tool designed to support participation-focused outcome measurements	Child speech	Child Health/Wellbeing	Quasi-experimental	46 speech language pathologists	Education<br><br>Persuasion		Positive
Daniels et al., 2008[118]	South Africa	Middle	To determine perceived and actual utilization of research in policy and legislation development	Eclampsia and pre-eclampsia	Maternal Health/Wellbeing	Qualitative	15 interviews with local researchers and policy makers	Education	Legislation	Positive

Davies et al., 2002[108]	Canada	High	To determine the effectiveness of an interactive, educational workshop to support nurses to implement fetal health surveillance guidelines	Labour and delivery	Maternal Health/Wellbeing	Mixed methods	Four hospitals sites (2 intervention, 2 control). Between 14-35 nurses from the intervention hospitals participated in each of the four education modules	Education<br><br>Environmental Restructuring<br><br>Training		Positive
Deorari et al., 2001[93]	India	Middle	To improve newborn health outcome and reduce incidence of newborn resuscitation by providing healthcare providers with neonatal resuscitation training	Neonatal resuscitation	Newborn Health/Wellbeing or Stillbirths	Quasi-experimental	Physicians and nurses at 14 hospitals	Environmental Restructuring<br><br>Training	Regulation	Positive
Di Noia et al., 2003[112]	USA	High	To promote a youth substance abuse prevention program through pamphlets, CD-ROMs, and the Internet to encourage use and recommendations for the program	Adolescent substance abuse prevention	Adolescent Health/Wellbeing	Experimental	188 staff members employed at junior high schools, youth-focused non-profit organizations, and government bodies aimed at preventing youth substance abuse	Education<br><br>Environmental Restructuring		Positive
Ding et al., 2008[94]	China	Middle	To reduce inappropriate antibiotic prescribing in the pediatric intensive care unit (PICU)	Antibiotic prescribing	Child Health/Wellbeing	Quasi-experimental	Pediatricians	Education<br><br>Environmental Restructuring	Guidelines<br><br>Environmental/ Social Planning	Positive
Dobbins et al., 2009[113]	Canada	High	To evaluate three KT strategies aimed at supporting healthy childhood bodyweight	Childhood obesity	Child Health/Wellbeing	Experimental	108 public health departments	Education<br><br>Environmental Restructuring<br><br>Training		Neutral
Edwards et al., 2016[66]	Jamaica, Kenya, Uganda and South Africa	Low and Middle	To improve HIV care by implementing nurse leadership hubs	HIV	Sexual or reproductive health/rights	Quasi-experimental	167 participants (e.g., nurses, staff nurses, managers, researchers, decision makers, community representatives)	Education<br><br>Environmental Restructuring<br><br>Training<br><br>Enablement	Communication/ Marketing	Neutral
English et al., 2010[109]	USA	High	To outline the design and implementation of a perinatal tobacco cessation program	Perinatal tobacco cessation	Maternal Health/Wellbeing	Mixed methods	11 lay case managers attended the first training session, 8 participated in the second session	Education<br><br>Training<br><br>Modelling		Neutral

For peer review only - http://bmjopen.bmj.com/site/about/guidelines.xhtml

Eriksson et al., 2017[86]	Vietnam	Middle	To identify barriers and enablers to implementation and sustainability of a knowledge translation strategy, the Neonatal Knowledge Into Practice (NeoKIP) trial	Neonatal mortality	Maternal Health/Wellbeing<br><br>Newborn Health/Wellbeing or Stillbirths	Qualitative	Interviews: 6 interviews with healthcare directors or representatives from healthcare facilities<br><br>Focus groups: 6 focus groups with previous stakeholders in the NeoKIP trial	Environmental Restructuring<br><br>Training		Positive
Farner et al., 2014[39]	USA	High	To improve healthcare providers' screening for critical congenital heart disease through the design, implement and evaluate an educational program	Newborn screening for critical congenital heart disease	Newborn Health/Wellbeing or Stillbirths	Quasi-experimental	215 nurses participated across 13 hospitals	Training<br><br>Modelling<br><br>Environmental restructuring<br><br>Education	Regulation	Positive
Finch et al., 2012[40]	Australia	High	To increase use of an intervention to encourage physical activity for children at childcare centres, and to improve childcare managers' knowledge of physical activity recommendations for children and perceived acceptability of the intervention	Childhood physical activity	Child Health/Wellbeing	Quasi-experimental	228 childcare centres participated in the intervention, 164 childcare centres were the control	Training<br><br>Enablement<br><br>Incentivisation		Positive
Finch et al., 2019[67]	Australia	High	To determine effectiveness of an intervention aimed at improving compliance with childcare nutrition guidelines in childcare centres	Childhood nutrition	Child Health/Wellbeing	Experimental	24 childcare centres participated in the intervention, 20 participated in the control from baseline data collection	Education<br><br>Training<br><br>Environmental Restructuring	Regulation	Neutral
George et al., 2018[68]	Sri Lanka	Middle	To hold a consultation with regional users of the WHO safe childbirth checklist to promote knowledge exchange, support improve maternal health outcomes, and offer high quality care during childbirth	Labour and delivery	Maternal Health/Wellbeing	Qualitative	60 participants (e.g., obstetricians, gynecologists, nurses, researchers, implementers, government officials, and 12 funders from low, middle, and high-income countries)	Education		Positive
Gera et al., 2019[69]	India	Middle	To evaluate an intervention aimed at improving newborn vaccination uptake in health facilities	Newborn vaccination	Newborn Health/Wellbeing or Stillbirths	Mixed Methods	141 public health facilities participated in the intervention<br><br>Qualitative: interviews with health managers	Training		Positive

Gichane et al., 2019[70]	Zambia	Middle	To outline the process of advocating for policy changes to allow for non-clinical healthcare workers to provide injectable contraception	Contraception/ Family planning	Sexual or reproductive health/rights	Editorial	Not reported	Education<br><br>Environmental Restructuring<br><br>Modelling	Legislation	Positive
Gilkey et al., 2019[71]	USA	High	To improve uptake of the HPV vaccine through use of a quality improvement program	HPV vaccination	Sexual or reproductive health/rights	Experimental	13 clinics received the intervention, while 12 were placed in the wait-list control group. 43 pediatric physicians participated across the intervention clinics, while 35 participated across the control clinics	Training<br><br>Incentivisation		Neutral
Goering & Wilson, 2002[107]	USA	High	To decrease variance and improve management of pre-term labour through the guideline implementation	Preterm labour	Maternal Health/Wellbeing	Observational	Nursing staff	Education<br><br>Enablement	Guidelines<br><br>Environmental/ Social planning	Positive
Goyet et al., 2014[41]	Cambodia	Middle	To identify barriers to KT strategies aimed at pneumonia	Pneumonia	Child Health/Wellbeing	Observational	Interventions targeted clinicians, health managers, policymakers	Education<br><br>Environmental Restructuring<br><br>Enablement		Neutral
Grady et al., 2019[72]	Australia	High	To determine effectiveness of a strategy aimed at improving compliance with dietary guidelines and daycare centres	Childhood nutrition	Child Health/Wellbeing	Quasi-experimental	27 daycare centres participated in the intervention services, 19 centres participated in the control services	Environmental Restructuring<br><br>Training<br><br>Enablement<br><br>Persuasion	Service Provision	Positive
Guillory, et al., 2017[73]	USA	High	To identify an appropriate implementation strategy for newborn screening for critical congenital heart disease across multiple hospitals	Newborn screening for critical congenital heart disease	Newborn Health/Wellbeing or Stillbirths	Quasi-experimental	117 physicians, hospital administrators and other healthcare professionals	Education<br><br>Environmental Restructuring<br><br>Training<br><br>Modelling		Positive

Hardee et al., 2019[74]	Nigeria and Uganda	Low and Middle	To determine effectiveness of two interventions which utilize a voluntary, rights-based family planning (VRBFP) approach to measure health and rights outcomes	Family planning	Sexual or reproductive health/rights	Mixed Methods	15 intervention healthcare facilities<br><br>Qualitative: 16 interviews with healthcare providers, health facility managers, supervisors, and representatives from collaborating human rights organizations<br><br>Focus Groups: male champions for the project, and facility and community healthcare workers	Education<br><br>Environmental Restructuring<br><br>Training<br><br>Enablement		Positive
Hulton et al., 2014[42]	Ethiopia, Ghana, Malawi, Nigeria, Sierra Leone, and Tanzania	Low and Middle	To outline and describe the Evidence for Action (E4A) program and its contributions to reduce rates of maternal and newborn mortality	Maternal and newborn mortality	Maternal Health/Wellbeing<br><br>Newborn health/wellbeing or stillbirths	Observational	Case studies of each of the E4A countries: Ethiopia, Ghana, Malawi, Nigeria, Sierra Leone, and Tanzania	Persuasion<br><br>Coercion	Communication/ Marketing<br><br>Guidelines<br><br>Regulation	Positive
Jeffery et al., 2004[95]	Macedonia	Middle	To outline the design and implementation of an education intervention aimed at improving perinatal health practices	Maternal and newborn mortality	Maternal Health/Wellbeing<br><br>Newborn health/wellbeing or stillbirths	Quasi-experimental	115 physicians and nurses	Education<br><br>Training		Positive
Jones et al., 2015[43]	Australia	High	To determine effectiveness of a complex intervention aimed at improving childcare nutrition and physical activity practices in childcare centres	Childhood nutrition and physical activity	Child Health/Wellbeing	Experimental	62 childcare centres participated in the intervention, while 60 centres served as the control	Education<br><br>Environmental Restructuring<br><br>Training<br><br>Enablement	Communication/ Marketing	Neutral
Kapungu et al., 2013[44]	Ghana	Middle	To reduce postpartum hemorrhage by designing and implementing a community-based misoprostol distribution intervention	Postpartum hemorrhage	Maternal Health/Wellbeing	Observational	74 healthcare providers	Education<br><br>Environmental Restructuring<br><br>Training<br><br>Enablement		Positive
Kingsnorth et al., 2020[85]	Canada	High	To address barriers and enablers to a knowledge translation intervention to improve clinic care	Children with physical disabilities	Child Health/Wellbeing	Observational	Healthcare providers	Education<br><br>Environmental Restructuring		Positive

			provided at a pediatric rehabilitation hospital	and complex health needs				Enablement<br><br>Persuasion		
Kraft et al., 2018 [75]	Ethiopia and Senegal	Low and Middle	To describe the perceived use and impact of using the WHO family planning guidance and tools	Family planning	Sexual or reproductive health/rights	Qualitative	Interviews with: 5 WHO regional advisers, 6 policy makers, 7 disseminators, 4 implementers and 4 end-users	Education	Guidelines	Positive
Lang et al., 2015[45]	USA	High	To improve healthcare providers' use of trauma-focused cognitive behavioural therapy and improve outcomes in children through the use of evidence-based practice	Mental health	Child Health/Wellbeing	Mixed methods	Across 16 participating agencies, 77 clinicians, 33 supervisors, and 23 senior leaders participated in training. 734 children across the agencies used the therapy approach	Education<br><br>Training<br><br>Persuasion		Positive
Makkar et al., 2016[76]	Australia	High	To test strategies to improve policymakers' use of Web Centre for Informing Policy in Health with Evidence from Research (CIPHER) usage and engagement with research evidence	Various sexual health topics (e.g., sexually transmissible infections, menopause, breast and cervical screening, and men's sexual health)	Sexual or reproductive health/rights	Observational	223 policymakers from 27 organisations accessed the platform during the study period	Education<br><br>Environmental Restructuring<br><br>Persuasion	Communication/ Marketing	Positive
Margolis et al., 2001[103]	USA	High	To improve the processes of health services and care delivery, particularly between healthcare providers and families, to support child health outcomes	Child health outcomes	Child Health/Wellbeing	Observational	8 pediatric and family healthcare practices participated	Education<br><br>Environmental Restructuring<br><br>Training<br><br>Enablement	Service Provision<br><br>Guidelines<br><br>Regulation	Positive
Mello et al., 2013[46]	USA	High	To identify adoption and implementation strategies for integrating an alcohol screening tool into regular practice for pediatric trauma patients	Alcohol use	Adolescent Health/Wellbeing	Mixed methods	7 pediatric trauma centres. A key informant was identified at each site<br><br>Qualitative: monthly check-in and feedback conversations with project managers at each site	Education<br><br>Training<br><br>Enablement	Service Provision	Positive

Melnyk et al., 2010[114]	USA	High	To implement the evidence-based Creating Opportunities for Personal Empowerment program and determine its effectiveness on nurses' beliefs of evidence-based practice	Premature birth	Newborn Health/Wellbeing or Stillbirths	Experimental	81 nurses (48 participated in implementation group and 33 participated in the control group)	Education<br><br>Training	Service Provision	Positive
Mody et al., 2013[47]	Bangladesh, China, Indonesia, Myanmar, Mongolia, Solomon Islands, Tonga, Vanuatu and Vietnam	Middle	To assess the use of family guidelines and associated tools as part of the Strategic Partnership Program	Family planning	Sexual or reproductive health/rights	Mixed methods	Workshop participants: program managers, Ministry of Health staff, clinicians and regional and country office staff from WHO/UNFPA.<br><br>Questionnaire: provided to Ministries of Health	Education<br><br>Training	Communication/ Marketing<br><br>Guidelines	Positive
Okonofua et al., 2011[57]	Nigeria	Middle	To describe the findings from multiple advocacy activities designed to provide free health services to pregnant women and children	Maternal and child mortality	Maternal Health/Wellbeing Child Health/Wellbeing	Observational	Interviews with 6 State Governors and/or Commissioners of Health	Education<br><br>Modelling<br><br>Persuasion	Communication/ Marketing<br><br>Guidelines	Positive
Ongolo-Zogo et al., 2018[77]	Cameroon and Uganda	Low and Middle	To assess how KT platforms have supported Millennium Development Goals through health system policy-making and decision making processes	Maternal and child health	Maternal Health/Wellbeing<br><br>Child Health/Wellbeing	Observational	Stakeholders and decision makers	Education<br><br>Environmental Restructuring<br><br>Training		Positive
Pariyo et al., 2005[104]	Uganda	Low	To determine the effects of scaling-up an intervention aimed at the integrated management of childhood illnesses to improve health outcomes in young children (under 5)	Childhood illness	Child Health/Wellbeing	Observational	316 healthcare facilities across 10 study districts. 427 healthcare workers participated across these facilities	Education<br><br>Training<br><br>Enablement		Neutral
Rowe et al., 2009[115]	Benin	Middle	To evaluate the effectiveness of additional training supports developed for healthcare providers who completed training in Integrated Management of Childhood Illness on child health outcomes	Childhood illness	Child Health/Wellbeing	Experimental	1244 consultations for any illness across 16 communes. Consultations were conducted by 267 healthcare workers at 114 health facilities.	Education<br><br>Environmental Restructuring<br><br>Training<br><br>Incentivisation		Neutral
Russell et al., 2010[110]	Canada	High	To evaluate knowledge brokers and their impact in a knowledge translation intervention and measurement tools to understand motor function in children with cerebral palsy	Cerebral palsy	Child Health/Wellbeing	Mixed methods	122 physiotherapists across three provinces	Education<br><br>Environmental Restructuring	Service Provision	Positive

For peer review only - http://bmjopen.bmj.com/site/about/guidelines.xhtml

Schreiber et al., 2009[105]	USA	High	The purpose of this study was to identify, implement, and evaluate strategies to support integrating scientific research evidence into clinical decision making	Physiotherapy	Child Health/Wellbeing	Observational	5 pediatric physiotherapists	Education<br><br>Environmental Restructuring<br><br>Training<br><br>Incentivisation		Positive
Schreiber et al., 2015[48]	USA	High	To outline a KT program designed to increase physiotherapists' knowledge and use of standardized outcome measures	Physiotherapy	Child Health/Wellbeing	Observational	17 physiotherapists from a pediatric outpatient facility	Education<br><br>Environmental Restructuring<br><br>Training<br><br>Modelling<br><br>Enablement<br><br>Persuasion		Positive
Schwartz et al., 2015[49]	USA	High	To use a multi-stakeholder partnership to develop and implement evidence-based breastfeeding support and training strategies	Breastfeeding	Maternal Health/Wellbeing	Quasi-experimental	8 health centres with predominantly culturally diverse patients implemented the 10 steps to support breastfeeding	Education<br><br>Environmental Restructuring<br><br>Training	Service Provision<br><br>Communication/ Marketing,<br><br>Guidelines<br><br>Environmental/ Social Planning	Positive
Segre et al., 2011[50]	USA	High	To describe the development and implementation of the train-the-trainer program aimed at maternal depression screening	Perinatal depression	Maternal Health/Wellbeing	Observational	42 individuals were trained from 32 maternal and child health agencies	Environmental Restructuring<br><br>Training		Positive
Segre et al., 2018[78]	USA	High	To describe a research and government partnership implementing 'listening visits' in health centres to support maternal mental health	Postpartum depression	Maternal Health/Wellbeing	Editorial	18 maternal health clinics	Education<br><br>Training<br><br>Enablement	Guidelines	Positive
Senarath et al., 2007[116]	Sri Lanka	Middle	To evaluate a program aimed at training healthcare providers to improve newborn care practices	Newborn care	Newborn Health/Wellbeing or Stillbirths	Experimental	120 healthcare providers. 59 participated in the intervention (27 midwives, 19 nurses and 13 physicians) while 61 participated in the control (26 midwives, 19 nurses and 16 physicians)	Education<br><br>Training		Positive

Seward et al., 2018[79]	Australia	High	To evaluate the effectiveness of a complex intervention aimed at improving compliance with child nutrition guidelines and children's dietary intake	Childhood nutrition	Child Health/Wellbeing	Experimental	45 childcare centres- 25 participated in the intervention and 20 participated in the control	Education<br><br>Environmental Restructuring<br><br>Persuasion	Guidelines	Positive
Simioni et al., 2017[87]	Argentina	Middle	To support the regionalization of a perinatal health strategy policy as a to improve health outcomes	Maternal and premature/ newborn health	Maternal Health/Wellbeing<br><br>Newborn Health/Wellbeing or Stillbirths	Mixed methods	Participants completed the first workshop and 49 participated in the second workshop	Training	Environmental/ Social Planning	Positive
Simmons, 2007[106] (Chapter 2)	Viet Nam	Middle	To describe the process of scaling up an intervention to provide injectable contraceptives to improve quality of care and family planning services	Contraceptio n/ family planning	Sexual or reproductive health/rights	Observational	21 provinces in Viet Nam - Each provincial team included provincial health and family planning sector representatives	Education	Guidelines	Neutral
Simmons, 2007[111] (Chapter 8)	Brazil, Bolivia, Chile	Middle and High	To implement training the trainer methods to build capacity and provide innovative training in the area of reproductive health services	Reproductive health services	Sexual or reproductive health/rights	Mixed methods	Brazil: 9 training centre teams. 98 trainers were trained, and 1921 providers trained<br><br>Bolivia: 8 training centre teams. 34 trainers were trained, and 741 providers trained<br><br>Chile: 3 training centre teams. 21 trainers were trained, and 395 providers trained	Education<br><br>Environmental Restructuring<br><br>Training	Service Provision	Positive
Singh et al., 2013[51]	Ghana	Middle	To test local change ideas which could be scaled up later as part of a national child survival improvement project	Maternal and infant health	Maternal Health/Wellbeing<br><br>Newborn Health/Wellbeing or Stillbirths	Quasi-experimental	27 healthcare facilities	Education	Environmental/. Social Planning	Positive
Snelgrove-Clarke, 2010[78]	Canada	High	To evaluate two educational interventions to improve nurses' use of clinical guidelines during labour and delivery	Labour and delivery	Maternal Health/Wellbeing	Experimental	93 nurses participated in the first intervention and 62 nurses participated in the second intervention	Education<br><br>Enablement	Guidelines	Neutral

For peer review only

Sobel et al., 2011[116]	Philippines	Middle	To evaluate the impact of a training workshop for healthcare providers to provide newborns with a Hepatitis B vaccination, in line with a new vaccination policy	Newborn Hepatitis B vaccination	Newborn Health/Wellbeing or Stillbirths	Mixed methods	45 health centre staff, 120 community healthcare workers and 23 healthcare providers from 9 healthcare centers	Education<br><br>Environmental Restructuring	Guideline	Positive
Sundaram et al., 2015[79]	Ghana	Middle	To determine the effectiveness of a multi-stakeholder partnership program developed to provide comprehensive abortion and post-abortion care services	Abortion	Sexual or reproductive health/rights	Quasi-experimental	457 healthcare providers (116 physicians and 341 midwives, nurses, medical assistants) participated across 166 healthcare facilities.<br><br>The intervention group comprised 197 participants from 64 healthcare facilities. The first control group included 148 participants from 58 healthcare facilities, and the second control group included 112 participants across 44 healthcare facilities.	Environmental Restructuring<br><br>Training	Service Provision<br><br>Guidelines<br><br>Legislation	Positive
Tarasoff et al., 2014[87]	Canada	High	To evaluate the effectiveness of a theatre-based KT intervention design to improve healthcare providers' knowledge and attitudes of assisted human reproduction for LGBTQ people	Family planning	Sexual or reproductive health/rights	Qualitative	30 health professionals attended the workshop (e.g., physicians, nurses, social workers, health managers)	Training	Service Provision	Positive
Twum-Danso et al., 2014[55]	Ghana	Middle	To test the feasibility and scale up of a postnatal care policy to improve care	Postnatal care	Newborn Health/Wellbeing or Stillbirths	Quasi-experimental	30 teams from 27 rural health facilities	Education	Guidelines	Positive
Uneke et al., 2017[81]	Nigeria	Middle	To improve researchers and policymakers' the knowledge and capacity to use KT to promote evidence-informed policymaking in maternal, newborn and child health and wellbeing	Maternal, newborn and child health	Child Health/Wellbeing<br><br>Maternal Health/Wellbeing	Quasi-experimental	38 participants (e.g., researchers, government members) attended the workshop	Education		Positive

					Newborn Health/Wellbeing or Stillbirths					
Uneke et al., 2018[80]	Nigeria	Middle	To improve researchers and policymakers' competence to apply KT to promote evidence-informed policymaking in maternal, newborn and child health and wellbeing	Maternal, newborn and child health	Child Health/Wellbeing<br><br>Maternal Health/Wellbeing<br><br>Newborn Health/Wellbeing or Stillbirths	Quasi-experimental	45 participants (e.g., research team members, project managers or steering committee members, government health agency members, NGO and charity members)	Education<br><br>Environmental Restructuring		Positive
Uskun et al., 2008[96]	Turkey	Middle	To improve healthcare providers' knowledge of immunization and increase immunization coverage through an education intervention	Childhood vaccination	Child Health/Wellbeing	Quasi-experimental	229 primary healthcare workers (89 physicians,88 midwives, 38 health officers, 14 nurse)	Education		Positive
Vlad et al., 2016[82]	India	Middle	To describe a multi-stakeholder partnership to develop comprehensive clinical guidelines to reduce maternal mortality	Postpartum haemorrhage	Maternal Health/Wellbeing	Observational	400 healthcare staff working in 8 maternity wards	Environmental Restructuring<br><br>Training<br><br>Enablement	Environmental/ Social Planning	Positive
Warren et al., 2010[97]	Kenya	Middle	To evaluate the impact of a new postnatal intervention to improve healthcare workers' counselling on maternal and newborn health	Maternal and newborn health	Maternal Health/Wellbeing<br><br>Newborn Health/Wellbeing or Stillbirths	Quasi-experimental	76 healthcare providers from four health facilities	Education	Service Provision	Positive
Wolfe et al., 2014[56]	USA	High	To develop evidence-based guidelines and complementary training for healthcare providers to support sharing life-altering information in pediatric settings	Health communication	Child Health/Wellbeing	Quasi-experimental	142 healthcare providers (54 physicians, 32 fellows and residents, 18 medical students, 14 health assistants, 15 nurses, 6 physiotherapists, 3 social workers)	Education<br><br>Environmental Restructuring	Guidelines<br><br>Environmental/ Social planning	Positive

Wolfenden et al., 2017[83]	Australia	High	To evaluate the implementation and compliance with a healthy school canteen policy, nutrient quality of foods provided at the canteens	Childhood nutrition	Child Health/Wellbeing	Experimental	70 primary schools with canteens (35 assigned to both intervention and control groups)	Education<br><br>Environmental Restructuring<br><br>Training<br><br>Enablement<br><br>Persuasion<br><br>Incentivisation	Communication/ Marketing<br><br>Environmental/ Social Planning	Positive
Yoong et al., 2016[84]	Australia	High	This study aimed to examine the impact of providing printed educational materials on childcare service cooks' intentions to use nutritional guidelines and provide fruit and vegetables on their menu	Childhood nutrition	Child Health/Wellbeing	Experimental	77 childcare centres (38 acted as the control and 39 participated in the intervention)	Education	Communication/ Marketing	Positive

For peer review only

Preferred Reporting Items for Systematic reviews and Meta-Analyses extension for Scoping Reviews (PRISMA-ScR) Checklist

SECTION	ITEM	PRISMA-ScR CHECKLIST ITEM	REPORTED ON PAGE #
**TITLE**			
Title	1	Identify the report as a scoping review.	1
**ABSTRACT**			
Structured summary	2	Provide a structured summary that includes (as applicable): background, objectives, eligibility criteria, sources of evidence, charting methods, results, and conclusions that relate to the review questions and objectives.	3
**INTRODUCTION**			
Rationale	3	Describe the rationale for the review in the context of what is already known. Explain why the review questions/objectives lend themselves to a scoping review approach.	6
Objectives	4	Provide an explicit statement of the questions and objectives being addressed with reference to their key elements (e.g., population or participants, concepts, and context) or other relevant key elements used to conceptualize the review questions and/or objectives.	6
**METHODS**			
Protocol and registration	5	Indicate whether a review protocol exists; state if and where it can be accessed (e.g., a Web address); and if available, provide registration information, including the registration number.	6-7
Eligibility criteria	6	Specify characteristics of the sources of evidence used as eligibility criteria (e.g., years considered, language, and publication status), and provide a rationale.	7
Information sources*	7	Describe all information sources in the search (e.g., databases with dates of coverage and contact with authors to identify additional sources), as well as the date the most recent search was executed.	7-8
Search	8	Present the full electronic search strategy for at least 1 database, including any limits used, such that it could be repeated.	8
Selection of sources of evidence†	9	State the process for selecting sources of evidence (i.e., screening and eligibility) included in the scoping review.	8
Data charting process‡	10	Describe the methods of charting data from the included sources of evidence (e.g., calibrated forms or forms that have been tested by the team before their use, and whether data charting was done independently or in duplicate) and any processes for obtaining and confirming data from investigators.	9
Data items	11	List and define all variables for which data were sought and any assumptions and simplifications made.	9
Critical appraisal of individual	12	If done, provide a rationale for conducting a critical appraisal of included sources of evidence; describe	NA

SECTION	ITEM	PRISMA-ScR CHECKLIST ITEM	REPORTED ON PAGE #
sources of evidence§		the methods used and how this information was used in any data synthesis (if appropriate).	
Synthesis of results	13	Describe the methods of handling and summarizing the data that were charted.	9-10
**RESULTS**			
Selection of sources of evidence	14	Give numbers of sources of evidence screened, assessed for eligibility, and included in the review, with reasons for exclusions at each stage, ideally using a flow diagram.	10
Characteristics of sources of evidence	15	For each source of evidence, present characteristics for which data were charted and provide the citations.	10-13
Critical appraisal within sources of evidence	16	If done, present data on critical appraisal of included sources of evidence (see item 12).	NA
Results of individual sources of evidence	17	For each included source of evidence, present the relevant data that were charted that relate to the review questions and objectives.	Supplementary file
Synthesis of results	18	Summarize and/or present the charting results as they relate to the review questions and objectives.	10-18
**DISCUSSION**			
Summary of evidence	19	Summarize the main results (including an overview of concepts, themes, and types of evidence available), link to the review questions and objectives, and consider the relevance to key groups.	18-22
Limitations	20	Discuss the limitations of the scoping review process.	22-23
Conclusions	21	Provide a general interpretation of the results with respect to the review questions and objectives, as well as potential implications and/or next steps.	23-24
**FUNDING**			
Funding	22	Describe sources of funding for the included sources of evidence, as well as sources of funding for the scoping review. Describe the role of the funders of the scoping review.	25

JBI = Joanna Briggs Institute; PRISMA-ScR = Preferred Reporting Items for Systematic reviews and Meta-Analyses extension for Scoping Reviews.

* Where *sources of evidence* (see second footnote) are compiled from, such as bibliographic databases, social media platforms, and Web sites.

† A more inclusive/heterogeneous term used to account for the different types of evidence or data sources (e.g., quantitative and/or qualitative research, expert opinion, and policy documents) that may be eligible in a scoping review as opposed to only studies. This is not to be confused with *information sources* (see first footnote).

‡ The frameworks by Arksey and O'Malley (6) and Levac and colleagues (7) and the JBI guidance (4, 5) refer to the process of data extraction in a scoping review as data charting.

§ The process of systematically examining research evidence to assess its validity, results, and relevance before using it to inform a decision. This term is used for items 12 and 19 instead of "risk of bias" (which is more applicable to systematic reviews of interventions) to include and acknowledge the various sources of evidence that may be used in a scoping review (e.g., quantitative and/or qualitative research, expert opinion, and policy document).

From: Tricco AC, Lillie E, Zarin W, O'Brien KK, Colquhoun H, Levac D, et al. PRISMA Extension for Scoping Reviews (PRISMAScR): Checklist and Explanation. Ann Intern Med. 2018;169:467–473. doi: 10.7326/M18-0850.

For peer review only - http://bmjopen.bmj.com/site/about/guidelines.xhtml

Knowledge Translation Strategies for Policy and Action Focused on Sexual, Reproductive, Maternal, Newborn, Child & Adolescent Health and Wellbeing: A Rapid Scoping Review

Journal:	*BMJ Open*
Manuscript ID	bmjopen-2021-053919.R1
Article Type:	Original research
Date Submitted by the Author:	28-Sep-2021
Complete List of Authors:	Curran, Janet; Dalhousie University, Nursing; IWK Health Centre, Pediatrics<br>Gallant, Allyson; Dalhousie University, Faculty of Health<br>Wong, Helen ; Dalhousie University, Faculty of Health<br>Shin, Hwayeon Danielle; Dalhousie University, School of Nursing<br>Urquhart, Robin; Dalhousie University, Department of Community Health and Epidemiology<br>Kontak, Julia; Maritime SPOR SUPPORT Unit<br>Wozney, Lori; Nova Scotia Health<br>Boulos, Leah; Maritime SPOR SUPPORT Unit<br>Bhutta, Zulfiqar; The Hospital for Sick Children, Centre for Global Child Health<br>Langlois, Etienne V.; World Health Organization, The Partnership for Maternal, Newborn & Child Health
**Primary Subject Heading**:	Global health
Secondary Subject Heading:	Health services research, Health policy, Paediatrics, Sexual health, Reproductive medicine
Keywords:	Community child health < PAEDIATRICS, Quality in health care < HEALTH SERVICES ADMINISTRATION & MANAGEMENT, PUBLIC HEALTH

I, the Submitting Author has the right to grant and does grant on behalf of all authors of the Work (as defined in the below author licence), an exclusive licence and/or a non-exclusive licence for contributions from authors who are: i) UK Crown employees; ii) where BMJ has agreed a CC-BY licence shall apply, and/or iii) in accordance with the terms applicable for US Federal Government officers or employees acting as part of their official duties; on a worldwide, perpetual, irrevocable, royalty-free basis to BMJ Publishing Group Ltd ("BMJ") its licensees and where the relevant Journal is co-owned by BMJ to the co-owners of the Journal, to publish the Work in this journal and any other BMJ products and to exploit all rights, as set out in our licence.

The Submitting Author accepts and understands that any supply made under these terms is made by BMJ to the Submitting Author unless you are acting as an employee on behalf of your employer or a postgraduate student of an affiliated institution which is paying any applicable article publishing charge ("APC") for Open Access articles. Where the Submitting Author wishes to make the Work available on an Open Access basis (and intends to pay the relevant APC), the terms of reuse of such Open Access shall be governed by a Creative Commons licence – details of these licences and which Creative Commons licence will apply to this Work are set out in our licence referred to above.

Other than as permitted in any relevant BMJ Author's Self Archiving Policies, I confirm this Work has not been accepted for publication elsewhere, is not being considered for publication elsewhere and does not duplicate material already published. I confirm all authors consent to publication of this Work and authorise the granting of this licence.

For peer review only

Knowledge Translation Strategies for Policy and Action Focused on Sexual, Reproductive, Maternal, Newborn, Child & Adolescent Health and Wellbeing: A Rapid Scoping Review

Janet A. Curran*[1,2] (jacurran@dal.ca) *Corresponding author https://orcid.org/0000-0001-9977-0467

Allyson J. Gallant[3] https://orcid.org/0000-0002-2933-7470

Helen Wong[3] https://orcid.org/0000-0001-6827-0662

Hwayeon Danielle Shin[1] https://orcid.org/0000-0003-4037-4464

Robin Urquhart[4] https://orcid.org/0000-0001-8864-5716

Julia Kontak[5] https://orcid.org/0000-0002-9104-0678

Lori Wozney[6] https://orcid.org/0000-0003-4280-3322

Leah Boulos[5] https://orcid.org/0000-0002-9849-383X

Zulfiqar A. Bhutta[7] https://orcid.org/0000-0003-0637-599X

Etienne V. Langlois[8] https://orcid.org/0000-0002-4853-2511

AFFILIATIONS:

[1] School of Nursing, Dalhousie University, 5869 University Avenue, Halifax, Nova Scotia, Canada B3H 4R2

[2] IWK Health Centre, Halifax, Nova Scotia, Canada

[3] Faculty of Health, Dalhousie University, Halifax, Nova Scotia, Canada

[4] Department of Community Health and Epidemiology, Dalhousie University, Halifax, Nova Scotia, Canada

[5] Maritime SPOR SUPPORT Unit, Halifax, Nova Scotia, Canada

[6] Nova Scotia Health, Nova Scotia, Canada

[7] Centre for Global Child Health, The Hospital for Sick Children (SickKids), Toronto, Ontario, Canada

For peer review only

[8] Partnership for Maternal, Newborn & Child Health (PMNCH), World Health Organisation, Geneva, Switzerland

For peer review only

ABSTRACT

Objective: The aim of this study was to identify knowledge translation (KT) strategies aimed at improving sexual, reproductive, maternal, newborn, child and adolescent health (SRMNCAH) and wellbeing.

Design: Rapid scoping review.

Search Strategy: A comprehensive and peer-reviewed search strategy was developed and applied to four electronic databases: MEDLINE ALL, Embase, CINAHL, and Web of Science. Additional searches of grey literature were conducted to identify KT strategies aimed at supporting SRMNCAH. KT strategies and policies published in English from January 2000- May 2020 onwards were eligible for inclusion.

Results: Only 4% of included 90 studies were conducted in low-income countries with the majority (52%) conducted in high-income countries. Studies primarily focused on maternal newborn or child health and wellbeing. *Education* (81%), including staff workshops and education modules, was the most commonly identified intervention component from the KT interventions. Low- and middle-income countries were more likely to include civil society organizations, government and policy makers as stakeholders compared to high-income countries. Reported barriers to KT strategies included limited resources and time constraints, while enablers included stakeholder involvement throughout the KT process.

Conclusion: We identified a number of gaps among KT strategies for SRMNCAH policy and action, including limited focus on adolescent, sexual and reproductive health and rights and SRMNCAH financing strategies. There is a need to support stakeholder engagement in KT interventions across the continuum of SRMNCAH services. Researchers and policy makers should consider enhancing efforts to work with multi-sectoral stakeholders to implement future KT strategies and policies to address SRMNCAH priorities.

Registration: The rapid scoping review protocol was registered on Open Science Framework on June 16th, 2020 (https://osf.io/xpf2k).

KEYWORDS: knowledge translation, sexual health, reproductive health, maternal health, newborn health, child health, adolescent health

Strengths and Limitations

- Our review identified a broad range of KT strategies and policies aimed at improving sexual, reproductive, maternal, newborn, child and adolescent health (SRMNCAH) and wellbeing published since January 2020
- KT strategies and policies were mapped to the Behaviour Change Wheel to identify and describe intervention and policy elements included in the strategies
- The terminology around knowledge translation varies across countries and health areas
- As a rapid scoping review, full text and grey literature review and data extraction were carried out by a single reviewer and verified by a second reviewer
- Although we conducted a systematic search of the grey literature it is possible our findings were impacted by our strict inclusion criteria and potential publication bias

For peer review only

INTRODUCTION

Progress towards sexual, reproductive, maternal, newborn, child and adolescent health (SRMNCAH) has been highly inequitable to date.[1,2] With the current COVID-19 pandemic, there have been substantive and unprecedented disruptions in essential SRMNCAH services,[3] including emerging data on increased maternal mortality, stillbirth rates, ruptured ectopic pregnancies, unintended pregnancies, maternal depression and limited access to contraceptives.[4,5] The greatest disruptions to essential healthcare services are witnessed in low-income countries.[6] As such, special efforts are needed to support evidence-based interventions to prevent further harm, reduce preventable deaths and morbidity, and promote equitable distribution of essential interventions for SRMNCAH.

There is a lack of equitable distribution of human resources and essential policy adoptions for SRMNCAH in most countries.[7] COVID-19 has also brought to the forefront the need to develop and implement multi-sectoral interventions using a whole-of-government approach.[8] Improving SRMNCAH across countries of different income levels will require collective action in terms of generating, sharing, brokering, and implementing new knowledge through cross-sectoral and interdisciplinary initiatives.[9] The Partnership for Maternal, Newborn & Child Health (PMNCH), hosted by the World Health Organization, is a global partnership designed to address these SRMNCAH inequities, and improve the health and well-being of all women, children and adolescents.[10]

Interventions shown to be effective by scientific endeavours require efforts to integrate evidence into policy and action. Knowledge translation (KT) is “a dynamic and iterative process that includes synthesis, dissemination, exchange and ethically-sound application of knowledge to improve the health, provide more effective health services, and products and strengthen the healthcare system.”[11] KT interventions can support this process

141 by facilitating the uptake of evidence into policy and practice targeting change at the level
142 professional, institutional or policy level. There has been a growing number of KT
143 interventions, as well as frameworks, theories, and models to guide the selection of KT
144 interventions.[12–14] However, the range of KT strategies related to SRMNCAH improvements
145 remains unknown.

146 No review to date has explored the range of KT interventions utilized at the level of
147 health system, policy, or practice specifically addressing the continuum of SRMNCAH. In
148 light of the global call for action to sustain SRMNCAH, it is critical to understand the
149 implementation of KT strategies that promote evidence-based policy and practice for
150 SRMNCAH.

151 **Objectives**

152 The aim of this rapid scoping review was to identify existing literature related to KT
153 strategies that promote the uptake of evidence into policy and action focused on improving
154 SRMNCAH and wellbeing. To achieve this aim, four questions were addressed:

155 1. What are the common KT strategies and activities used to promote the use of
156 evidence to inform policy and action to improve SRMNCAH and wellbeing?
157 2. How are stakeholders involved in designing or implementing these KT
158 strategies and activities?
159 3. What are the commonly reported outcomes of KT strategies and activities to
160 promote the use of evidence in SRMNCAH and wellbeing?
161 4. What are the commonly reported barriers and enablers for using KT strategies
162 and activities to promote the use of evidence in SRMNCAH and wellbeing?

163 **METHODS**

only

This rapid scoping review follows the methodological guidance developed by the Joanna Briggs Institute and is reported according to the Preferred Reporting Items for Systematic Reviews and Meta-Analyses - Extension for Scoping Reviews (PRISMA-ScR).[15,16] The rapid scoping review protocol was registered on Open Science Framework on June 16th, 2020 (https://osf.io/xpf2k).

Inclusion and Exclusion Criteria

All study designs were eligible for inclusion in the review. Studies were excluded if they focused on basic science or clinical management of women, newborn, child or adolescent aspects of health or wellbeing. Systematic reviews were also excluded; however, the reference lists of relevant reviews were examined to identify additional potential studies for inclusion. Studies published from 2000 onwards and published in English were eligible for inclusion.

Studies reporting a KT strategy aimed at supporting or improving health systems or policy decisions to support SRMNCAH and wellbeing were eligible for inclusion. KT strategies aimed at patients, caregivers, healthcare providers, healthcare management, health systems, policy makers, civil society organizations and funder or donors, within or outside the health sector, were also eligible as long as it was in the context of SRMNCAH and wellbeing. Studies that targeted these stakeholders outside of SRMNCAH and wellbeing were excluded. KT strategies addressing HIV were excluded, unless specifically focused on SRMNCAH. Study outcomes relating to the effectiveness or implementation of the KT strategy or activity and SRMNCAH were included. Studies that did not report primary outcomes relevant to KT or SRMNCAH were excluded.

Search Strategy and Information Sources

A comprehensive search strategy was developed with support from an experienced library scientist. The search strategy was peer-reviewed by a second library scientist using the

For peer review only

Peer Review of Electronic Search Strategy (PRESS) guidelines to ensure a comprehensive and high-quality search strategy was developed.[17] An electronic database search of MEDLINE ALL (Ovid), Embase (Elsevier Embase.com), CINAHL with Full Text (EBSCOhost), and Web of Science (SCI-EXPANDED, SSCI, A&HCI, CPCI-S, CPCI-SSH, ESCI; Clarivate) was executed on May 29, 2020, and results were limited from January 2000 to the search date (see Supplementary File 1). No search filters or other limits were applied. Search strategy citations were imported into Covidence, an online systematic review management software, and duplicates were removed automatically in Covidence prior to screening.[18] Reference lists of all included studies, as well as those of any relevant systematic reviews, were screened by one reviewer and verified by a second. Additionally, a search of grey literature was undertaken by a reviewer in July 2020. Search terms were applied in Google and relevant website links were clicked through to identify any reports or literature. The reviewer clicked through each relevant website and used reference chaining within the website to ensure any and all relevant literature was identified. Google results were browsed until the reviewer went two pages (20 results) without clicking on a potentially relevant result. The website URL links were compiled and verified for inclusion by another team member.

Selection of Sources of Evidence

Reviewers independently screened titles and abstracts against the inclusion criteria, and all conflicts were resolved by a third reviewer. Full-text articles were then reviewed and assessed against the inclusion criteria by one reviewer then verified by the second reviewer. Uncertainties at this stage were resolved through discussions with the research team. The reference lists of included full-text articles were then reviewed to identify other potential studies for inclusion.

Data Charting & Data Items

For peer review only

Data was extracted and mapped to four categories: Descriptive details of the study (e.g., authors and year, country, sample characteristics, study design, decision-making level, and SRMNCAH priority), characteristics of KT strategy of activity, KT strategy details, mode of KT delivery, stakeholder involvement), outcomes and direction of effect, and identified barriers and enablers identified by study authors. Study data were extracted using the data extraction tool by one reviewer and was verified by another to ensure all relevant data were captured. Critical appraisal of individual sources of evidence were not conducted.

Synthesis of Results

Following data extraction, the income level of each country identified from the studies was determined using the World Bank classification.[19] Study details were grouped into four categories that aligned with each of the research questions: KT strategy, stakeholder engagement, reported outcomes, and types of barriers and enablers identified. Quantitative summaries and thematic analysis were then applied to each grouping to identify potential trends across country income levels and SRMNCAH thematic areas. Study data were also mapped on to the PMNCH's high-level outcomes of interest identified in the 2021-2025 Strategy (e.g., policy, service delivery, financing)[10] and narrative summaries were produced.

To facilitate summarizing content associated with the KT strategies, details of each KT strategy were mapped to the Behaviour Change Wheel (BCW).[20] The BCW provides a synthesis of 19 behaviour change theories in a comprehensive, theory-based tool that can be used to identify important behaviour change elements to consider in intervention design.[21] The BCW includes nine intervention functions that can be used to guide intervention content and design. It also includes seven policy categories to guide implementation of behaviour change interventions and policies. [20] KT strategies were mapped to relevant BCW intervention functions and policy categories by two independent researchers. Researchers met to review BCW coding and discrepancies were resolved through discussion.

For peer review only

239 **Patient and Public Involvement**

240 This scoping review was conducted without the involvement of patients or members of the
241 public.

242 **RESULTS**

243 **Selection of Sources of Evidence**

244 The search strategy returned 11,190 studies for screening. After removing 3,626
245 duplicates, 7,564 titles and abstracts were screened by reviewers. This stage identified 212
246 full-text studies to review. Following full-text analysis, 154 studies were excluded, resulting
247 in 58 studies included in the review. A review of the reference lists of included studies
248 identified 26 additional studies that met the inclusion criteria. The grey literature search
249 identified one study for inclusion; five additional studies were included from screening the
250 reference list of a relevant systematic review. This resulted in a final total of 90 included
251 studies in the scoping review. The selection process and sources of evidence are summarized
252 in a PRISMA-ScR flow diagram (Figure 1).[22]

253 * Insert Figure 1*

254 **Characteristics of Sources of Evidence**

255 A summary of the characteristics of the 90 included studies can be found in Table 1, with
256 a comprehensive description of studies found in Supplementary File 2. All studies were
257 published between 2000-2020, with an increase in relevant publications since 2006. Just over
258 a third (34%) where published between 2011-2015[23–53] and another 34% between 2016-
259 2020.[54–84] Thirty-two percent of studies were quasi-experimental
260 designs,[23,25,26,28,31,32,34,35,44,50,51,59–61,67,68,75,76,83,85–92] with observational
261 (22%),[36,37,39,43,45,52,71,72,77,80,93–102] mixed methods (17%),[40–42,47,53,56–58,64,69,82,103–106]

experimental (16%),[24,27,38,62,66,74,78,79,107–112] and qualitative (11%)[29,30,33,49,54,55,63,70,81,113] designs also identified. Two editorials were also included.[65,73]

Country Income Levels

The majority of the studies (52%) were conducted in high-income countries,[23–25,27–29,31,33–35,38,40,41,43–45,49,53,56,57,62,66–68,71,73,74,74,80,83,87,93–95,97,98,100,102–105,107,108,108,109,112] including Canada, the United States, and Australia. Middle-income countries were the setting for 36% studies,[26,30,32,36,39,42,46–48,50,52,63–65,75–77,81,82,85,86,88–92,96,101,110,111,113] including Nepal, Egypt, and Zambia, with most middle-income countries considered low-middle income (81%) and 19% considered upper-middle income. Only 4% of included studies were located in low-income countries, such as Uganda and Madagascar [58–60,99]. Seven countries (i.e., Bangladesh, Cameroon, Ethiopia, Myanmar, Nigeria, Solomon Islands and Uganda) across 14% of studies represented humanitarian or highly fragile settings,[37,42,54,58,59,61,70,72,85,99] according to Organisation for Economic Co-operation and Development.[114] Eight percent of studies included multi-income countries, with six targeting both low- and middle-income countries (e.g., Uganda and Peru), [37,54,61,69,70,72] and one targeting middle and high-income countries (e.g., Brazil and Chile).[106] As results were reported by country in these seven studies, we analyzed these studies at each of the appropriate income levels.

SRMNCAH priorities

Child health and wellbeing was the most commonly identified priority, with 30% targeting these concerns.[27,29,32,33,35,36,38,40,43,46,47,51,57,62,67,74,78–80,83,85,89,91,94,98–100,105,108,110] Twenty-eight percent addressed newborn health and wellbeing or stillbirths, [23,25,26,28,31,34,50,59,60,60,64,68,81,82,86,88,90,95–97,109,111] and a quarter of studies addressed maternal health and wellbeing.[24,37,39,44,45,52,54,55,58,63,72,73,75–77,87,92,93,103,104,112,113] Studies addressing sexual and reproductive health and rights (SRHR [12%]) [30,38,42,61,65,66,69–71,101,106] and

adolescent health and wellbeing (4%)[33,41,56,107] priorities were identified, but in a lower volume compared to studies addressing maternal, child and newborn or stillbirth priorities. Some studies (13%) also included multiple SRMNCAH priorities, with eight targeting both maternal and newborn health and wellbeing,[37,46,54,81,82,84,90,92] two targeting maternal and child health and wellbeing,[52,72] and two targeting maternal, child and newborn health and wellbeing.[75,76] These studies were analyzed at each of the appropriate priority levels.

PMNCH High-Level Outcomes (2021-2025)

Across the priority high-level outcomes of the PMNCH 2021-2025 Strategy, the majority of included studies (81%) addressed *service delivery*. *Policymaking* was also commonly identified (19%). None of the included KT strategies addressed *financing* as a central focus.

Settings

KT strategies were primarily implemented in hospitals (39%), community (25%) or primary care (8%) settings. Three percent of strategies were conducted within government health departments or agencies.[71,73,108] There were rarely enough details provided to explore specific departmental settings in hospitals, although four hospital studies were conducted in newborn and pediatric intensive care units (NICUs/PICUs),[26,31,89,109] one in a labour and delivery department,[93] and one specified the hospital was in a rural setting.[86] Community settings were also described in limited detail, however eight studies were conducted in childcare centres,[27,35,38,62,67,74,79,83] two in schools,[78,107] and one in a mental health community clinic.[56]

Publication Date	Country by Income Level	Study Design	SRMNCAH Priorities	Examples of SRMNCAH Health Topics	Settings	PMNCH Function of Interest
**2000-2005:** n=8 (9%)<br>**2006-2010:** n=20 (22 %)<br>**2011-2015:** n=31 (34 %)<br>**2016-2020:** n=31 (34 %)	**Low:** n= 4 (4%)<br>**Low& Middle:** n= 6 (7%)<br>**Lower middle:** n= 26 (81%)<br>**Upper middle**: n=6 (19%)<br><br>**Middle & High:** n= 1 (1%)<br>**High:** n= 47 (52%)	**Experimental:** n=14 (16 %)<br>**Quasi-experimental:** n= 29 (32%)<br>**Observational:** n= 20 (22%)<br>**Qualitative:** n= 10 (11%)<br>**Mixed methods**: n= 15 (17%)<br>**Editorials:** n= 2 (2%)	**Adolescent**: n=4 (4%)<br>**Child:** n=28 (31%)<br>**Maternal:** n=14 (16%)<br>**Newborn or stillbirths:** n=20 (22%)<br>**Maternal & newborn:** n=8 (9%)<br>**Maternal & child**: n=2 (2%)<br>**Maternal & child & newborn:** n=2 (2%)<br>**SRHR:** n=12 (13%)	**Adolescent:** Substance use[107]<br>**Child:** Child nutrition[27,35,38,67,74,78,79]<br>**Maternal:** Postpartum depression,[45] eclampsia and pre-eclampsia[113]<br>**Newborn or stillbirths:** Newborn sleep,[23] newborn vaccination[97]<br>**SRHR:** Family planning[42,49,65,69,70,101]	**Hospitals:** n= 35 (39%)<br>**Community:** n= 23 (25%)<br>**Childcare centres or schools:** n=10 (11%)<br>**Primary Care:** n=7 (8%)<br>**Government departments:** n=3 (3%)	**Financing:** n=0<br>**Policymaking:** n= 17 (19%)<br>**Service delivery:** n= 73 (81%)

Table 1. Overview of characteristics of included studies (n=90)

For peer review only

Synthesis of KT Strategies and Activities:

BCW Intervention Functions

Mapping the KT strategies to the BCW intervention functions identified that all but one study contained at least one intervention function. All nine BCW intervention functions were identified across the KT strategies, *with Education, Training,* and *Environmental Restructuring* the most common (Table 2).

BCW Intervention Functions		
**Intervention Function and Definition** [20]	**Intervention Function Stratified by Country Income Level***	**Intervention Function Stratified by SRMNCAH priority****
**Education (n=73; 81%)** (Increasing knowledge or understanding)	Low: n= 8 (80%)<br>Middle: n= 30 (77%)<br>High: n= 45 (88%)	Adolescent: n=3 (75%)<br>Child: n=29 (91%)<br>Maternal: n=21 (78%)<br>Newborn or stillbirths: n=23 (77%)<br>SRHR: n= 9 (75%)
**Training (n=46; 51%)** (Imparting skills)	Low: n= 6 (60%)<br>Middle: n= 19 (41%)<br>High: n= 26 (52%)	Adolescent: n= 2 (50%)<br>Child: n= 14 (44%)<br>Maternal: n= 12 (46%)<br>Newborn or stillbirths: n= 16 (53%)<br>SRHR: n= 7 (58%)
**Environmental Restructuring (n=45; 50%)** (Changing the physical or social context)	Low: n= 4 (40%)<br>Middle: n= 16 (41%)<br>High: n= 30 (58%)	Adolescent: n= 1 (25%)<br>Child: n= 22 (69%)<br>Maternal: n= 10 (38%)<br>Newborn or stillbirths: n= 14 (47%)<br>SRHR: n= 6 (50%)
**Enablement (n=22; 24%)** (Increasing means/reducing barriers)	Low: n= 5 (50%)<br>Middle: n= 6 (15%)<br>High: n= 14 (29%)	Adolescent: n= 1 (25%)<br>Child: n= 8 (25%)<br>Maternal: n= 6 (23%)<br>Newborn or stillbirths: n= 4 (13%)<br>SRHR: n= 2 (13%)
**Persuasion (n=11; 12%)** (communication used to induce positive or negative feelings or stimulate action)	Low: n= 1 (10%)<br>Middle: n= 2 (5%)<br>High: n= 10 (21%)	Adolescent: n= 0<br>Child: n= 8 (25%)<br>Maternal: n=3 (12%)<br>Newborn or stillbirths: n= 1 (3%)<br>SRHR: n=1 (8%)
**Modelling (n=7; 8%)** (Providing an example for people to aspire to or imitate)	Low: n= 0<br>Middle: n= 3 (8%)<br>High: n= 4 (8%)	Adolescent: n= 0<br>Child: n= 2 (6%)<br>Maternal: n= 2 (8%)<br>Newborn or stillbirths: n= 2 (7%)<br>SRHR: n= 1 (8%)
**Incentivisation (n=5; 6%)** (Creating expectation of reward)	Low: n= 0<br>Middle: n= 1 (3%)<br>High: n= 4 (8%)	Adolescent: n= 0<br>Child: n= 4 (13%)<br>Maternal: n= 0<br>Newborn or stillbirths: n= 0<br>SRHR: n= 1 (8%)

For peer review only

**Coercion (n=2; 2%)**<br>(Creating expectation of punishment or cost)	Low: n= 1 (10%)<br>Middle: n= 1 (3%)<br>High: n=0	Adolescent: n= 0<br>Child: n= 0<br>Maternal: n= 1 (4%)<br>Newborn or stillbirths: n= 2 (7%)<br>SRHR: n=0
**Restriction (n=1; 1%)**<br>(Using rules to reduce the opportunity to engage in the target behaviour)	Low: n=0<br>Middle: n= 1 (3%)<br>High: n=0	Adolescent: n= 0<br>Child: n=0<br>Maternal: n= 0<br>Newborn or stillbirths: n=1 (3%)<br>SRHR: n= 0
**BCW Policy Categories**		
**Policy Category and Definition [20]**	**Policy Category Stratified Country Income Level***	**Policy Category Stratified by SRMNCAH priority****
**Guidelines (n=25; 28%)**<br>(Creating documents that recommend or mandate practice)	Low: n= 5 (50%)<br>Middle: n= 12 (31%)<br>High: n= 12 (25%)	Adolescent: n= 1 (25%)<br>Child: n=6 (19%)<br>Maternal: n= 9 (35%)<br>Newborn or stillbirths: n=8 (27%)<br>SRHR: n= 5 (42%)
**Service Provision (n=18; 20%)**<br>(Delivering a service)	Low: n= 0<br>Middle: n= 2 (5%)<br>High: n= 15 (31%)	Adolescent: n= 1 (25%)<br>Child: n=6 (19%)<br>Maternal: n= 4 (15%)<br>Newborn or stillbirths: n=7 (23%)<br>SRHR: n= 3 (25%)
**Communication/Marketing (n=12; 13%)**<br>(Using print, electronic, telephonic or broadcast media)	Low: n= 2 (20%)<br>Middle: n=4 (10%)<br>High: n= 8 (17%)	Adolescent: n= 0<br>Child: n=5 (16%)<br>Maternal: n= 3 (12%)<br>Newborn or stillbirths: n= (13%)<br>SRHR: n= 3 (25%)
**Environmental/Social Planning (n=11; 12%)**<br>(Designing and/or controlling the physical or social environment)	Low: n= 1 (10%)<br>Middle: n= 4 (10%)<br>High: n= 6 (12%)	Adolescent: n= 0<br>Child: n=4 (13%)<br>Maternal: n= 5 (19%)<br>Newborn or stillbirths: n=5 (17%)<br>SRHR: n=0
**Regulation (n=7; 8%)**<br>(Establishing rules or principles of behaviour or practice)	Low: n= 1 (10%)<br>Middle: n= 2 (5%)<br>High: n= 5 (10%)	Adolescent: n= 0<br>Child: n=3 (9%)<br>Maternal: n= 1 (3%)<br>Newborn or stillbirths: n=4 (13%)<br>SRHR: n=0
**Legislation (n=3; 3%)**<br>(Making or changing laws)	Low: n= 0<br>Middle: n= 3(8%)<br>High: n= 0	Adolescent: n= 0<br>Child: n=0<br>Maternal: n= 1 (4%)<br>Newborn or stillbirths: n=0<br>SRHR: n=2 (17%)

Table 2. Summary of BCW intervention functions and policy categories identified in KT strategies by country income level and SRMNCAH priority* please note country income levels include seven multi-country studies (n=97); ** please note priorities include 12 multi-

For peer review only

priority studies (n=104)

Education was the most commonly identified element from the included studies, identified in 81% of studies. Types of *Education* provided included modules, staff workshops, slides and other resources. This function was identified in 73 KT strategies across country income levels (88% in high-income countries, 80% of low-income countries, and 77% of middle-income countries). At the SRMNCAH priority level, 91% of child health and wellbeing strategy included an *Education* function. *Education* was also identified in 81% of the maternal health interventions, as well as 75% of each of the SRHR, and adolescent and wellbeing interventions.

Training was identified in 51% of strategies. *Training* typically included conveying skills to staff members and healthcare providers. This element was included in 60% of the KT strategies aimed at low-income countries, 52% of high-income countries and 41% of middle-income countries. Strategies stratified by SRMNCAH priority identified *Training* was utilized consistently across each priority.

Environmental Restructuring was identified in 50% of the KT strategies. This element included reorganizing how services were provided, how health centers were set up, and adding additional resources (e.g., tools, team members) in the health centre or health system to facilitate use of the KT strategy. This element was identified at all three income levels but was more likely to be used in high-income countries (58%), compared to middle (41%) and low-income (40%) countries. Sixty-nine percent of child health and wellbeing strategies included this function, as well as 50% of the sexual and reproductive health and rights strategies. *Environmental Restructuring* was only applied in one of the four (25%) adolescent health and wellbeing strategies.

BCW Policy Categories

Sixty percent of studies included policy category content, with six of the seven BCW policy categories were identified across the strategies (Table 2). No strategies applied content related to the *Fiscal* category option. *Guidelines* and *Service Provision* were the most commonly identified, with G*uidelines* identified in 28% of KT strategies and *Service Provision* identified in 20% of studies. *Guidelines* were implemented in 50% of the low-income countries, compared to only 31% of middle-income and 25% of the high-income countries. *Guidelines* were most frequently used in 50% of KT strategies related to sexual or reproductive health and rights, and 36% of maternal health and well-being strategies.

Service Provision was implemented in 89% of the high-income countries, compared to only 5% of middle-income countries and none of the low-income countries. This policy category was divided across SRMNCAH priorities. SRHR included *Service Provision* in 25% of strategies, and 25% of strategies targeting adolescent health and wellbeing included this category.

Mode of KT Delivery

Seventy-two percent of KT strategies identified the mode of KT delivery, with the majority using one mode of delivery, while 27% used two or more modes. The majority of these studies used in-person delivery (59%) as the sole mode of delivery. Of the multi-modal delivery strategies, in-person delivery was also included as a mode in all but one study.[111] High-income countries used more multi-mode interventions, such as in-person with additional online or web-based components, compared to those in low- and middle-income countries. Studies published between 2018-2020 have used evolving trends in technology, such as webinars and social media,[73,83] as modes of delivery.

Synthesis of Stakeholder Involvement

368 There was an overall lack of description provided on how stakeholders were involved
369 in designing or implementing KT strategies, with 31% of studies not providing any
370 description of stakeholder engagement. Of the remaining studies that did provide details, the
371 level of detail varied by article, with some simply acknowledging stakeholders were engaged,
372 while others provided a more comprehensive view of the stakeholder groups involved and
373 their roles. Commonly identified stakeholder groups included: government and policy
374 makers, healthcare providers, civil society organizations, members of the public, and
375 members of the research community (Table 3). Engagement with these groups was
376 distributed across country income level, with two notable exceptions. First, civil society
377 organizations were more likely to be engaged in low (60%) and middle-income (48%)
378 countries compared to high-income (27%) countries. Additionally, government and
379 policymakers were engaged by low (50%) and middle-income (62%) countries much more
380 often than in high-income (8%) countries.

381 Stakeholder engagement was dispersed across the SRMNCAH priorities. Priorities
382 addressing SRHR were more likely to include policymakers and government as well as civil
383 society organizations compared to other priorities. Half of strategies addressing adolescent
384 health and wellbeing, while 25% of SRHR engaged healthcare providers including clinicians,
385 nurses and allied healthcare professionals. Involvement of researcher communities was
386 identified across all six priorities but was rarely used in newborn health and wellbeing or
387 stillbirth strategies (7%).

**Type of Stakeholders involved in KT Strategy**	**Stakeholders by Country Income Level***	**Stakeholders by SRMNCAH Priority****
**Policymakers and Government**	Low: n= 5 (50%)<br>Middle: n= 24 (62%)<br>High: n= 4 (8%)	Adolescent: n= 1 (25%)<br>Child: n= 4 (13%)<br>Maternal: n= 9 (35%)<br>Newborn or stillbirths: n= 3 (10%)<br>SRHR: n= 7 (63%)
**Healthcare Providers**	Low: n= 5 (50%)	Adolescent: n= 2 (50%)

For peer review only

**and Administrators**	Middle: n= 17 (44%)<br>High: n= 23 (48%)	Child: n= 10 (31%)<br>Maternal: n= 11 (42%)<br>Newborn or stillbirths: n= 9 (30%)<br>SRHR: n= 3 (25%)
**Civil Society Organizations**	Low: n= 6 (60%)<br>Middle: n= 19 (48%)<br>High: n= 13 (27%)	Adolescent: n= 1 (25%)<br>Child: n= 4 (13%)<br>Maternal: n= 3 (13%)<br>Newborn or stillbirths: n= 2 (7%)<br>SRHR: n= 7 (63%)
**Public**	Low: n= 2 (20%)<br>Middle: n= 1 (3%)<br>High: n= 10 (21%)	Adolescent: n= 2 (50%)<br>Child: n= 3 (9%)<br>Maternal: n= 1 (4%)<br>Newborn or stillbirths: n= 2 (7%)<br>SRHR: n= 2 (17%)
**Research Community**	Low: n= 3 (30%)<br>Middle: n= 10 (26%)<br>High: n= 17 (35%)	Adolescent: n= 1 (25%)<br>Child: n= 9 (28%)<br>Maternal: n= 10 (38%)<br>Newborn or stillbirths: n= 2 (7%)<br>SRHR: n= 3 (25%)

Table 3. Summary of stakeholder engagement by country income level and SRMNCAH priority; * please note country income levels include seven multi-country studies (n=97); ** please note priorities include 12 multi-priority studies (n=104)

For peer review only

Synthesis of Outcomes

Nearly 80% of KT strategy outcomes were reported at only a single outcome level (e.g., patient or healthcare provider), with 20% studies reporting multiple outcome levels (e.g., healthcare provider and system). At the single outcome level, 38% of outcomes were measured at the healthcare provider level (e.g., increased knowledge) and 29% at system level (e.g., reductions in safety incidents). Only 5% of strategies included patient level outcomes (e.g., improved newborn sleep). Patient outcomes were more likely to be included in multi-level outcomes, along with healthcare provider and system level (e.g., immunization rates, quality of care provided and healthcare system use) outcomes. Of the multi-level outcome studies, 17% had outcomes at two levels (e.g., patient and healthcare providers, healthcare providers and system, or patient and system) and 4% of studies included three level outcomes (e.g., patient, healthcare provider and system).

Healthcare provider outcomes were mostly reported in high-income (44%) and low-income (40%) countries, compared to 26% of middle-income countries, while system level outcomes were more common in low-income (40%) and middle-income (41%) countries. Healthcare provider outcomes were identified across the SRMNCAH priorities, ranging from 23-75% for maternal health and wellbeing strategies to those for adolescent wellbeing, respectively. While fewer maternal health and wellbeing strategies included healthcare provider level outcomes, nearly half of these strategies (48%) were aimed at system level outcomes. Childhood health and wellbeing strategies were also most likely to address multi-level outcomes compared to all other priorities.

For peer review only

Synthesis of Barriers and Enablers

Few studies reported barriers and enablers to using KT strategies to promote the use of evidence in SRMNCAH and wellbeing. Fewer than half of the studies (43%) outlined barriers and even fewer identified enablers (40%) to their KT strategies. When studies included barriers and enablers, the level of detail provided varied across the studies, with some studies providing a brief list of these factors while other studies provided more detailed descriptions and how each affected the KT strategy. A summary of identified barriers and enablers can be found in Table 4.

**Barriers Identified Across Studies (n=39)**		
	**Stratified Country Income Level***	**Stratified by SRMNCAH Priority****
**Limited Resources** (n=22)	Low: n= 4 (40%)<br>Middle: n= 9 (23%)	Adolescent: n=2 (50%)<br>Child: n= 6 (19%)

	High: n= 9 (19%)	Maternal: n= 6 (23%)<br>Newborn or stillbirths: n= 2 (7%)<br>SRHR: n= 6 (50%)
**Time Constraints** (n=12)	Low: n= 0<br>Middle: n=2 (5%)<br>High: n=10 (21%)	Adolescent: n= 1 (25%)<br>Child: n= 5 (16%)<br>Maternal: n= 2 (8%)<br>Newborn or stillbirths: n= 2 (7%)<br>SRHR: n= 2 (13%)
**Negative Attitudes** (n=10)	Low: n= 3 (30%)<br>Middle: n=3 (8%)<br>High: n=5 (10%)	Adolescent: n= 1 (25%)<br>Child: n= 2 (6%)<br>Maternal: n=3 (12%)<br>Newborn or stillbirths: n= 1 (3%)<br>SRHR: n= 3 (25%)
**Lack of Knowledge** (n=9)	Low: n= 1 (10%)<br>Middle: n=3 (8%)<br>High: n=5 (10%)	Adolescent: n=1 (25%)<br>Child: n= 2 (6%)<br>Maternal: n=4 (15%)<br>Newborn or stillbirths: n= 3 (10%)<br>SRHR: n=1 (8%)
**Lack of Training** (n=7)	Low: n= 0<br>Middle: n=0<br>High: n=7 (15%)	Adolescent: n= 1 (25%)<br>Child: n= 4 (13%)<br>Maternal: n= 2 (8%)<br>Newborn or stillbirths: n= 0<br>SRHR: n=0
**Poor Engagement with Stakeholders** (n=7)	Low: n= 0<br>Middle: n=4<br>High: n=3	Adolescent: n= 1 (25%)<br>Child: n= 3 (9%)<br>Maternal: n= 3 (12%)<br>Newborn or stillbirths: n= 1 (3%)<br>SRHR: n=1 (8%)
**Enablers Identified Across Studies (n=36)**		
	**Stratified Country Income Level***	**Stratified by SRMNCAH Priority****
**Supportive Stakeholder Involvement** (n=24)	Low: n= 4 (40%)<br>Middle: n= 10 (26%)<br>High: n= 10 (21%)	Adolescent: n= 2 (50%)<br>Child: n= 3 (9%)<br>Maternal: n=7 (27%)<br>Newborn or stillbirths: n= 4 (13%)<br>SRHR: n=8 (67%)
**Access to Resources** (n=8)	Low: n= 2 (20%)<br>Middle: n=2 (5%)<br>High: n=4 (8%)	Adolescent: n= 0<br>Child: n= 1 (3%)<br>Maternal: n=3 (12%)<br>Newborn or stillbirths: n= 3 (10%)<br>SRHR: n= 2 (17%)
**Access to Knowledge** (n=8)	Low: n= 4 (40%)<br>Middle: n=4 (10%)<br>High: n=0	Adolescent: n= 0<br>Child: n= 2 (6%)<br>Maternal: n= 3 (12%)<br>Newborn or stillbirths: n= 2 (7%)<br>SRHR: n= 3 (25%)
**Positive Attitudes or Empowerment** (n=7)	Low: n= 2 (20%)<br>Middle: n=3 (8%)<br>High: n=2 (4%)	Adolescent: n= 0<br>Child: n= 2 (6%)<br>Maternal: n=4 (15%)

For peer review only

		Newborn or stillbirths: n= 1 (3%)<br>SRHR: n=0
**Skills and Training** (n=6)	Low: n= 0<br>Middle: n=0<br>High: n=6 (13%)	Adolescent: n=0<br>Child: n= 3 (9%)<br>Maternal: n=2 (8%)<br>Newborn or stillbirths: n= 0<br>SRHR: n=1 (8%)

Table 4. Summary of barriers and enablers identified across strategies by country income level and SRMNCAH priority; * please note country income levels include seven multi-country studies (n=97); ** please note priorities include 12 multi-priority studies (n=104)

Identified Barriers: Limited resources was the most commonly reported barrier for using KT strategies across countries of all income levels and SRMNCAH priorities. In 56% of strategies, limited resources referred to physical (e.g., funding) or human resources (e.g., healthcare staff) constraints. Low-income countries were more likely to report limited resources (40%) compared to high-income countries (19%). In terms of SRMNCAH priority, 50% of SRHR strategies identified limited resources as barriers to using KT strategies.

Secondly, time constraints were reported in 21% of high-income countries. This type of barrier delayed the implementation process, including fidelity of KT strategies. Lastly, negative attitudes was the third commonly reported barrier, reported by 30% of low-income countries. Examples of negative attitudes included resistance to change, lack of confidence, and poor 'buy-in' for using KT strategies.

Identified Enablers: Supportive stakeholder involvement was the most commonly identified enabler to KT strategies. Two-thirds of studies which reported enablers identified the importance of developing supportive relationships with stakeholders, and that the partnerships forged supported the implementation of the KT strategy. Supportive stakeholder involvement included successful collaboration and partnerships with, but not limited to, healthcare providers, government bodies, or non-profit organizations. This enabler was identified across all country income levels but was most common in low-income countries

For peer review only

(40%). In terms of SRMNCAH priority, 67% of SRHR studies reported supportive stakeholder involvement as enablers.

Secondly, access to resources and knowledge were the next commonly reported enablers for using KT strategies. Resources in these articles included financial as well as human resources. This enabler was more common in low-income countries (20%) compared to middle-income (8%) or high-income (5%) countries. This enabler was identified across the SRMNCAH priorities, with the exception of adolescents' health and well-being. Finally, only high-income countries reported skills and training as enablers for using KT strategies.

DISCUSSION

This rapid scoping review identified 90 studies published since 2000 which utilized KT strategies and policies to support the use of evidence for improving SRMNCAH. While a wide range of studies across country income levels and SRMNCAH priorities were identified, most KT strategies were implemented in high-income countries and focused on maternal, newborn or stillbirths, or child health and wellbeing topics. The review identified key gaps in KT interventions to support evidence-based decision-making for SRHR, adolescent health and wellbeing and SRMNCAH financing. Our findings illustrate the majority of KT strategies included an *education* component and strategies were commonly aimed at addressing healthcare provider and system-level level outcomes. Across PMNCH outcomes of interest, most strategies address *service delivery* or *policymaking,* with none addressing SRMNCAH financing. While few details were typically provided on stakeholder engagement, or on barriers and enablers in the KT process, it was noted that collaboration with stakeholders and building partnerships with local actors, such as government or health authorities, facilitated use of KT strategies.

Low- and middle-income countries identified a lack of resources (e.g., funding, staff, physical resources) as the most common barrier to implementing and sustaining KT

For peer review only

strategies. While lack of resources were also identified in high-income countries, these barriers look different based on country income level and are more often pronounced in low- and middle-income countries.[113] As we only identified ten KT strategies implemented or evaluated in low-income countries, this may suggest more work is needed in these countries to move evidence into practice to improve SRMNCAH outcomes while addressing the economic, resource, and health system barriers they experience.[115] Using a tailored approach to specifically address the unique barriers in low-income countries may help support the successful implementation of KT strategies and improve maternal, child and adolescent health outcomes within these countries.[115]

Engaging with stakeholders was viewed as a key enabler to KT strategies included in this review. Among the groups of stakeholders involved, low- and middle-income countries were more likely to include members of civil societies and non-governmental organizations compared to high-income countries. Additionally, government and policymakers were engaged by low- and middle-income countries much more often than in high-income countries. Involving civil societies in knowledge translation, especially among low-income countries, is crucial as these organizations often have the capacity and resources required to implement recommendations.[116] Drawing on civil society stakeholder groups throughout the design and implementation of KT strategies may provide vital support to help facilitate the implementation and evaluation of KT strategies.

SRMNCAH and Wellbeing

Maternal health and wellbeing strategies addressed pre-term labour management, labour and delivery outcomes and perinatal care, although primarily in high-income countries.[63,84,102,103,112] These strategies typically included utilizing clinical guidelines and education for nurses to help support improvements in these maternal outcomes. Our review

identified a critical gap around effective KT approaches to support evidence-based SRMNCAH interventions in low-income countries. Yet, these women and communities experience more barriers to high quality healthcare, including trained healthcare providers during childbirth.[117,118] More investments and capacity strengthening efforts are needed to support KT interventions in low-income settings, thus advancing evidence-based policy and practice in settings where the needs are the most dire. This includes humanitarian and fragile settings, which bear a disproportionate burden of poor SRMNCAH outcomes. For instance, maternal mortality increases by 11% on average in conflict zones and by 28% in the worst-hit areas.[119] Additionally, more than 10 million deaths in children younger than 5 years can be attributed to conflict between 1995 and 2015 globally.[119] In addition, up to a third of girls living in a humanitarian setting report that their first sexual encounter was forced.[120]

Many of the identified strategies were aimed at improving newborn, childhood and maternal mortality rates, particularly in low and middle-income countries.[23,37,52,59,72,81,86,90] Strategies included education sessions targeting healthcare providers to ensure quality care is provided to support health outcomes among these groups, and one strategy outlined an advocacy campaign targeting government members to support the provision of free health services for pregnant women and children to help reduce mortality rates.[52] Providing educational and training opportunities to healthcare providers is vital to targeting health system barriers often experienced in low- and middle-income countries.[118,121] However it is recommended to include stakeholder groups within and beyond the health system in these strategies, and these multi-sectoral stakeholder strategies will need to be sustainable to ensure continuous access to high quality healthcare for children.

Our review identified an overall lack of relevant KT strategies addressing adolescent health and wellbeing. This is a significant gap in the literature as adolescents represent over

1.2 billion of today's population, and 90% of these youth living in low and middle-income countries.[122,123] While the adolescent studies included in this review addressed issues including substance use, mental health and overall health needs,[41,53,56,107] there is a lack of research into supporting dietary and lifestyle choices and SRHR in this population. Focusing on developing KT programs and policies to address the health needs of adolescents is essential to supporting the transition into healthy adulthood. Future KT programs and policies should be co-designed with adolescents and youth to harness their capacity and advocacy skills and to ensure their unique health needs are being addressed.[123]

SRMNCAH Financing

Our review identified a key gap in effective KT interventions to support evidence-based decision-making on SRMNCAH financing. This finding highlights the need for greater efforts to ensure that robust health financing evidence is used to strengthen public financial management systems pertaining to SRMNCAH.[124] This is critical to support efficient spending in times of COVID-19, whereby governments are struggling with shrinking fiscal spaces and major disruptions to essential SRMNCAH services.[125]

Implications for Future KT Strategies

Findings from this scoping review identified educational interventions aimed at changing healthcare provider behaviours to improve provision of care are implemented across all countries and SRMNCAH priorities. Future work should build on these good practices to address issues around SRHR, adolescent health and wellbeing, and SRMNCAH financing. It will also be essential for teams designing and implementing KT strategies to integrate stakeholder groups early on in the process and co-design KT interventions to optimize the success of work.[126] Depending on the complexity and scalability of the KT strategies,

547 especially in low and middle-income countries, there is an opportunity to identify and address
548 barriers to optimal implementation. Utilizing models, such as the Ottawa Model for Research
549 Use recommended by Santesso & Tugwell, throughout the KT process may prove useful to
550 support effective implementation of KT strategies and positive outcomes.[115,127]

551 Across the studies identified in our review, only 16% utilized an experimental design,
552 with most studies being observational in nature, thus impeding the assessment of the
553 effectiveness of KT interventions. Future research could benefit from more experimental
554 study designs (e.g., cluster randomized control trials, interrupted time series, controlled
555 before and after studies) – and eventually a systematic review and meta-analysis - to evaluate
556 the effectiveness of SRMNCAH strategies, which could provide useful direction and
557 guidance for KT decision makers and policymakers.

558 ***Limitations***

559 Due to the rapid nature of this scoping review, it is possible we may have missed relevant
560 KT strategies in the search. Another potential limitation is only identifying one piece of grey
561 literature in the scoping review, which may have been due to our search strategy and strict
562 inclusion criteria. However, our comprehensive literature and grey literature searches
563 identified a broad range of relevant strategies across countries and SRMNCAH priorities. The
564 challenges of identifying relevant KT strategies and policies may also be due to how varied
565 the terminology is around knowledge translation (e.g., knowledge transfer, knowledge
566 exchange, knowledge mobilization) and how it is applied across different countries and
567 health areas.[128] Additionally, while few KT strategies included in the review were
568 implemented and evaluated in low-income countries, this may not reflect all of the KT
569 strategies being implementing across these settings. Although we conducted a search of the
570 grey literature, it is possible publication bias may have impacted our ability to include all

unpublished KT work conducted to address SRMNCAH priorities in different settings.[129] Finally, findings from the review are up to date as of July 2020 and it is possible additional relevant strategies may have been published between running the search strategy and report writing. However, this review provides a comprehensive view of KT strategies which have been published over the past 20 years to address the health and wellbeing of women, children and adolescents.

CONCLUSION

Most KT strategies included in this scoping review were implemented in high-income countries and aimed at maternal, newborn and child health and wellbeing. We identified a critical gap in the published literature of KT approaches in low-income countries and humanitarian and fragile settings. Meaningful engagement of stakeholders in KT was identified as a key enabler to enhance people-centered and gender-responsive SRMNCAH policy, service delivery and financing. Effective KT approaches are required to support the implementation and impact of multi-sectoral policies and interventions. As SRMNCAH outcomes worsen as a consequence of the COVID-19 pandemic, it is critical to support SRMNCAH interventions and protect the progress made to date. Knowledge translation approaches have a key role to play to ensure that strategies to mitigate the disruptions to SRMNCAH services are effective, feasible and acceptable, while addressing the equity gap and ensuring that vulnerable communities are not left behind.

ABBREVIATIONS:

BCW: Behaviour Change Wheel

KT: Knowledge Translation

NICU: neonatal intensive care unit

PICU: pediatric intensive care unit

PMNCH: Partnership for Maternal, Newborn & Child Health

PRESS: Peer Review of Electronic Search Strategy

PRISMA-ScR: Preferred Reporting Items for Systematic Reviews and Meta-Analyses - Extension for Scoping Reviews

SPOR: Strategy for Patient-Oriented Research

SRHR: Sexual and Reproductive Health and Rights

SRMNCAH: Sexual, Reproductive, Maternal, Newborn, Child, and Adolescent Health

WHO: World Health Organization

For peer review only

CONTRIBUTIONS: EVL, ZB, JAC, HW, RU, LB and LW developed the scoping review protocol. JAC, AJG, HDS, JK and EVL contributed to developing the initial draft of the manuscript. All authors provided feedback and revisions to the manuscript.

FUNDING: This work was funded by the SPOR Evidence Alliance in collaboration with the Partnership for Maternal, Newborn & Child Health (PMNCH) at the World Health Organization (Grant Number: Not Applicable).

COMPETING INTERESTS: The authors have no conflicts to declare.

ETHICS STATEMENT: Not Applicable

DATA SHARING: Data sharing not applicable as no datasets generated and/or analysed for this study.

REFERENCES

1. Victora, C. G. *et al.* Countdown to 2015: a decade of tracking progress for maternal, newborn, and child survival. *Lancet Lond. Engl.* **387**, 2049–2059 (2016).

2. Protect the progress: rise, refocus and recover. 2020 progress report on the Every Woman Every Child Global Strategy for Women's, Children's and Adolescents' Health (2016–2030). Geneva: World Health Organization and the United Nations Children's Fund (UNICEF); 2020. Licence: CC BY-NC-SA 3.0 IGO.
3. Moynihan, R. *et al.* Pandemic impacts on healthcare utilisation: a systematic review. *medRxiv* 2020.10.26.20219352 (2020) doi:10.1101/2020.10.26.20219352.
4. Chmielewska, B. *et al.* Effects of the COVID-19 pandemic on maternal and perinatal outcomes: a systematic review and meta-analysis. *Lancet Glob. Health* **0**, (2021).
5. United Nations Population Fund (UNPFA). Impact of COVID-19 on Family Planning: What we know one year into the pandemic. /resources/impact-covid-19-family-planning-what-we-know-one-year-pandemic (2021).
6. WHO. *Pulse survey on continuity of essential health services during the COVID-19 pandemic: interim report, 27 August 2020*. https://www.who.int/publications-detail-redirect/WHO-2019-nCoV-EHS_continuity-survey-2020.1 (2020).
7. Countdown 2015 Maternal, Newborn & Child Survival – A Decade of Tracking Progress for Maternal, Newborn and Child Survival: The 2015 Report. http://countdown2030.org/2015/2015-final-report.
8. Organisation for Economic Co-operation and Development. The territorial impact of COVID-19: Managing the crisis across levels of government. *OECD* https://www.oecd.org/coronavirus/policy-responses/theterritorial-impact-of-covid-19-managing-the-crisis-across-levels-ofgovernment-d3e314e1/ (2020).
9. Jha, A., Kickbusch, I., Taylor, P., Abbasi, K., & SDGs Working Group. Accelerating achievement of the sustainable development goals. *BMJ* **352**, i409 (2016).
10. The Partnership for Maternal, Newborn and Child Health (PMNCH). *PMNCH 2021-2025 Strategy*.

For peer review only

https://www.who.int/pmnch/knowledge/publications/pmnch_strategic_plan_2021_2025.pdf?ua=1 (2021).

11. Canadian Institute for Health Research. About Knowledge Translation. https://cihr-irsc.gc.ca/e/29418.html#2 (2016).

12. Milat, A. J. & Li, B. Narrative review of frameworks for translating research evidence into policy and practice. *Public Health Res. Pract.* **27**, (2017).

13. Tabak, R. G., Khoong, E. C., Chambers, D. A. & Brownson, R. C. Bridging research and practice: models for dissemination and implementation research. *Am. J. Prev. Med.* **43**, 337–350 (2012).

14. Slaughter, S. E. *et al.* Classification schemes for knowledge translation interventions: a practical resource for researchers. *BMC Med. Res. Methodol.* **17**, 161 (2017).

15. Tricco, A. C. *et al.* PRISMA Extension for Scoping Reviews (PRISMA-ScR): Checklist and Explanation. *Ann. Intern. Med.* **169**, 467 (2018).

16. Peters, M. D. J. *et al.* Guidance for conducting systematic scoping reviews. *Int. J. Evid. Based Healthc.* **13**, 141–146 (2015).

17. McGowan, J. *et al.* PRESS Peer Review of Electronic Search Strategies: 2015 Guideline Statement. *J. Clin. Epidemiol.* **75**, 40–46 (2016).

18. Covidence. Covidence systematic review software, Veritas Health Innovation, Melbourne, Australia. Available at www.covidence.org.

19. The World Bank. World Bank Country and Lending Groups. https://datahelpdesk.worldbank.org/knowledgebase/articles/906519-world-bank-country-and-lending-groups.

20. Michie, S., Atkins, L. & West, R. *The Behaviour Change Wheel: A Guide to Designing Interventions*. (Silverback Publishing, 2014).

For peer review only

For peer review only - http://bmjopen.bmj.com/site/about/guidelines.xhtml

21. Michie, S., van Stralen, M. M. & West, R. The behaviour change wheel: A new method for characterising and designing behaviour change interventions. *Implement. Sci. IS* **6**, 42 (2011).

22. Page, M. J. *et al.* The PRISMA 2020 statement: an updated guideline for reporting systematic reviews. *BMJ* **372**, n71 (2021).

23. Abney-Roberts, S. E. A Successful Quality Improvement Project to Improve Infant Safe Sleep Practice. *J. Obstet. Gynecol. Neonatal Nurs.* **44**, S43 (2015).

24. Agapidaki, E. *et al.* A theory-based educational intervention to pediatricians in order to improve identification and referral of maternal depression: a quasi-experimental study. *Ann. Gen. Psychiatry* **12**, 37 (2013).

25. Ainsworth, R. M., Mog, C. & Summerlin-Long, S. A Comprehensive Newborn Falls Initiative One Year Later. *J. Obstet. Gynecol. Neonatal Nurs.* **43**, S66 (2014).

26. Al-Rafay, S. S. & Al-Sharkawy, S. S. Educational outcomes associated with providing a comprehensive guidelines program about nursing care of preterm neonates receiving total parenteral nutrition. *Clin. Nurs. Res.* **21**, 142–158 (2012).

27. Alkon, A. *et al.* Nutrition and physical activity randomized control trial in child care centers improves knowledge, policies, and children's body mass index. *BMC Public Health* **14**, 215 (2014).

28. Allen, M. & Schafer, D. J. Nurses Improving the Health of Mothers and Infants by Dancing the 10 Steps to Successful Breastfeeding. *J. Obstet. Gynecol. Neonatal Nurs.* **44**, S52–S52 (2015).

29. Anaby, D., Korner-Bitensky, N., Law, M. & Cormier, I. Focus on participation for children and youth with disabilities: Supporting therapy practice through a guided knowledge translation process. *Br. J. Occup. Ther.* **78**, 440–449 (2015).

For peer review only

30. Ansbro, É. M. *et al.* Introduction of Syphilis Point-of-Care Tests, from Pilot Study to National Programme Implementation in Zambia: A Qualitative Study of Healthcare Workers' Perspectives on Testing, Training and Quality Assurance. *PloS One* **10**, e0127728 (2015).

31. Baer, V. L. *et al.* Implementing a program to improve compliance with neonatal intensive care unit transfusion guidelines was accompanied by a reduction in transfusion rate: a pre-post analysis within a multihospital health care system. *Transfusion (Paris)* **51**, 264–269 (2011).

32. Brennan, M. M. *et al.* Paediatric resuscitation for nurses working in Ghana: an educational intervention. *Int. Nurs. Rev.* **60**, 136–143 (2013).

33. Cameron, D., Russell, D. J., Rivard, L., Darrah, J. & Palisano, R. Knowledge brokering in children's rehabilitation organizations: perspectives from administrators. *J. Contin. Educ. Health Prof.* **31**, 28–33 (2011).

34. Farner, R. *et al.* The nurse champion model for advancing newborn screening of critical congenital heart disease. *J. Obstet. Gynecol. Neonatal Nurs. JOGNN* **43**, 497–506 (2014).

35. Finch, M. *et al.* Impact of a population based intervention to increase the adoption of multiple physical activity practices in centre based childcare services: a quasi experimental, effectiveness study. *Int. J. Behav. Nutr. Phys. Act.* **9**, 101 (2012).

36. Goyet, S. *et al.* Knowledge translation: a case study on pneumonia research and clinical guidelines in a low- income country. *Implement. Sci.* **9**, 82 (2014).

37. Hulton, L. *et al.* Using evidence to drive action: a 'revolution in accountability' to implement quality care for better maternal and newborn health in Africa. *Int. J. Gynaecol. Obstet. Off. Organ Int. Fed. Gynaecol. Obstet.* **127**, 96–101 (2014).

For peer review only

38. Jones, C. O. H. *et al.* 'Even if you know everything you can forget': health worker perceptions of mobile phone text-messaging to improve malaria case-management in Kenya. *PloS One* **7**, e38636 (2012).

39. Kapungu, C. T. *et al.* A community-based continuum of care model for the prevention of postpartum hemorrhage in rural Ghana. *Int. J. Gynaecol. Obstet. Off. Organ Int. Fed. Gynaecol. Obstet.* **120**, 156–159 (2013).

40. Lang, J. M., Franks, R. P., Epstein, C., Stover, C. & Oliver, J. A. Statewide dissemination of an evidence-based practice using Breakthrough Series Collaboratives. *Child. Youth Serv. Rev.* **55**, 201–209 (2015).

41. Mello, M. J. *et al.* Translation of alcohol screening and brief intervention guidelines to pediatric trauma centers. *J. Trauma Acute Care Surg.* **75**, S301-307 (2013).

42. Mody, S. K., Ba-Thike, K. & Gaffield, M. E. The World Health Organization-United Nations Population Fund Strategic Partnership Programme's implementation of family planning guidelines and tools in Asia-Pacific countries. *J. Obstet. Gynaecol. Res.* **39**, 825–830 (2013).

43. Schreiber, J., Marchetti, G. F., Racicot, B. & Kaminski, E. The use of a knowledge translation program to increase use of standardized outcome measures in an outpatient pediatric physical therapy clinic: administrative case report. *Phys. Ther.* **95**, 613–629 (2015).

44. Schwartz, R. *et al.* Washington 'Steps' Up: A 10-Step Quality Improvement Initiative to Optimize Breastfeeding Support in Community Health Centers. *J. Hum. Lact. Off. J. Int. Lact. Consult. Assoc.* **31**, 651–659 (2015).

45. Segre, L. S., Brock, R. L., O'Hara, M. W., Gorman, L. L. & Engeldinger, J. Disseminating perinatal depression screening as a public health initiative: a train-the-trainer approach. *Matern. Child Health J.* **15**, 814–821 (2011).

For peer review only

46. Singh, K. *et al.* Impact evaluation of a quality improvement intervention on maternal and child health outcomes in Northern Ghana: early assessment of a national scale-up project. *Int. J. Qual. Health Care* **25**, 477–487 (2013).

47. Sobel, H. L. *et al.* Implementing a national policy for hepatitis B birth dose vaccination in Philippines: Lessons for improved delivery. *Vaccine* **29**, 941–945 (2011).

48. Sundaram, A., Juarez, F., Ahiadeke, C., Bankole, A. & Blades, N. The impact of Ghana's R3M programme on the provision of safe abortions and postabortion care. *Health Policy Plan.* **30**, 1017–1031 (2015).

49. Tarasoff, L. A., Epstein, R., Green, D. C., Anderson, S. & Ross, L. E. Using interactive theatre to help fertility providers better understand sexual and gender minority patients. *Med. Humanit.* **40**, 135–141 (2014).

50. Twum-Danso, N. A. *et al.* Using quality improvement methods to test and scale up a new national policy on early post-natal care in Ghana. *Health Policy Plan.* **29**, 622–632 (2014).

51. Wolfe, A. D. *et al.* Sharing life-altering information: development of pediatric hospital guidelines and team training. *J. Palliat. Med.* **17**, 1011–1018 (2014).

52. Okonofua, F., Lambo, E., Okeibunor, J. & Agholor, K. Advocacy for free maternal and child health care in Nigeria—Results and outcomes. *Health Policy* **99**, 131–138 (2011).

53. Carmona J.M.; Howe E.; Zapata R.; Stevens D.; Murphy J.K. The teen health improvement program: A system-wide initiative to improve the quality of care for adolescents served by the country's largest public hospital system. in (2015).

54. Alvarez, E. *et al.* Developing evidence briefs for policy: a qualitative case study comparing the process of using a guidance-contextualization workbook in Peru and Uganda. *Health Res. Policy Syst.* **17**, 89 (2019).

For peer review only

55. Barnard, J. G. *et al.* Facilitators and barriers to the use of standing orders for vaccination in obstetrics and gynecology settings. *Am. J. Obstet. Gynecol.* **216**, 69.e1-69.e7 (2017).

56. Becker-Haimes, E. M., Franklin, M., Bodie, J. & Beidas, R. S. Feasibility and Acceptability of a Toolkit to Facilitate Clinician Use of Exposure Therapy for Youth. *Evid.-Based Pract. Child Adolesc. Ment. Health* **2**, 165–178 (2017).

57. Boyko, J. A., Kothari, A. & Wathen, C. N. Moving knowledge about family violence into public health policy and practice: a mixed method study of a deliberative dialogue. *Health Res. Policy Syst.* **14**, 31 (2016).

58. Braddick, L. *et al.* A mixed-methods study of barriers and facilitators to the implementation of postpartum hemorrhage guidelines in Uganda. *Int. J. Gynaecol. Obstet. Off. Organ Int. Fed. Gynaecol. Obstet.* **132**, 89–93 (2016).

59. Burgoine, K. *et al.* Staged implementation of a two-tiered hospital-based neonatal care package in a resource-limited setting in Eastern Uganda. *BMJ Glob. Health* **3**, (2018).

60. Close, K., Karel, M. & White, M. A pilot program of knowledge translation and implementation for newborn resuscitation using US Peace Corps Volunteers in rural Madagascar. *Glob. Health* **12**, (2016).

61. Edwards, N. *et al.* The impact of leadership hubs on the uptake of evidence-informed nursing practices and workplace policies for HIV care: a quasi-experimental study in Jamaica, Kenya, Uganda and South Africa. *Implement. Sci. IS* **11**, 110 (2016).

62. Finch, M. *et al.* Challenges of Increasing Childcare Center Compliance With Nutrition Guidelines: A Randomized Controlled Trial of an Intervention Providing Training, Written Menu Feedback, and Printed Resources. *Am. J. Health Promot. AJHP* **33**, 399–411 (2019).

63. George E.; Maloney F.; Kara N.; Bukoye B.; Benotti E.; Delaney M.M.; Nejad S.B.; Kumar N.D.; Semrau K. FACTORS DETERMINING THE UTILIZATION OF

For peer review only

ANTENATAL CARE IN RURAL AND URBAN SETTINGS IN MOZAMBIQUE. *Int. J. Gynecol. Obstet.* **143**, 523 (2018).

64. Gera, R. *et al.* Implementation of "health systems approach" to improve vaccination at birth in institutional deliveries at public health facilities; experience from six states of India. *J. Fam. Med. Prim. Care* **8**, 1630–1636 (2019).

65. Gichane, M. W., Mutesa, M. & Chowa, G. Translating Evidence into Policy Change: Advocacy for Community-Based Distribution of Injectable Contraceptives in Zambia. *Glob. Soc. Welf.* **6**, 41–47 (2019).

66. Gilkey, M. B., Parks, M. J., Margolis, M. A., McRee, A.-L. & Terk, J. V. Implementing Evidence-Based Strategies to Improve HPV Vaccine Delivery. *Pediatrics* **144**, (2019).

67. Grady, A. *et al.* Effectiveness of a dissemination strategy on the uptake of an online menu planning program: A controlled trial. *Health Promot. J. Aust. Off. J. Aust. Assoc. Health Promot. Prof.* **30 Suppl 1**, 20–25 (2019).

68. Guillory, C. *et al.* Texas Pulse Oximetry Project: A Multicenter Educational and Quality Improvement Project for Implementation of Critical Congenital Heart Disease Screening Using Pulse Oximetry. *Am. J. Perinatol.* **34**, 856–860 (2017).

69. Hardee, K. *et al.* Improving Voluntary, Rights-Based Family Planning: Experience From Nigeria And Uganda. *Open Access J. Contracept.* **10**, 55–67 (2019).

70. Kraft, J. M. *et al.* Dissemination and use of WHO family planning guidance and tools: a qualitative assessment. *Health Res. Policy Syst.* **16**, 42 (2018).

71. Makkar, S. R., Howe, M., Williamson, A. & Gilham, F. Impact of tailored blogs and content on usage of Web CIPHER - an online platform to help policymakers better engage with evidence from research. *Health Res. Policy Syst.* **14**, 85 (2016).

72. Ongolo-Zogo, P., Lavis, J. N., Tomson, G. & Sewankambo, N. K. Assessing the influence of knowledge translation platforms on health system policy processes to

For peer review only

achieve the health millennium development goals in Cameroon and Uganda: a comparative case study. *Health Policy Plan.* **33**, 539–554 (2018).

73. Segre, L. S., Trusty, S., Gullickson, R., Chuffo Davila, R. & O'Hara, M. W. Brokering the Evidence-Practice Gap: A Strategy for Moving Evidence Into Clinical Practice. *Psychiatr. Serv. Wash. DC* **69**, 852–854 (2018).
74. Seward, K. *et al.* Improving the implementation of nutrition guidelines in childcare centres improves child dietary intake: findings of a randomised trial of an implementation intervention. *Public Health Nutr.* **21**, 607–617 (2018).
75. Uneke, C. J., Sombie, I., Uro-Chukwu, H. C. & Johnson, E. Using equitable impact sensitive tool (EQUIST) to promote implementation of evidence informed policymaking to improve maternal and child health outcomes: a focus on six West African Countries. *Glob. Health* **14**, 104 (2018).
76. Uneke, C. J., Sombie, I., Uro-Chukwu, H. C., Johnson, E. & Okonofua, F. Using equitable impact sensitive tool (EQUIST) and knowledge translation to promote evidence to policy link in maternal and child health: report of first EQUIST training workshop in Nigeria. *Pan Afr. Med. J.* **28**, 37 (2017).
77. Vlad, I. *et al.* Improving quality for maternal care - a case study from Kerala, India. *F1000Research* **5**, (2016).
78. Wolfenden, L. *et al.* Multi-strategic intervention to enhance implementation of healthy canteen policy: a randomised controlled trial. *Implement. Sci. IS* **12**, (2017).
79. Yoong, S. L. *et al.* A theory-based evaluation of a dissemination intervention to improve childcare cooks' intentions to implement nutritional guidelines on their menus. *Implement. Sci.* **11**, 105 (2016).

For peer review only

For peer review only - http://bmjopen.bmj.com/site/about/guidelines.xhtml

80. Kingsnorth, S., Orava, T., Parker, K. & Milo-Manson, G. From knowledge translation theory to practice: developing an evidence to care hub in a pediatric rehabilitation setting. *Disabil. Rehabil.* **42**, 869–879 (2020).

81. Eriksson, L., Bergström, A., Hoa, D. T. P., Nga, N. T. & Eldh, A. C. Sustainability of knowledge implementation in a low- and middle- income context: Experiences from a facilitation project in Vietnam targeting maternal and neonatal health. *PLOS ONE* **12**, e0182626 (2017).

82. Simioni, A. T. *et al.* [Regionalization of perinatal health care in the province of Santa Fe, Argentina]. *Rev. Panam. Salud Publica Pan Am. J. Public Health* **41**, e38 (2017).

83. Cunningham, B. J. & Cardy, J. O. Using implementation science to engage stakeholders and improve outcome measurement in a preschool speech-language service system. *Speech Lang. Hear.* **23**, 17–24 (2020).

84. Agency for Healthcare Research and Quality. *AHRQ Safety Program for Perinatal Care: Experiences From the Frontline. Prepared under contract no. 2902010000241 (RTI International). AHRQ Publication No. 17-0003-23-EF. Rockville, MD: Agency for Healthcare Research and Quality. October 30, 2016. www.ahrq.gov/perinatalsafety.* (2016).

85. Akter, S. F. U., Heller, R. D., Smith, A. J. & Milly, A. F. Impact of a training intervention on use of antimicrobials in teaching hospitals. *J. Infect. Dev. Ctries.* **3**, 447–451 (2009).

86. Allen, C. W. & Jeffery, H. Implementation and evaluation of a neonatal educational program in rural Nepal. *J. Trop. Pediatr.* **52**, 218–222 (2006).

87. Berglund, A., Lefevre-Cholay, H., Bacci, A., Blyumina, A. & Lindmark, G. Successful implementation of evidence-based routines in Ukrainian maternities. *Acta Obstet. Gynecol. Scand.* **89**, 230–237 (2010).

For peer review only

88. Deorari, A. K., Paul, V. K., Singh, M., Vidyasagar, D., & Medical Colleges Network. Impact of education and training on neonatal resuscitation practices in 14 teaching hospitals in India. *Ann. Trop. Paediatr.* **21**, 29–33 (2001).

89. Ding, H. *et al.* Influencing the use of antibiotics in a Chinese pediatric intensive care unit. *Pharm. World Sci. PWS* **30**, 787–793 (2008).

90. Jeffery, H. E. *et al.* The impact of evidence-based education on a perinatal capacity-building initiative in Macedonia. *Med. Educ.* **38**, 435–447 (2004).

91. Uskun, E., Uskun, S. B., Uysalgenc, M. & Yagiz, M. Effectiveness of a training intervention on immunization to increase knowledge of primary healthcare workers and vaccination coverage rates. *Public Health* **122**, 949–958 (2008).

92. Warren, C., Mwangi, A., Oweya, E., Kamunya, R. & Koskei, N. Safeguarding maternal and newborn health: improving the quality of postnatal care in Kenya. *Int. J. Qual. Health Care* **22**, 24–30 (2010).

93. Agency for Healthcare Research and Quality. *Care Transitions from Hospital to Home: IDEAL Discharge Planning Implementation Handbook. Guide to Patient and Family Engagement*.

94. Alton, M., Frush, K., Brandon, D. & Mericle, J. Development and implementation of a pediatric patient safety program. *Adv. Neonatal Care Off. J. Natl. Assoc. Neonatal Nurses* **6**, 104–111 (2006).

95. Brown, J. J., Wacogne, I., Fleckney, S., Jones, L. & Bhrolchain, C. N. Achieving early surgery for undescended testes: quality improvement through a multifaceted approach to guideline implementation. *Child Care Health Dev.* **31**, 119–119 (2005).

96. Carlo, W. A. *et al.* Educational impact of the neonatal resuscitation program in low-risk delivery centers in a developing country. *J. Pediatr.* **154**, 504-508.e5 (2009).

For peer review only

97. Crone, M. R. *et al.* Sustainability of the Prevention of Passive Infant Smoking Within Well-Baby Clinics. *Health Educ. Behav.* **33**, 178–196 (2006).

98. Margolis, P. A. *et al.* From concept to application: the impact of a community-wide intervention to improve the delivery of preventive services to children. *Pediatrics* **108**, E42 (2001).

99. Pariyo, G. W., Gouws, E., Bryce, J., Burnham, G., & Uganda IMCI Impact Study Team. Improving facility-based care for sick children in Uganda: training is not enough. *Health Policy Plan.* **20 Suppl 1**, i58–i68 (2005).

100. Schreiber, J., Stern, P., Marchetti, G. & Provident, I. Strategies to promote evidence-based practice in pediatric physical therapy: a formative evaluation pilot project. *Phys. Ther.* **89**, 918–933 (2009).

101. R Simmons, Fajans, P. & Ghiron, L. Chapter 2: Strategic choices in scaling up: introducing injectable contraception and improving quality of care in Viet Nam. in *Scaling up health service delivery: from pilot innovations to policies and programmes* (World health Organization, 2007).

102. Goering, M. & Wilson, W. Implementing preterm labor guidelines: a collaborative care improvement process. *J. Perinat. Neonatal Nurs.* **16**, 47–57 (2002).

103. Davies, B. *et al.* Fetal health surveillance: a community-wide approach versus a tailored intervention for the implementation of clinical practice guidelines. *CMAJ Can. Med. Assoc. J. J. Assoc. Medicale Can.* **167**, 469–474 (2002).

104. English, K. C., Merzel, C. & Moon-Howard, J. Translating public health knowledge into practice: development of a lay health advisor perinatal tobacco cessation program. *J. Public Health Manag. Pract. JPHMP* **16**, E9–E19 (2010).

For peer review only

105. Russell, D. J. *et al.* Using knowledge brokers to facilitate the uptake of pediatric measurement tools into clinical practice: a before-after intervention study. *Implement. Sci. IS* **5**, 92 (2010).

106. R Simmons, Fajans, P. & Ghiron, L. Chapter 7 Scaling up family planning service innovations in Brazil: the influence of politics and decentralization. in *Scaling up health service delivery: from pilot innovations to policies and programmes* (World health Organization, 2007).

107. Di Noia, J., Schwinn, T. M., Dastur, Z. A. & Schinke, S. P. The relative efficacy of pamphlets, CD-ROM, and the Internet for disseminating adolescent drug abuse prevention programs: an exploratory study. *Prev. Med.* **37**, 646–653 (2003).

108. Dobbins, M. *et al.* A randomized controlled trial evaluating the impact of knowledge translation and exchange strategies. *Implement. Sci.* **4**, 61 (2009).

109. Melnyk, B. M. *et al.* Translating the evidence-based NICU COPE program for parents of premature infants into clinical practice: impact on nurses' evidence-based practice and lessons learned. *J. Perinat. Neonatal Nurs.* **24**, 74–80 (2010).

110. Rowe, A. K. *et al.* A multifaceted intervention to improve health worker adherence to integrated management of childhood illness guidelines in Benin. *Am. J. Public Health* **99**, 837–846 (2009).

111. Senarath, U., Fernando, D. N. & Rodrigo, I. Effect of training for care providers on practice of essential newborn care in hospitals in Sri Lanka. *J. Obstet. Gynecol. Neonatal Nurs. JOGNN* **36**, 531–541 (2007).

112. Snelgrove-Clarke, E. E. The effects of action learning on nurses' use of a fetal health surveillance guideline with low-risk labouring women. https://escholarship.mcgill.ca/concern/theses/dz010q44w.

For peer review only

113. Daniels, K., Lewin, S., & Practice Policy Group. Translating research into maternal health care policy: a qualitative case study of the use of evidence in policies for the treatment of eclampsia and pre-eclampsia in South Africa. *Health Res. Policy Syst.* **6**, 12 (2008).

114. Organisation for Economic Co-operation and Development. States of Fragility 2020. https://www.oecd-ilibrary.org/development/states-of-fragility-2020_ba7c22e7-en (2020).

115. Santesso, N. & Tugwell, P. Knowledge translation in developing countries. *J. Contin. Educ. Health Prof.* **26**, 87–96 (2006).

116. Nabyonga Orem, J. *et al.* Perspectives on the role of stakeholders in knowledge translation in health policy development in Uganda. *BMC Health Serv. Res.* **13**, 324 (2013).

117. World Health Organization. Millennium Development Goals (MDGs). https://www.who.int/news-room/fact-sheets/detail/millennium-development-goals-(mdgs) (2018).

118. Fenny, A. P., Crentsil, A. O. & Ackah, C. The health MDGs in Ghana: lessons and implications for the implementation of the sustainable development goals. *J. Public Health* **26**, 225–234 (2018).

119. Bendavid, E. *et al.* The effects of armed conflict on the health of women and children. *The Lancet* **397**, 522–532 (2021).

120. Stark, L., Seff, I. & Reis, C. Gender-based violence against adolescent girls in humanitarian settings: a review of the evidence. *Lancet Child Adolesc. Health* **5**, 210–222 (2021).

For peer review only

121. Oduro-Mensah, E. *et al.* Care Decision Making of Frontline Providers of Maternal and Newborn Health Services in the Greater Accra Region of Ghana. *PLOS ONE* **8**, e55610 (2013).

122. UNICEF. Adolescent health and well-being. https://www.unicef.org/health/adolescent-health-and-well-being (2021).

123. Partnership for MAternal, Newborn and Child health. Adolescent health and well-being. *WHO* http://www.who.int/pmnch/media/news/2019/Adolescent_health_and_well-being_webinars/en/ (2020).

124. World Health Organization. WHO | Aligning public financial management and health financing. *WHO* http://www.who.int/health_financing/documents/aligning-pfm-health-financing/en/ (2017).

125. World Health Organization Regional Office for South-East Asia. Continuing essential Sexual Reproductive, Maternal, Neonatal, Child and Adolescent Health services during COVID-19 pandemic. (2020).

126. Dal Mas, F., Biancuzzi, H., Massaro, M. & Miceli, L. Adopting a knowledge translation approach in healthcare co-production. A case study. *Manag. Decis.* **58**, 1841–1862 (2020).

127. Graham, I. D. & Logan, J. Innovations in knowledge transfer and continuity of care. *Can. J. Nurs. Res. Rev. Can. Rech. En Sci. Infirm.* **36**, 89–103 (2004).

128. Graham, I. D. *et al.* Lost in knowledge translation: Time for a map? *J. Contin. Educ. Health Prof.* **26**, 13–24 (2006).

129. Rosenthal, R. The file drawer problem and tolerance for null results. *Psychol. Bull.* **86**, 638–641 (1979).

For peer review only

For peer review only

Figure 1. PRISMA-ScR flow diagram of literature search

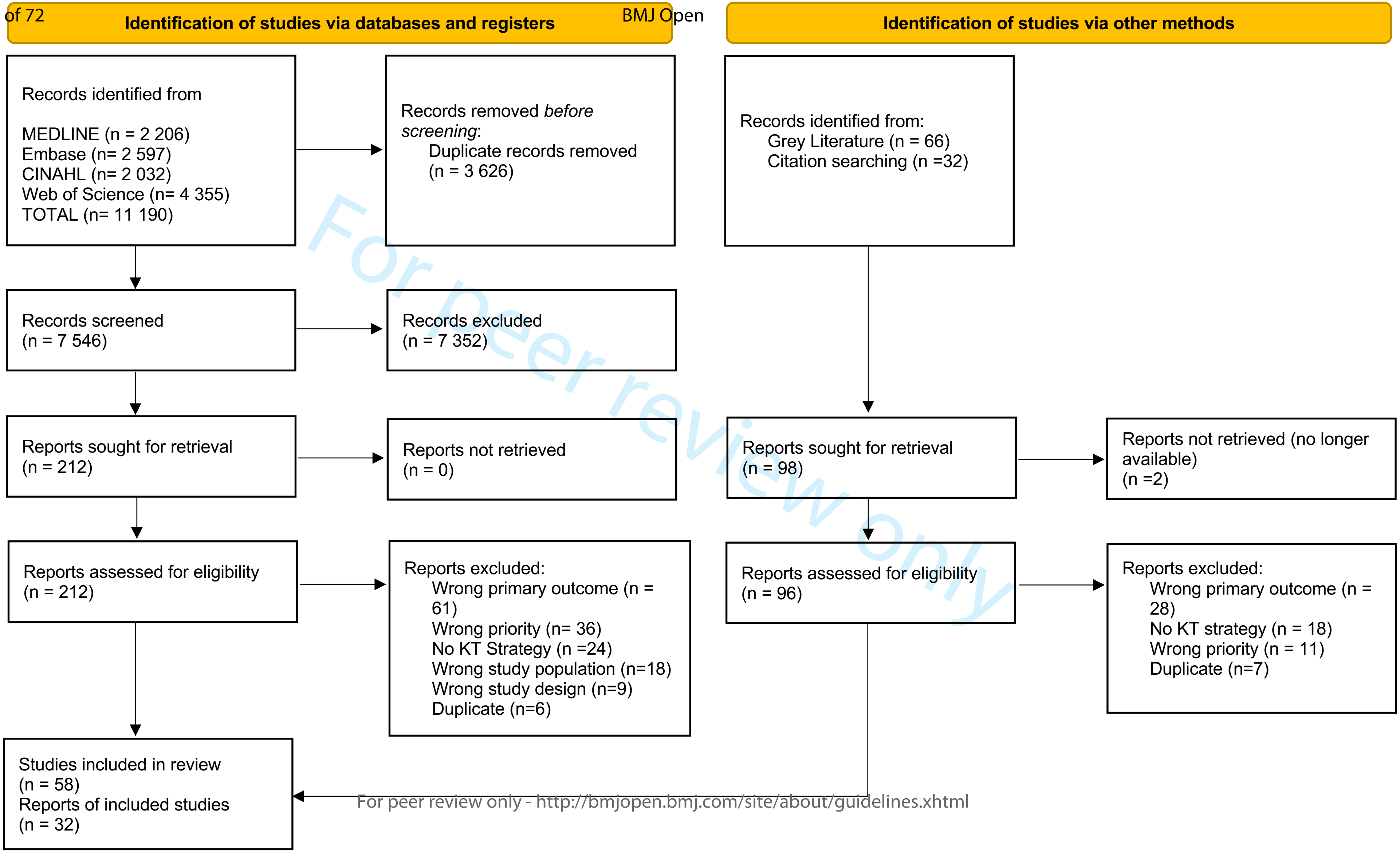

For peer review only

Supplementary File 1- Search Strategy

1 exp Diffusion of Innovation/
2 Information Dissemination/
3 Translational Medical Research/
4 ((disseminat* or implement* or share? or sharing or translat*) adj2 (activities or activity or approach? or model? or plan? or planning or program* or strategies or strategy or advanc* or better* or boost* or enhanc* or forward* or further* or improv* or increas* or bolster* or sustain* or uphold*)).ti,ab,kw,kf.
5 (evidence* adj2 (adapt* or adopt* or diffus* or disseminat* or exchang* or implement* or mobili* or share? or sharing or transfer* or translat* or utilis* or utiliz* or uptake)).ti,ab,kw,kf.
6 (information adj2 (adapt* or adopt* or diffus* or disseminat* or exchang* or mobili* or propagat* or share? or sharing or spread* or transfer* or translat*)).ti,ab,kw,kf.
7 (innovation? adj2 (adapt* or adopt* or diffus* or disseminat* or exchang* or implement* or mobili* or propagat* or share? or sharing or spread* or transfer* or translat* or transmi* or uptake)).ti,ab,kw,kf.
8 (knowledge adj2 (adapt* or adopt* or diffus* or disseminat* or exchang* or implement* or mobili* or propagat* or share? or sharing or spread* or transfer* or translat* or transmi* or uptake)).ti,ab,kw,kf.
9 (research adj2 (adapt* or adopt* or diffus* or disseminat* or exchang* or implement* or mobili* or propagat* or share? or sharing or spread* or transfer* or translat* or transmi* or utilis* or utiliz* or uptake)).ti,ab,kw,kf.
10 or/1-9 [KT]
11 Cartoons as Topic/
12 exp Mass Media/
13 Pamphlets/
14 Newspapers as Topic/
15 Webcasts as Topic/
16 Guidelines as Topic/
17 ((knowledge or evidence) adj (product? or summary or summaries or brief* or report? or portal? or repositor* or database? or center? or centre?)).ti,ab,kw,kf.
18 (advocacy brief* or strategy brief*).ti,ab,kw,kf.
19 (position statement? or consensus statement? or policy statement?).ti,ab,kw,kf.
20 (booklet? or brochure? or cartoon* or comic? or flyer? or infograph* or pamphlet? or poster?).ti,ab,kw,kf.
21 (media adj2 (communicat* or diffus* or disseminat* or exchang* or mobili* or populari* or propagat* or publici* or share? or sharing or spread*)).ti,ab,kw,kf.
22 (briefing? or news or newspaper? or press release?).ti,ab,kw,kf.
23 (thematic report? or accountability report? or evaluation report?).ti,ab,kw,kf.
24 (strategic plan? or annual report? or progress report?).ti,ab,kw,kf.
25 (online communication? or web communication? or video? or webcast* or podcast*).ti,ab,kw,kf.
26 (toolkit? or tool or tools or roadmap? or road map?).ti,ab,kw,kf.
27 (guideline? or standards).ti,ab,kw,kf.

For peer review only

28 or/11-27 [products]
29 10 and 28 [KT products]
30 ((evidence or information or innovation? or knowledge or research) adj2 (adapt* or adopt* or diffus* or disseminat* or exchang* or implement* or mobili* or propagat* or share? or sharing or spread* or transfer* or translat* or transmi* or uptake) adj2 (activities or activity or approach? or initiative? or plan? or policies or policy or procedure? or program* or scheme? or strategies or strategy)).ti,ab,kw,kf.
31 or/1-3
32 (activities or activity or approach? or initiative? or plan? or policies or policy or procedure? or program* or scheme? or strategies or strategy).ti.
33 31 and 32 [KT activities]
34 30 or 33 [KT activities or strategies]
35 29 or 34 [KT products, activities, or strategies (INTERVENTION)]
36 exp Administrative Personnel/
37 exp Policy Making/
38 (administrator? or decision maker? or policy maker? or policymaker?).ti,ab.
39 (knowledge user? or stakeholder?).ti,ab.
40 (advisory or board or committee? or task force?).ti,ab.
41 (((policy or policies) adj making) or policymaking or ((policy or policies) adj2 develop*) or ((policy or policies) adj2 analys?s) or (health adj (policy or policies))).ti,ab.
42 (legislator? or legislation?).ti,ab.
43 (health system? or health care system? or healthcare system? or system? level or system? manage*).ti,ab.
44 (civil societ* or third sector or non government* organi?ation? or nongovernment* organi?ation? or ngo or ngos).ti,ab.
45 (donor? or funder? or funding agen* or grant* agen*).ti,ab.
46 or/36-45 [POPULATION]
47 Reproductive Health/ or Reproductive Health Services/ or Family Planning Services/ or Sexual Health/ or Circumcision, Female/ or Menstruation/ or exp Puberty/ or exp Contraception/ or exp Sexually Transmitted Diseases/ or exp Sex Offenses/ or Intimate Partner Violence/ or Gender-Based Violence/ or Sex Work/ or exp Human papillomavirus 16/ or Human papillomavirus 18/ or Uterine Cervical Neoplasms/ or (reproductive or reproduction or family planning or sexual* or early marriage or child marriage or child bride? or female genital mutilation or female circumcision or menstruation or menstrual or puberty or contraception or contraceptive* or condom? or sexually transmitted infection* or sexually transmitted disease* or hiv or aids or coerc* or rape or forced sex or abortion? or intimate partner violence or gender based violence or sex work* or transactional sex or hpv or cervical cancer?).ti,ab,kw,kf.
48 exp Women's Health/ or exp Women's Health Services/ or Women/ or (wom#n? or female? or mother? or maternal or widow?).ti,ab,kw,kf.
49 exp Maternal Health Services/ or Pregnant Women/ or exp Pregnancy/ or (birth or childbirth or mother* or maternal* or maternity or preconception or prenatal* or perinatal* or antepartum or ante-partum or intrapartum or intra-partum or neonatal* or neo-natal* or postnatal* or post-natal* or postpartum or post-partum or pregnan*).ti,ab,kw,kf.

For peer review only

50 exp Infant/ or (fetus* or foetus* or fetal* or foetal* or baby or babies or neonate* or neo-nate* or newborn* or new-born* or infant*).ti,ab,kw,kf.

51 exp Child/ or exp Pediatrics/ or (child* or kid or kids or girl or girls or boy or boys or preschool* or pre-school* or kindergarten* or school age* or elementary school* or juvenile* or minors or p?ediatric? or first-grader* or second-grader* or third-grader* or fourth-grader* or fifth-grader* or sixth-grader* or seventh-grader* or eighth-grader* or middle school* or junior high*).ti,ab,kw,kf.

52 Adolescent/ or Young Adult/ or (teen* or youth* or adolescen* or juvenile* or (young adj2 (adult* or person* or individual* or people* or population*)) or youngster* or highschool* or ((secondary or high*) adj2 (school* or education)) or pubescen*).ti,ab,kw,kf.

53 or/47-52 [RMNCAH filter]

54 35 and 46 and 53

For peer review only

Supplementary File 2- Description of Included Studies

Author & Year	Country	Country Income Level	Primary Objective	Health Topic	SRMNCAH Priority	Study Design	Sample Characteristics	BCW Intervention Functions Summary	BCW Policy Categories Summary	Direction of Effect
Abney-Roberts et al., 2015[1]	USA	High	To prevent sleep-related infant deaths by applying recommendations from the American Academy of Pediatrics	Newborn sleep	Newborn health/wellbeing or stillbirths	Quasi-experimental	150 healthcare providers (e.g., nurses and physicians from obstetrics, pediatrics and family medicine)	Education		Positive
Agapidaki et al., 2013[2]	Greece	High	To improve pediatricians' ability to identify and manage maternal depression by implementing a targeted education program	Maternal depression	Maternal health/wellbeing	Experimental	43 pediatricians	Education<br><br>Environmental Restructuring<br><br>Persuasion	Guidelines	Positive
Agency for Healthcare Research and Quality, 2016[3]	USA	High	To decrease maternal and neonatal adverse events, and to improve patient safety, team communication, and quality of care	Perinatal care	Maternal health/wellbeing<br><br>Newborn health/wellbeing or stillbirths	Observational	Labour & delivery units	Education<br><br>Environmental Restructuring<br><br>Training	Service Provision	Positive
Ainsworth et al., 2014[4]	USA	High	To reduce and prevent newborn falls	Newborn fall prevention	Newborn health/wellbeing or stillbirths	Quasi-experimental	Healthcare providers, mothers and their infants	Education<br><br>Environmental Restructuring	Regulation	Positive
Akter et al., 2009[5]	Bangladesh	Middle	To identify changes in antimicrobial use following the implementation of an educational intervention targeting physicians	Antimicrobial use	Child health/wellbeing	Quasi-experimental	All physicians working in pediatric wards at the participating hospital	Education		Positive
Al-Rafay & Al-Sharkawy, 2012[6]	Egypt	Middle	To evaluate changes in nurses' knowledge following an education program to address total parenteral nutrition guidelines	Newborn nutrition	Newborn health/wellbeing or stillbirths	Quasi-experimental	40 nurses	Education<br><br>Training	Guidelines	Positive
Alkon et al., 2014[7]	USA	High	To evaluate a nutrition and physical activity program implemented at childcare centres	Child nutrition and physical activity	Child health/wellbeing	Experimental	137 childcare providers from 17 childcare centres. Centres included a total of 552 children who were in the program (aged 3-5 years)	Education<br><br>Environmental Restructuring	Guidelines	Positive

Allen & Jeffery, 2006[8]	Nepal	Middle	To design and implement an educational newborn care program targeting healthcare providers to decrease infant morbidity and mortality	Newborn care	Newborn health/wellbeing or stillbirths	Quasi-experimental	30 healthcare providers (e.g., junior doctors, nurses, community health workers)	Education		Positive
Allen & Schafer, 2015[9]	USA	High	To implement practices which promote optimal infant feeding	Infant nutrition	Newborn health/wellbeing or stillbirths	Quasi-experimental	Healthcare providers, pregnant women, and new mothers and their infants	Education<br><br>Environmental Restructuring<br><br>Enablement	Service provision<br><br>Communication/ Marketing<br><br>Environmental/ Social Planning	Positive
Alton et al. 2006[10]	USA	High	To describe a pediatric patient safety program	Pediatric patient safety	Child health/wellbeing	Observational	Healthcare providers, health systems managers	Education<br><br>Environmental Restructuring	Service provision<br><br>Communication/ Marketing<br><br>Environmental/ Social Planning<br><br>Regulation	Positive
Alvarez et al., 2019[11]	Peru and Uganda	Low and middle	To determine the useability and usefulness of a workbook to provide support and guidance in global health systems	Maternal and newborn health	Maternal Health/Wellbeing<br><br>Newborn health/wellbeing or stillbirths	Qualitative	8 participants (e.g., researchers, policymakers or other stakeholders)	Education	Guidelines	Positive
Anaby et al., 2015[12]	Canada	High	To improve healthcare providers' knowledge and to change practice to support children with disabilities	Activities with children and youth with physical disabilities	Child health/wellbeing	Qualitative	14 healthcare providers (e.g., occupational therapists, physiotherapists, speech language pathologist)	Education	Service Provision	Positive
Ansbro et al., 2015[13]	Zambia	Middle	To explore healthcare providers' experience of using rapid syphilis testing	Syphilis	Sexual or reproductive health/rights	Qualitative	40 healthcare providers		Guidelines	Positive
Baer et al., 2011[14]	USA	High	To determine the effectiveness of a NICU blood transfusion and monitoring system 12 months following implementation	Neonatal blood transfusions	Newborn health/wellbeing or stillbirths	Quasi-experimental	Four NICUs	Environmental Restructuring<br><br>Enablement<br><br>Coercion<br><br>Restriction	Service Provision	Positive
Barnard et al., 2017[15]	USA	High	To identify barriers and enablers to establishing standing orders to vaccinate during pregnancy	Vaccination during pregnancy	Maternal health/wellbeing	Qualitative	38 healthcare providers and staff members (e.g., medical directors,	Education	Service Provision	Positive

For peer review only

							practice administrators, nurses, medical assistants)			
Becker-Haimes et al., 2017[16]	USA	High	To explore the acceptability and feasibility of a toolkit designed for healthcare providers working across mental health settings.	Youth mental health	Adolescent health/wellbeing	Mixed methods	Quantitative: 70 clinicians<br><br>Qualitative: Six randomly selected clinicians from the quantitative study	Education		Positive
Berglund et al., 2010[17]	Ukraine	Middle	To determine the effectiveness of the W.H.O's Effective Perinatal Care package implemented at maternity hospitals	Perinatal care	Maternal Health/Wellbeing	Quasi-experimental	Healthcare providers at three maternity hospitals (e.g., obstetricians, neonatologists, pediatricians, anesthesiologists, midwives, pediatric nurses)	Environmental Restructuring<br><br>Training<br><br>Enablement		Positive
Boyko et al., 2016[18]	Canada	High	To explore the usefulness of deliberative dialogue utilized by the Preventing Violence Across the Lifespan (PreVAiL) Research Network during a biennial team meeting	Child maltreatment	Child Health/Wellbeing	Mixed methods	44 participants in the deliberative dialogue (e.g., researchers, knowledge users, policymakers)	Education		Positive
Braddick et al., 2016[19]	Uganda	Low	To assess compliance with postpartum hemorrhage clinical guideline recommendations, and identify barriers and enablers to using the recommendations	Postpartum hemorrhage	Maternal Health/Wellbeing	Mixed methods	Quantitative: 154 births<br>Qualitative: 18 healthcare provider interviews (e.g., physicians, midwives)	Education	Guidelines	Neutral
Brennan et al., 2013[20]	Ghana	Middle	To increase nurses' knowledge and skills in pediatric resuscitation through the WHO Emergency Triage and Assessment Treatment program	Pediatric resuscitation	Child Health/Wellbeing	Quasi-experimental	41 nurses	Education<br><br>Training<br><br>Modelling		Positive
Brown et al., 2005[21]	UK	High	To identify the barriers creating delays in surgery and develop guidelines for management of undescended testes	Undescended testes	Newborn Health/Wellbeing or Stillbirths	Observational	Healthcare providers and hospital staff	Education<br><br>Environmental Restructuring	Service Provision<br><br>Guidelines	Positive

Burgoine et al., 2018[22]	Uganda	Low	To reduce neonatal mortality through the implementation of two neonatal health interventions	Neonatal mortality	Newborn Health/Wellbeing or Stillbirths	Quasi-experimental	57 healthcare providers (e.g., midwives, nurses, interns)	Education<br><br>Environmental Restructuring<br><br>Training<br><br>Enablement	Guidelines<br><br>Environmental/ Social Planning	Positive
Cameron et al., 2011[23]	Canada	High	To determine healthcare administrators' perceptions of the barriers and enablers in using a knowledge broker to support implementing measures aimed at children with cerebral palsy needs	Cerebral palsy	Child Health/Wellbeing	Qualitative	Healthcare administrators from 27 pediatric healthcare centres	Education<br><br>Environmental Restructuring	Service Provision	Positive
Carlo et al., 2009[24]	Zambia	Middle	To improve nurse midwives' knowledge and skills following completion of an American Academy of Pediatrics Neonatal Resuscitation Program	Neonatal resuscitation	Newborn Health/Wellbeing or Stillbirths	Observational	127 nurse midwives	Education<br><br>Training		Positive
Carmona et al., 2015[25]	USA	High	To improve healthcare providers' ability to provide high quality adolescent healthcare	Teen health	Adolescent Health/Wellbeing	Mixed methods	109 healthcare providers from 15 sites	Training	Guidelines	Positive
Close et al., 2016[26]	Madagascar	Low	To determine the effectiveness of a knowledge translation and implementation model aimed at training Peace Corps Volunteers to then train healthcare staff in rural communities	Neonatal resuscitation	Newborn Health/Wellbeing or Stillbirths	Quasi-experimental	10 Peace Corps Volunteers trained 42 healthcare providers in 10 communities	Training<br><br>Enablement		Positive
Crone et al., 2006[27]	Netherland s	High	To determine long-term use of a smoking cessation program to prevent passive smoking around children	Baby exposure to smoke in the household	Newborn Health/Wellbeing or Stillbirths	Observational	39 baby health clinic managers, 255 nurses and 68 physicians	Education<br><br>Training	Communication/ Marketing	Neutral
Cunningham & Cardy, 2020[28]	Canada	High	To improve speech language pathologists' knowledge and intention to utilize a tool designed to support participation-focused outcome measurements	Child speech	Child Health/Wellbeing	Quasi-experimental	46 speech language pathologists	Education<br><br>Persuasion		Positive
Daniels et al., 2008[29]	South Africa	Middle	To determine perceived and actual utilization of research in policy and legislation development	Eclampsia and pre-eclampsia	Maternal Health/Wellbeing	Qualitative	15 interviews with local researchers and policy makers	Education	Legislation	Positive

For peer review only

Davies et al., 2002[30]	Canada	High	To determine the effectiveness of an interactive, educational workshop to support nurses to implement fetal health surveillance guidelines	Labour and delivery	Maternal Health/Wellbeing	Mixed methods	Four hospitals sites (2 intervention, 2 control). Between 14-35 nurses from the intervention hospitals participated in each of the four education modules	Education<br><br>Environmental Restructuring<br><br>Training		Positive
Deorari et al., 2001[31]	India	Middle	To improve newborn health outcome and reduce incidence of newborn resuscitation by providing healthcare providers with neonatal resuscitation training	Neonatal resuscitation	Newborn Health/Wellbeing or Stillbirths	Quasi-experimental	Physicians and nurses at 14 hospitals	Environmental Restructuring<br><br>Training	Regulation	Positive
Di Noia et al., 2003[32]	USA	High	To promote a youth substance abuse prevention program through pamphlets, CD-ROMs, and the Internet to encourage use and recommendations for the program	Adolescent substance abuse prevention	Adolescent Health/Wellbeing	Experimental	188 staff members employed at junior high schools, youth-focused non-profit organizations, and government bodies aimed at preventing youth substance abuse	Education<br><br>Environmental Restructuring		Positive
Ding et al., 2008[33]	China	Middle	To reduce inappropriate antibiotic prescribing in the pediatric intensive care unit (PICU)	Antibiotic prescribing	Child Health/Wellbeing	Quasi-experimental	Pediatricians	Education<br><br>Environmental Restructuring	Guidelines<br><br>Environmental/ Social Planning	Positive
Dobbins et al., 2009[34]	Canada	High	To evaluate three KT strategies aimed at supporting healthy childhood bodyweight	Childhood obesity	Child Health/Wellbeing	Experimental	108 public health departments	Education<br><br>Environmental Restructuring<br><br>Training		Neutral
Edwards et al., 2016[35]	Jamaica, Kenya, Uganda and South Africa	Low and Middle	To improve HIV care by implementing nurse leadership hubs	HIV	Sexual or reproductive health/rights	Quasi-experimental	167 participants (e.g., nurses, staff nurses, managers, researchers, decision makers, community representatives)	Education<br><br>Environmental Restructuring<br><br>Training<br><br>Enablement	Communication/ Marketing	Neutral
English et al., 2010[36]	USA	High	To outline the design and implementation of a perinatal tobacco cessation program	Perinatal tobacco cessation	Maternal Health/Wellbeing	Mixed methods	11 lay case managers attended the first training session, 8 participated in the second session	Education<br><br>Training<br><br>Modelling		Neutral

For peer review only

Eriksson et al., 2017[37]	Vietnam	Middle	To identify barriers and enablers to implementation and sustainability of a knowledge translation strategy, the Neonatal Knowledge Into Practice (NeoKIP) trial	Neonatal mortality	Maternal Health/Wellbeing<br><br>Newborn Health/Wellbeing or Stillbirths	Qualitative	Interviews: 6 interviews with healthcare directors or representatives from healthcare facilities<br><br>Focus groups: 6 focus groups with previous stakeholders in the NeoKIP trial	Environmental Restructuring<br><br>Training		Positive
Farner et al., 2014[38]	USA	High	To improve healthcare providers' screening for critical congenital heart disease through the design, implement and evaluate an educational program	Newborn screening for critical congenital heart disease	Newborn Health/Wellbeing or Stillbirths	Quasi-experimental	215 nurses participated across 13 hospitals	Training<br><br>Modelling<br><br>Environmental restructuring<br><br>Education	Regulation	Positive
Finch et al., 2012[39]	Australia	High	To increase use of an intervention to encourage physical activity for children at childcare centres, and to improve childcare managers' knowledge of physical activity recommendations for children and perceived acceptability of the intervention	Childhood physical activity	Child Health/Wellbeing	Quasi-experimental	228 childcare centres participated in the intervention, 164 childcare centres were the control	Training<br><br>Enablement<br><br>Incentivisation		Positive
Finch et al., 2019[40]	Australia	High	To determine effectiveness of an intervention aimed at improving compliance with childcare nutrition guidelines in childcare centres	Childhood nutrition	Child Health/Wellbeing	Experimental	24 childcare centres participated in the intervention, 20 participated in the control from baseline data collection	Education<br><br>Training<br><br>Environmental Restructuring	Regulation	Neutral
George et al., 2018[41]	Sri Lanka	Middle	To hold a consultation with regional users of the WHO safe childbirth checklist to promote knowledge exchange, support improve maternal health outcomes, and offer high quality care during childbirth	Labour and delivery	Maternal Health/Wellbeing	Qualitative	60 participants (e.g., obstetricians, gynecologists, nurses, researchers, implementers, government officials, and 12 funders from low, middle, and high-income countries)	Education		Positive
Gera et al., 2019[42]	India	Middle	To evaluate an intervention aimed at improving newborn vaccination uptake in health facilities	Newborn vaccination	Newborn Health/Wellbeing or Stillbirths	Mixed Methods	141 public health facilities participated in the intervention<br><br>Qualitative: interviews with health managers	Training		Positive

For peer review only - http://bmjopen.bmj.com/site/about/guidelines.xhtml

Gichane et al., 2019[43]	Zambia	Middle	To outline the process of advocating for policy changes to allow for non-clinical healthcare workers to provide injectable contraception	Contraception/ Family planning	Sexual or reproductive health/rights	Editorial	Not reported	Education<br><br>Environmental Restructuring<br><br>Modelling	Legislation	Positive
Gilkey et al., 2019[44]	USA	High	To improve uptake of the HPV vaccine through use of a quality improvement program	HPV vaccination	Sexual or reproductive health/rights	Experimental	13 clinics received the intervention, while 12 were placed in the wait-list control group. 43 pediatric physicians participated across the intervention clinics, while 35 participated across the control clinics	Training<br><br>Incentivisation		Neutral
Goering & Wilson, 2002[45]	USA	High	To decrease variance and improve management of pre-term labour through the guideline implementation	Preterm labour	Maternal Health/Wellbeing	Observational	Nursing staff	Education<br><br>Enablement	Guidelines<br><br>Environmental/ Social planning	Positive
Goyet et al., 2014[46]	Cambodia	Middle	To identify barriers to KT strategies aimed at pneumonia	Pneumonia	Child Health/Wellbeing	Observational	Interventions targeted clinicians, health managers, policymakers	Education<br><br>Environmental Restructuring<br><br>Enablement		Neutral
Grady et al., 2019[47]	Australia	High	To determine effectiveness of a strategy aimed at improving compliance with dietary guidelines and daycare centres	Childhood nutrition	Child Health/Wellbeing	Quasi-experimental	27 daycare centres participated in the intervention services, 19 centres participated in the control services	Environmental Restructuring<br><br>Training<br><br>Enablement<br><br>Persuasion	Service Provision	Positive
Guillory, et al., 2017[48]	USA	High	To identify an appropriate implementation strategy for newborn screening for critical congenital heart disease across multiple hospitals	Newborn screening for critical congenital heart disease	Newborn Health/Wellbeing or Stillbirths	Quasi-experimental	117 physicians, hospital administrators and other healthcare professionals	Education<br><br>Environmental Restructuring<br><br>Training<br><br>Modelling		Positive

Hardee et al., 2019[49]	Nigeria and Uganda	Low and Middle	To determine effectiveness of two interventions which utilize a voluntary, rights-based family planning (VRBFP) approach to measure health and rights outcomes	Family planning	Sexual or reproductive health/rights	Mixed Methods	15 intervention healthcare facilities<br><br>Qualitative: 16 interviews with healthcare providers, health facility managers, supervisors, and representatives from collaborating human rights organizations<br><br>Focus Groups: male champions for the project, and facility and community healthcare workers	Education<br><br>Environmental Restructuring<br><br>Training<br><br>Enablement		Positive
Hulton et al., 2014[50]	Ethiopia, Ghana, Malawi, Nigeria, Sierra Leone, and Tanzania	Low and Middle	To outline and describe the Evidence for Action (E4A) program and its contributions to reduce rates of maternal and newborn mortality	Maternal and newborn mortality	Maternal Health/Wellbeing<br><br>Newborn health/wellbeing or stillbirths	Observational	Case studies of each of the E4A countries: Ethiopia, Ghana, Malawi, Nigeria, Sierra Leone, and Tanzania	Persuasion<br><br>Coercion	Communication/ Marketing<br><br>Guidelines<br><br>Regulation	Positive
Jeffery et al., 2004[51]	Macedonia	Middle	To outline the design and implementation of an education intervention aimed at improving perinatal health practices	Maternal and newborn mortality	Maternal Health/Wellbeing<br><br>Newborn health/wellbeing or stillbirths	Quasi-experimental	115 physicians and nurses	Education<br><br>Training		Positive
Jones et al., 2015[52]	Australia	High	To determine effectiveness of a complex intervention aimed at improving childcare nutrition and physical activity practices in childcare centres	Childhood nutrition and physical activity	Child Health/Wellbeing	Experimental	62 childcare centres participated in the intervention, while 60 centres served as the control	Education<br><br>Environmental Restructuring<br><br>Training<br><br>Enablement	Communication/ Marketing	Neutral
Kapungu et al., 2013[53]	Ghana	Middle	To reduce postpartum hemorrhage by designing and implementing a community-based misoprostol distribution intervention	Postpartum hemorrhage	Maternal Health/Wellbeing	Observational	74 healthcare providers	Education<br><br>Environmental Restructuring<br><br>Training<br><br>Enablement		Positive
Kingsnorth et al., 2020[54]	Canada	High	To address barriers and enablers to a knowledge translation intervention to improve clinic care	Children with physical disabilities	Child Health/Wellbeing	Observational	Healthcare providers	Education<br><br>Environmental Restructuring		Positive

			provided at a pediatric rehabilitation hospital	and complex health needs				Enablement<br><br>Persuasion		
Kraft et al., 2018 [55]	Ethiopia and Senegal	Low and Middle	To describe the perceived use and impact of using the WHO family planning guidance and tools	Family planning	Sexual or reproductive health/rights	Qualitative	Interviews with: 5 WHO regional advisers, 6 policy makers, 7 disseminators, 4 implementers and 4 end-users	Education	Guidelines	Positive
Lang et al., 2015[56]	USA	High	To improve healthcare providers' use of trauma-focused cognitive behavioural therapy and improve outcomes in children through the use of evidence-based practice	Mental health	Child Health/Wellbeing	Mixed methods	Across 16 participating agencies, 77 clinicians, 33 supervisors, and 23 senior leaders participated in training. 734 children across the agencies used the therapy approach	Education<br><br>Training<br><br>Persuasion		Positive
Makkar et al., 2016[57]	Australia	High	To test strategies to improve policymakers' use of Web Centre for Informing Policy in Health with Evidence from Research (CIPHER) usage and engagement with research evidence	Various sexual health topics (e.g., sexually transmissible infections, menopause, breast and cervical screening, and men's sexual health)	Sexual or reproductive health/rights	Observational	223 policymakers from 27 organisations accessed the platform during the study period	Education<br><br>Environmental Restructuring<br><br>Persuasion	Communication/ Marketing	Positive
Margolis et al., 2001[58]	USA	High	To improve the processes of health services and care delivery, particularly between healthcare providers and families, to support child health outcomes	Child health outcomes	Child Health/Wellbeing	Observational	8 pediatric and family healthcare practices participated	Education<br><br>Environmental Restructuring<br><br>Training<br><br>Enablement	Service Provision<br><br>Guidelines<br><br>Regulation	Positive
Mello et al., 2013[59]	USA	High	To identify adoption and implementation strategies for integrating an alcohol screening tool into regular practice for pediatric trauma patients	Alcohol use	Adolescent Health/Wellbeing	Mixed methods	7 pediatric trauma centres. A key informant was identified at each site<br><br>Qualitative: monthly check-in and feedback conversations with project managers at each site	Education<br><br>Training<br><br>Enablement	Service Provision	Positive

Melnyk et al., 2010[60]	USA	High	To implement the evidence-based Creating Opportunities for Personal Empowerment program and determine its effectiveness on nurses' beliefs of evidence-based practice	Premature birth	Newborn Health/Wellbeing or Stillbirths	Experimental	81 nurses (48 participated in implementation group and 33 participated in the control group)	Education<br><br>Training	Service Provision	Positive
Mody et al., 2013[61]	Bangladesh, China, Indonesia, Myanmar, Mongolia, Solomon Islands, Tonga, Vanuatu and Vietnam	Middle	To assess the use of family guidelines and associated tools as part of the Strategic Partnership Program	Family planning	Sexual or reproductive health/rights	Mixed methods	Workshop participants: program managers, Ministry of Health staff, clinicians and regional and country office staff from WHO/UNFPA.<br><br>Questionnaire: provided to Ministries of Health	Education<br><br>Training	Communication/ Marketing<br><br>Guidelines	Positive
Okonofua et al., 2011[62]	Nigeria	Middle	To describe the findings from multiple advocacy activities designed to provide free health services to pregnant women and children	Maternal and child mortality	Maternal Health/Wellbeing Child Health/Wellbeing	Observational	Interviews with 6 State Governors and/or Commissioners of Health	Education<br><br>Modelling<br><br>Persuasion	Communication/ Marketing<br><br>Guidelines	Positive
Ongolo-Zogo et al., 2018[63]	Cameroon and Uganda	Low and Middle	To assess how KT platforms have supported Millennium Development Goals through health system policy-making and decision making processes	Maternal and child health	Maternal Health/Wellbeing<br><br>Child Health/Wellbeing	Observational	Stakeholders and decision makers	Education<br><br>Environmental Restructuring<br><br>Training		Positive
Pariyo et al., 2005[64]	Uganda	Low	To determine the effects of scaling-up an intervention aimed at the integrated management of childhood illnesses to improve health outcomes in young children (under 5)	Childhood illness	Child Health/Wellbeing	Observational	316 healthcare facilities across 10 study districts. 427 healthcare workers participated across these facilities	Education<br><br>Training<br><br>Enablement		Neutral
Rowe et al., 2009[65]	Benin	Middle	To evaluate the effectiveness of additional training supports developed for healthcare providers who completed training in Integrated Management of Childhood Illness on child health outcomes	Childhood illness	Child Health/Wellbeing	Experimental	1244 consultations for any illness across 16 communes. Consultations were conducted by 267 healthcare workers at 114 health facilities.	Education<br><br>Environmental Restructuring<br><br>Training<br><br>Incentivisation		Neutral
Russell et al., 2010[66]	Canada	High	To evaluate knowledge brokers and their impact in a knowledge translation intervention and measurement tools to understand motor function in children with cerebral palsy	Cerebral palsy	Child Health/Wellbeing	Mixed methods	122 physiotherapists across three provinces	Education<br><br>Environmental Restructuring	Service Provision	Positive

Schreiber et al., 2009[67]	USA	High	The purpose of this study was to identify, implement, and evaluate strategies to support integrating scientific research evidence into clinical decision making	Physiotherapy	Child Health/Wellbeing	Observational	5 pediatric physiotherapists	Education<br>Environmental Restructuring<br>Training<br>Incentivisation		Positive
Schreiber et al., 2015[68]	USA	High	To outline a KT program designed to increase physiotherapists' knowledge and use of standardized outcome measures	Physiotherapy	Child Health/Wellbeing	Observational	17 physiotherapists from a pediatric outpatient facility	Education<br>Environmental Restructuring<br>Training<br>Modelling<br>Enablement<br>Persuasion		Positive
Schwartz et al., 2015[69]	USA	High	To use a multi-stakeholder partnership to develop and implement evidence-based breastfeeding support and training strategies	Breastfeeding	Maternal Health/Wellbeing	Quasi-experimental	8 health centres with predominantly culturally diverse patients implemented the 10 steps to support breastfeeding	Education<br>Environmental Restructuring<br>Training	Service Provision<br>Communication/ Marketing,<br>Guidelines<br>Environmental/ Social Planning	Positive
Segre et al., 2011[70]	USA	High	To describe the development and implementation of the train-the-trainer program aimed at maternal depression screening	Perinatal depression	Maternal Health/Wellbeing	Observational	42 individuals were trained from 32 maternal and child health agencies	Environmental Restructuring<br>Training		Positive
Segre et al., 2018[71]	USA	High	To describe a research and government partnership implementing 'listening visits' in health centres to support maternal mental health	Postpartum depression	Maternal Health/Wellbeing	Editorial	18 maternal health clinics	Education<br>Training<br>Enablement	Guidelines	Positive
Senarath et al., 2007[72]	Sri Lanka	Middle	To evaluate a program aimed at training healthcare providers to improve newborn care practices	Newborn care	Newborn Health/Wellbeing or Stillbirths	Experimental	120 healthcare providers. 59 participated in the intervention (27 midwives, 19 nurses and 13 physicians) while 61 participated in the control (26 midwives, 19 nurses and 16 physicians)	Education<br>Training		Positive

Seward et al., 2018[73]	Australia	High	To evaluate the effectiveness of a complex intervention aimed at improving compliance with child nutrition guidelines and children's dietary intake	Childhood nutrition	Child Health/Wellbeing	Experimental	45 childcare centres- 25 participated in the intervention and 20 participated in the control	Education<br><br>Environmental Restructuring<br><br>Persuasion	Guidelines	Positive
Simioni et al., 2017[74]	Argentina	Middle	To support the regionalization of a perinatal health strategy policy as a to improve health outcomes	Maternal and premature/ newborn health	Maternal Health/Wellbeing<br><br>Newborn Health/Wellbeing or Stillbirths	Mixed methods	Participants completed the first workshop and 49 participated in the second workshop	Training	Environmental/ Social Planning	Positive
Simmons, 2007[75] (Chapter 2)	Viet Nam	Middle	To describe the process of scaling up an intervention to provide injectable contraceptives to improve quality of care and family planning services	Contraception/ family planning	Sexual or reproductive health/rights	Observational	21 provinces in Viet Nam - Each provincial team included provincial health and family planning sector representatives	Education	Guidelines	Neutral
Simmons, 2007[76] (Chapter 8)	Brazil, Bolivia, Chile	Middle and High	To implement training the trainer methods to build capacity and provide innovative training in the area of reproductive health services	Reproductive health services	Sexual or reproductive health/rights	Mixed methods	Brazil: 9 training centre teams. 98 trainers were trained, and 1921 providers trained<br><br>Bolivia: 8 training centre teams. 34 trainers were trained, and 741 providers trained<br><br>Chile: 3 training centre teams. 21 trainers were trained, and 395 providers trained	Education<br><br>Environmental Restructuring<br><br>Training	Service Provision	Positive
Singh et al., 2013[77]	Ghana	Middle	To test local change ideas which could be scaled up later as part of a national child survival improvement project	Maternal and infant health	Maternal Health/Wellbeing<br><br>Newborn Health/Wellbeing or Stillbirths	Quasi-experimental	27 healthcare facilities	Education	Environmental/. Social Planning	Positive
Snelgrove-Clarke, 2010[78]	Canada	High	To evaluate two educational interventions to improve nurses' use of clinical guidelines during labour and delivery	Labour and delivery	Maternal Health/Wellbeing	Experimental	93 nurses participated in the first intervention and 62 nurses participated in the second intervention	Education<br><br>Enablement	Guidelines	Neutral

For peer review only

Sobel et al., 2011[79]	Philippines	Middle	To evaluate the impact of a training workshop for healthcare providers to provide newborns with a Hepatitis B vaccination, in line with a new vaccination policy	Newborn Hepatitis B vaccination	Newborn Health/Wellbeing or Stillbirths	Mixed methods	45 health centre staff, 120 community healthcare workers and 23 healthcare providers from 9 healthcare centers	Education<br><br>Environmental Restructuring	Guideline	Positive
Sundaram et al., 2015[80]	Ghana	Middle	To determine the effectiveness of a multi-stakeholder partnership program developed to provide comprehensive abortion and post-abortion care services	Abortion	Sexual or reproductive health/rights	Quasi-experimental	457 healthcare providers (116 physicians and 341 midwives, nurses, medical assistants) participated across 166 healthcare facilities.<br><br>The intervention group comprised 197 participants from 64 healthcare facilities. The first control group included 148 participants from 58 healthcare facilities, and the second control group included 112 participants across 44 healthcare facilities.	Environmental Restructuring<br><br>Training	Service Provision<br><br>Guidelines<br><br>Legislation	Positive
Tarasoff et al., 2014[81]	Canada	High	To evaluate the effectiveness of a theatre-based KT intervention design to improve healthcare providers' knowledge and attitudes of assisted human reproduction for LGBTQ people	Family planning	Sexual or reproductive health/rights	Qualitative	30 health professionals attended the workshop (e.g., physicians, nurses, social workers, health managers)	Training	Service Provision	Positive
Twum-Danso et al., 2014[82]	Ghana	Middle	To test the feasibility and scale up of a postnatal care policy to improve care	Postnatal care	Newborn Health/Wellbeing or Stillbirths	Quasi-experimental	30 teams from 27 rural health facilities	Education	Guidelines	Positive
Uneke et al., 2017[83]	Nigeria	Middle	To improve researchers and policymakers' the knowledge and capacity to use KT to promote evidence-informed policymaking in maternal, newborn and child health and wellbeing	Maternal, newborn and child health	Child Health/Wellbeing<br><br>Maternal Health/Wellbeing	Quasi-experimental	38 participants (e.g., researchers, government members) attended the workshop	Education		Positive

For peer review only

					Newborn Health/Wellbeing or Stillbirths					
Uneke et al., 2018[84]	Nigeria	Middle	To improve researchers and policymakers' competence to apply KT to promote evidence-informed policymaking in maternal, newborn and child health and wellbeing	Maternal, newborn and child health	Child Health/Wellbeing<br><br>Maternal Health/Wellbeing<br><br>Newborn Health/Wellbeing or Stillbirths	Quasi-experimental	45 participants (e.g., research team members, project managers or steering committee members, government health agency members, NGO and charity members)	Education<br><br>Environmental Restructuring		Positive
Uskun et al., 2008[85]	Turkey	Middle	To improve healthcare providers' knowledge of immunization and increase immunization coverage through an education intervention	Childhood vaccination	Child Health/Wellbeing	Quasi-experimental	229 primary healthcare workers (89 physicians,88 midwives, 38 health officers, 14 nurse)	Education		Positive
Vlad et al., 2016[86]	India	Middle	To describe a multi-stakeholder partnership to develop comprehensive clinical guidelines to reduce maternal mortality	Postpartum haemorrhage	Maternal Health/Wellbeing	Observational	400 healthcare staff working in 8 maternity wards	Environmental Restructuring<br><br>Training<br><br>Enablement	Environmental/ Social Planning	Positive
Warren et al., 2010[87]	Kenya	Middle	To evaluate the impact of a new postnatal intervention to improve healthcare workers' counselling on maternal and newborn health	Maternal and newborn health	Maternal Health/Wellbeing<br><br>Newborn Health/Wellbeing or Stillbirths	Quasi-experimental	76 healthcare providers from four health facilities	Education	Service Provision	Positive
Wolfe et al., 2014[88]	USA	High	To develop evidence-based guidelines and complementary training for healthcare providers to support sharing life-altering information in pediatric settings	Health communication	Child Health/Wellbeing	Quasi-experimental	142 healthcare providers (54 physicians, 32 fellows and residents, 18 medical students, 14 health assistants, 15 nurses, 6 physiotherapists, 3 social workers)	Education<br><br>Environmental Restructuring	Guidelines<br><br>Environmental/ Social planning	Positive

Wolfenden et al., 2017[89]	Australia	High	To evaluate the implementation and compliance with a healthy school canteen policy, nutrient quality of foods provided at the canteens	Childhood nutrition	Child Health/Wellbeing	Experimental	70 primary schools with canteens (35 assigned to both intervention and control groups)	Education<br><br>Environmental Restructuring<br><br>Training<br><br>Enablement<br><br>Persuasion<br><br>Incentivisation	Communication/ Marketing<br><br>Environmental/ Social Planning	Positive
Yoong et al., 2016[90]	Australia	High	This study aimed to examine the impact of providing printed educational materials on childcare service cooks' intentions to use nutritional guidelines and provide fruit and vegetables on their menu	Childhood nutrition	Child Health/Wellbeing	Experimental	77 childcare centres (38 acted as the control and 39 participated in the intervention)	Education	Communication/ Marketing	Positive

For peer review only

References

1. Abney-Roberts, S. E. A Successful Quality Improvement Project to Improve Infant Safe Sleep Practice. *J. Obstet. Gynecol. Neonatal Nurs.* **44**, S43 (2015).

2. Agapidaki, E. *et al.* A theory-based educational intervention to pediatricians in order to improve identification and referral of maternal depression: a quasi-experimental study. *Ann. Gen. Psychiatry* **12**, 37 (2013).

3. Agency for Healthcare Research and Quality. *AHRQ Safety Program for Perinatal Care: Experiences From the Frontline. Prepared under contract no. 2902010000241 (RTI International). AHRQ Publication No. 17-0003-23-EF. Rockville, MD: Agency for Healthcare Research and Quality. October 30, 2016. www.ahrq.gov/perinatalsafety.* (2016).

4. Ainsworth, R. M., Mog, C. & Summerlin-Long, S. A Comprehensive Newborn Falls Initiative One Year Later. *J. Obstet. Gynecol. Neonatal Nurs.* **43**, S66 (2014).

5. Akter, S. F. U., Heller, R. D., Smith, A. J. & Milly, A. F. Impact of a training intervention on use of antimicrobials in teaching hospitals. *J. Infect. Dev. Ctries.* **3**, 447–451 (2009).

6. Al-Rafay, S. S. & Al-Sharkawy, S. S. Educational outcomes associated with providing a comprehensive guidelines program about nursing care of preterm neonates receiving total parenteral nutrition. *Clin. Nurs. Res.* **21**, 142–158 (2012).

7. Alkon, A. *et al.* Nutrition and physical activity randomized control trial in child care centers improves knowledge, policies, and children's body mass index. *BMC Public Health* **14**, 215 (2014).

8. Allen, C. W. & Jeffery, H. Implementation and evaluation of a neonatal educational program in rural Nepal. *J. Trop. Pediatr.* **52**, 218–222 (2006).

9. Allen, M. & Schafer, D. J. Nurses Improving the Health of Mothers and Infants by Dancing the 10 Steps to Successful Breastfeeding. *J. Obstet. Gynecol. Neonatal Nurs.* **44**, S52–S52 (2015).

10. Alton, M., Frush, K., Brandon, D. & Mericle, J. Development and implementation of a pediatric patient safety program. *Adv. Neonatal Care Off. J. Natl. Assoc. Neonatal Nurses* **6**, 104–111 (2006).

11. Alvarez, E. *et al.* Developing evidence briefs for policy: a qualitative case study comparing the process of using a guidance-contextualization workbook in Peru and Uganda. *Health Res. Policy Syst.* **17**, 89 (2019).

12. Anaby, D., Korner-Bitensky, N., Law, M. & Cormier, I. Focus on participation for children and youth with disabilities: Supporting therapy practice through a guided knowledge translation process. *Br. J. Occup. Ther.* **78**, 440–449 (2015).

13. Ansbro, É. M. *et al.* Introduction of Syphilis Point-of-Care Tests, from Pilot Study to National Programme Implementation in Zambia: A Qualitative Study of Healthcare Workers' Perspectives on Testing, Training and Quality Assurance. *PloS One* **10**, e0127728 (2015).

14. Baer, V. L. *et al.* Implementing a program to improve compliance with neonatal intensive care unit transfusion guidelines was accompanied by a reduction in transfusion rate: a pre-post analysis within a multihospital health care system. *Transfusion (Paris)* **51**, 264–269 (2011).

15. Barnard, J. G. *et al.* Facilitators and barriers to the use of standing orders for vaccination in obstetrics and gynecology settings. *Am. J. Obstet. Gynecol.* **216**, 69.e1-69.e7 (2017).

16. Becker-Haimes, E. M., Franklin, M., Bodie, J. & Beidas, R. S. Feasibility and Acceptability of a Toolkit to Facilitate Clinician Use of Exposure Therapy for Youth. *Evid.-Based Pract. Child Adolesc. Ment. Health* **2**, 165–178 (2017).

17. Berglund, A., Lefevre-Cholay, H., Bacci, A., Blyumina, A. & Lindmark, G. Successful implementation of evidence-based routines in Ukrainian maternities. *Acta Obstet. Gynecol. Scand.* **89**, 230–237 (2010).

For peer review only

18. Boyko, J. A., Kothari, A. & Wathen, C. N. Moving knowledge about family violence into public health policy and practice: a mixed method study of a deliberative dialogue. *Health Res. Policy Syst.* **14**, 31 (2016).

19. Braddick, L. *et al.* A mixed-methods study of barriers and facilitators to the implementation of postpartum hemorrhage guidelines in Uganda. *Int. J. Gynaecol. Obstet. Off. Organ Int. Fed. Gynaecol. Obstet.* **132**, 89–93 (2016).

20. Brennan, M. M. *et al.* Paediatric resuscitation for nurses working in Ghana: an educational intervention. *Int. Nurs. Rev.* **60**, 136–143 (2013).

21. Brown, J. J., Wacogne, I., Fleckney, S., Jones, L. & Bhrolchain, C. N. Achieving early surgery for undescended testes: quality improvement through a multifaceted approach to guideline implementation. *Child Care Health Dev.* **31**, 119–119 (2005).

22. Burgoine, K. *et al.* Staged implementation of a two-tiered hospital-based neonatal care package in a resource-limited setting in Eastern Uganda. *BMJ Glob. Health* **3**, (2018).

23. Cameron, D., Russell, D. J., Rivard, L., Darrah, J. & Palisano, R. Knowledge brokering in children's rehabilitation organizations: perspectives from administrators. *J. Contin. Educ. Health Prof.* **31**, 28–33 (2011).

24. Carlo, W. A. *et al.* Educational impact of the neonatal resuscitation program in low-risk delivery centers in a developing country. *J. Pediatr.* **154**, 504-508.e5 (2009).

25. Carmona J.M.; Howe E.; Zapata R.; Stevens D.; Murphy J.K. The teen health improvement program: A system-wide initiative to improve the quality of care for adolescents served by the country's largest public hospital system. in (2015).

26. Close, K., Karel, M. & White, M. A pilot program of knowledge translation and implementation for newborn resuscitation using US Peace Corps Volunteers in rural Madagascar. *Glob. Health* **12**, (2016).

27. Crone, M. R. *et al.* Sustainability of the Prevention of Passive Infant Smoking Within Well-Baby Clinics. *Health Educ. Behav.* **33**, 178–196 (2006).

28. Cunningham, B. J. & Cardy, J. O. Using implementation science to engage stakeholders and improve outcome measurement in a preschool speech-language service system. *Speech Lang. Hear.* **23**, 17–24 (2020).

29. Daniels, K., Lewin, S., & Practice Policy Group. Translating research into maternal health care policy: a qualitative case study of the use of evidence in policies for the treatment of eclampsia and pre-eclampsia in South Africa. *Health Res. Policy Syst.* **6**, 12 (2008).

30. Davies, B. *et al.* Fetal health surveillance: a community-wide approach versus a tailored intervention for the implementation of clinical practice guidelines. *CMAJ Can. Med. Assoc. J. J. Assoc. Medicale Can.* **167**, 469–474 (2002).

31. Deorari, A. K., Paul, V. K., Singh, M., Vidyasagar, D., & Medical Colleges Network. Impact of education and training on neonatal resuscitation practices in 14 teaching hospitals in India. *Ann. Trop. Paediatr.* **21**, 29–33 (2001).

32. Di Noia, J., Schwinn, T. M., Dastur, Z. A. & Schinke, S. P. The relative efficacy of pamphlets, CD-ROM, and the Internet for disseminating adolescent drug abuse prevention programs: an exploratory study. *Prev. Med.* **37**, 646–653 (2003).

33. Ding, H. *et al.* Influencing the use of antibiotics in a Chinese pediatric intensive care unit. *Pharm. World Sci. PWS* **30**, 787–793 (2008).

34. Dobbins, M. *et al.* A randomized controlled trial evaluating the impact of knowledge translation and exchange strategies. *Implement. Sci.* **4**, 61 (2009).

35. Edwards, N. *et al.* The impact of leadership hubs on the uptake of evidence-informed nursing practices and workplace policies for HIV care: a quasi-experimental study in Jamaica, Kenya, Uganda and South Africa. *Implement. Sci. IS* **11**, 110 (2016).

36. English, K. C., Merzel, C. & Moon-Howard, J. Translating public health knowledge into practice: development of a lay health advisor perinatal tobacco cessation program. *J. Public Health Manag. Pract. JPHMP* **16**, E9–E19 (2010).

For peer review only

37. Eriksson, L., Bergström, A., Hoa, D. T. P., Nga, N. T. & Eldh, A. C. Sustainability of knowledge implementation in a low- and middle-income context: Experiences from a facilitation project in Vietnam targeting maternal and neonatal health. *PLOS ONE* **12**, e0182626 (2017).

38. Farner, R. *et al.* The nurse champion model for advancing newborn screening of critical congenital heart disease. *J. Obstet. Gynecol. Neonatal Nurs. JOGNN* **43**, 497–506 (2014).

39. Finch, M. *et al.* Impact of a population based intervention to increase the adoption of multiple physical activity practices in centre based childcare services: a quasi experimental, effectiveness study. *Int. J. Behav. Nutr. Phys. Act.* **9**, 101 (2012).

40. Finch, M. *et al.* Challenges of Increasing Childcare Center Compliance With Nutrition Guidelines: A Randomized Controlled Trial of an Intervention Providing Training, Written Menu Feedback, and Printed Resources. *Am. J. Health Promot. AJHP* **33**, 399–411 (2019).

41. George E.; Maloney F.; Kara N.; Bukoye B.; Benotti E.; Delaney M.M.; Nejad S.B.; Kumar N.D.; Semrau K. FACTORS DETERMINING THE UTILIZATION OF ANTENATAL CARE IN RURAL AND URBAN SETTINGS IN MOZAMBIQUE. *Int. J. Gynecol. Obstet.* **143**, 523 (2018).

42. Gera, R. *et al.* Implementation of "health systems approach" to improve vaccination at birth in institutional deliveries at public health facilities; experience from six states of India. *J. Fam. Med. Prim. Care* **8**, 1630–1636 (2019).

43. Gichane, M. W., Mutesa, M. & Chowa, G. Translating Evidence into Policy Change: Advocacy for Community-Based Distribution of Injectable Contraceptives in Zambia. *Glob. Soc. Welf.* **6**, 41–47 (2019).

44. Gilkey, M. B., Parks, M. J., Margolis, M. A., McRee, A.-L. & Terk, J. V. Implementing Evidence-Based Strategies to Improve HPV Vaccine Delivery. *Pediatrics* **144**, (2019).

45. Goering, M. & Wilson, W. Implementing preterm labor guidelines: a collaborative care improvement process. *J. Perinat. Neonatal Nurs.* **16**, 47–57 (2002).

46. Goyet, S. *et al.* Knowledge translation: a case study on pneumonia research and clinical guidelines in a low- income country. *Implement. Sci.* **9**, 82 (2014).

47. Grady, A. *et al.* Effectiveness of a dissemination strategy on the uptake of an online menu planning program: A controlled trial. *Health Promot. J. Aust. Off. J. Aust. Assoc. Health Promot. Prof.* **30 Suppl 1**, 20–25 (2019).

48. Guillory, C. *et al.* Texas Pulse Oximetry Project: A Multicenter Educational and Quality Improvement Project for Implementation of Critical Congenital Heart Disease Screening Using Pulse Oximetry. *Am. J. Perinatol.* **34**, 856–860 (2017).

49. Hardee, K. *et al.* Improving Voluntary, Rights-Based Family Planning: Experience From Nigeria And Uganda. *Open Access J. Contracept.* **10**, 55–67 (2019).

50. Hulton, L. *et al.* Using evidence to drive action: a 'revolution in accountability' to implement quality care for better maternal and newborn health in Africa. *Int. J. Gynaecol. Obstet. Off. Organ Int. Fed. Gynaecol. Obstet.* **127**, 96–101 (2014).

51. Jeffery, H. E. *et al.* The impact of evidence-based education on a perinatal capacity-building initiative in Macedonia. *Med. Educ.* **38**, 435–447 (2004).

52. Jones, C. O. H. *et al.* 'Even if you know everything you can forget': health worker perceptions of mobile phone text-messaging to improve malaria case-management in Kenya. *PloS One* **7**, e38636 (2012).

53. Kapungu, C. T. *et al.* A community-based continuum of care model for the prevention of postpartum hemorrhage in rural Ghana. *Int. J. Gynaecol. Obstet. Off. Organ Int. Fed. Gynaecol. Obstet.* **120**, 156–159 (2013).

54. Kingsnorth, S., Orava, T., Parker, K. & Milo-Manson, G. From knowledge translation theory to practice: developing an evidence to care hub in a pediatric rehabilitation setting. *Disabil. Rehabil.* **42**, 869–879 (2020).

For peer review only

55. Kraft, J. M. *et al.* Dissemination and use of WHO family planning guidance and tools: a qualitative assessment. *Health Res. Policy Syst.* **16**, 42 (2018).

56. Lang, J. M., Franks, R. P., Epstein, C., Stover, C. & Oliver, J. A. Statewide dissemination of an evidence-based practice using Breakthrough Series Collaboratives. *Child. Youth Serv. Rev.* **55**, 201–209 (2015).

57. Makkar, S. R., Howe, M., Williamson, A. & Gilham, F. Impact of tailored blogs and content on usage of Web CIPHER - an online platform to help policymakers better engage with evidence from research. *Health Res. Policy Syst.* **14**, 85 (2016).

58. Margolis, P. A. *et al.* From concept to application: the impact of a community-wide intervention to improve the delivery of preventive services to children. *Pediatrics* **108**, E42 (2001).

59. Mello, M. J. *et al.* Translation of alcohol screening and brief intervention guidelines to pediatric trauma centers. *J. Trauma Acute Care Surg.* **75**, S301-307 (2013).

60. Melnyk, B. M. *et al.* Translating the evidence-based NICU COPE program for parents of premature infants into clinical practice: impact on nurses' evidence-based practice and lessons learned. *J. Perinat. Neonatal Nurs.* **24**, 74–80 (2010).

61. Mody, S. K., Ba-Thike, K. & Gaffield, M. E. The World Health Organization-United Nations Population Fund Strategic Partnership Programme's implementation of family planning guidelines and tools in Asia-Pacific countries. *J. Obstet. Gynaecol. Res.* **39**, 825–830 (2013).

62. Okonofua, F., Lambo, E., Okeibunor, J. & Agholor, K. Advocacy for free maternal and child health care in Nigeria—Results and outcomes. *Health Policy* **99**, 131–138 (2011).

63. Ongolo-Zogo, P., Lavis, J. N., Tomson, G. & Sewankambo, N. K. Assessing the influence of knowledge translation platforms on health system policy processes to achieve the health millennium development goals in Cameroon and Uganda: a comparative case study. *Health Policy Plan.* **33**, 539–554 (2018).

64. Pariyo, G. W., Gouws, E., Bryce, J., Burnham, G., & Uganda IMCI Impact Study Team. Improving facility-based care for sick children in Uganda: training is not enough. *Health Policy Plan.* **20 Suppl 1**, i58–i68 (2005).

65. Rowe, A. K. *et al.* A multifaceted intervention to improve health worker adherence to integrated management of childhood illness guidelines in Benin. *Am. J. Public Health* **99**, 837–846 (2009).

66. Russell, D. J. *et al.* Using knowledge brokers to facilitate the uptake of pediatric measurement tools into clinical practice: a before-after intervention study. *Implement. Sci. IS* **5**, 92 (2010).

67. Schreiber, J., Stern, P., Marchetti, G. & Provident, I. Strategies to promote evidence-based practice in pediatric physical therapy: a formative evaluation pilot project. *Phys. Ther.* **89**, 918–933 (2009).

68. Schreiber, J., Marchetti, G. F., Racicot, B. & Kaminski, E. The use of a knowledge translation program to increase use of standardized outcome measures in an outpatient pediatric physical therapy clinic: administrative case report. *Phys. Ther.* **95**, 613–629 (2015).

69. Schwartz, R. *et al.* Washington 'Steps' Up: A 10-Step Quality Improvement Initiative to Optimize Breastfeeding Support in Community Health Centers. *J. Hum. Lact. Off. J. Int. Lact. Consult. Assoc.* **31**, 651–659 (2015).

70. Segre, L. S., Brock, R. L., O'Hara, M. W., Gorman, L. L. & Engeldinger, J. Disseminating perinatal depression screening as a public health initiative: a train-the-trainer approach. *Matern. Child Health J.* **15**, 814–821 (2011).

71. Segre, L. S., Trusty, S., Gullickson, R., Chuffo Davila, R. & O'Hara, M. W. Brokering the Evidence-Practice Gap: A Strategy for Moving Evidence Into Clinical Practice. *Psychiatr. Serv. Wash. DC* **69**, 852–854 (2018).

72. Senarath, U., Fernando, D. N. & Rodrigo, I. Effect of training for care providers on practice of essential newborn care in hospitals in Sri Lanka. *J. Obstet. Gynecol. Neonatal Nurs. JOGNN* **36**, 531–541 (2007).

For peer review only

73. Seward, K. *et al.* Improving the implementation of nutrition guidelines in childcare centres improves child dietary intake: findings of a randomised trial of an implementation intervention. *Public Health Nutr.* **21**, 607–617 (2018).

74. Simioni, A. T. *et al.* [Regionalization of perinatal health care in the province of Santa Fe, Argentina]. *Rev. Panam. Salud Publica Pan Am. J. Public Health* **41**, e38 (2017).

75. R Simmons, Fajans, P. & Ghiron, L. Chapter 2: Strategic choices in scaling up: introducing injectable contraception and improving quality of care in Viet Nam. in *Scaling up health service delivery: from pilot innovations to policies and programmes* (World health Organization, 2007).

76. R Simmons, Fajans, P. & Ghiron, L. Chapter 7 Scaling up family planning service innovations in Brazil: the influence of politics and decentralization. in *Scaling up health service delivery: from pilot innovations to policies and programmes* (World health Organization, 2007).

77. Singh, K. *et al.* Impact evaluation of a quality improvement intervention on maternal and child health outcomes in Northern Ghana: early assessment of a national scale-up project. *Int. J. Qual. Health Care* **25**, 477–487 (2013).

78. Snelgrove-Clarke, E. E. The effects of action learning on nurses' use of a fetal health surveillance guideline with low-risk labouring women. https://escholarship.mcgill.ca/concern/theses/dz010q44w.

79. Sobel, H. L. *et al.* Implementing a national policy for hepatitis B birth dose vaccination in Philippines: Lessons for improved delivery. *Vaccine* **29**, 941–945 (2011).

80. Sundaram, A., Juarez, F., Ahiadeke, C., Bankole, A. & Blades, N. The impact of Ghana's R3M programme on the provision of safe abortions and postabortion care. *Health Policy Plan.* **30**, 1017–1031 (2015).

81. Tarasoff, L. A., Epstein, R., Green, D. C., Anderson, S. & Ross, L. E. Using interactive theatre to help fertility providers better understand sexual and gender minority patients. *Med. Humanit.* **40**, 135–141 (2014).

82. Twum-Danso, N. A. *et al.* Using quality improvement methods to test and scale up a new national policy on early post-natal care in Ghana. *Health Policy Plan.* **29**, 622–632 (2014).

83. Uneke, C. J., Sombie, I., Uro-Chukwu, H. C., Johnson, E. & Okonofua, F. Using equitable impact sensitive tool (EQUIST) and knowledge translation to promote evidence to policy link in maternal and child health: report of first EQUIST training workshop in Nigeria. *Pan Afr. Med. J.* **28**, 37 (2017).

84. Uneke, C. J., Sombie, I., Uro-Chukwu, H. C. & Johnson, E. Using equitable impact sensitive tool (EQUIST) to promote implementation of evidence informed policymaking to improve maternal and child health outcomes: a focus on six West African Countries. *Glob. Health* **14**, 104 (2018).

85. Uskun, E., Uskun, S. B., Uysalgenc, M. & Yagiz, M. Effectiveness of a training intervention on immunization to increase knowledge of primary healthcare workers and vaccination coverage rates. *Public Health* **122**, 949–958 (2008).

86. Vlad, I. *et al.* Improving quality for maternal care - a case study from Kerala, India. *F1000Research* **5**, (2016).

87. Warren, C., Mwangi, A., Oweya, E., Kamunya, R. & Koskei, N. Safeguarding maternal and newborn health: improving the quality of postnatal care in Kenya. *Int. J. Qual. Health Care* **22**, 24–30 (2010).

88. Wolfe, A. D. *et al.* Sharing life-altering information: development of pediatric hospital guidelines and team training. *J. Palliat. Med.* **17**, 1011–1018 (2014).

89. Wolfenden, L. *et al.* Multi-strategic intervention to enhance implementation of healthy canteen policy: a randomised controlled trial. *Implement. Sci. IS* **12**, (2017).

For peer review only

90. Yoong, S. L. *et al.* A theory-based evaluation of a dissemination intervention to improve childcare cooks' intentions to implement nutritional guidelines on their menus. *Implement. Sci.* **11**, 105 (2016).

For peer review only

Preferred Reporting Items for Systematic reviews and Meta-Analyses extension for Scoping Reviews (PRISMA-ScR) Checklist

SECTION	ITEM	PRISMA-ScR CHECKLIST ITEM	REPORTED ON PAGE #
**TITLE**			
Title	1	Identify the report as a scoping review.	1
**ABSTRACT**			
Structured summary	2	Provide a structured summary that includes (as applicable): background, objectives, eligibility criteria, sources of evidence, charting methods, results, and conclusions that relate to the review questions and objectives.	3
**INTRODUCTION**			
Rationale	3	Describe the rationale for the review in the context of what is already known. Explain why the review questions/objectives lend themselves to a scoping review approach.	6
Objectives	4	Provide an explicit statement of the questions and objectives being addressed with reference to their key elements (e.g., population or participants, concepts, and context) or other relevant key elements used to conceptualize the review questions and/or objectives.	6
**METHODS**			
Protocol and registration	5	Indicate whether a review protocol exists; state if and where it can be accessed (e.g., a Web address); and if available, provide registration information, including the registration number.	6-7
Eligibility criteria	6	Specify characteristics of the sources of evidence used as eligibility criteria (e.g., years considered, language, and publication status), and provide a rationale.	7
Information sources*	7	Describe all information sources in the search (e.g., databases with dates of coverage and contact with authors to identify additional sources), as well as the date the most recent search was executed.	7-8
Search	8	Present the full electronic search strategy for at least 1 database, including any limits used, such that it could be repeated.	8
Selection of sources of evidence†	9	State the process for selecting sources of evidence (i.e., screening and eligibility) included in the scoping review.	8
Data charting process‡	10	Describe the methods of charting data from the included sources of evidence (e.g., calibrated forms or forms that have been tested by the team before their use, and whether data charting was done independently or in duplicate) and any processes for obtaining and confirming data from investigators.	9
Data items	11	List and define all variables for which data were sought and any assumptions and simplifications made.	9
Critical appraisal of individual	12	If done, provide a rationale for conducting a critical appraisal of included sources of evidence; describe	NA

St. Michael's
Inspired Care.
Inspiring Science.

For peer review only - http://bmjopen.bmj.com/site/about/guidelines.xhtml

SECTION	ITEM	PRISMA-ScR CHECKLIST ITEM	REPORTED ON PAGE #
sources of evidence§		the methods used and how this information was used in any data synthesis (if appropriate).	
Synthesis of results	13	Describe the methods of handling and summarizing the data that were charted.	9-10
**RESULTS**			
Selection of sources of evidence	14	Give numbers of sources of evidence screened, assessed for eligibility, and included in the review, with reasons for exclusions at each stage, ideally using a flow diagram.	10
Characteristics of sources of evidence	15	For each source of evidence, present characteristics for which data were charted and provide the citations.	10-13
Critical appraisal within sources of evidence	16	If done, present data on critical appraisal of included sources of evidence (see item 12).	NA
Results of individual sources of evidence	17	For each included source of evidence, present the relevant data that were charted that relate to the review questions and objectives.	Supplementary file
Synthesis of results	18	Summarize and/or present the charting results as they relate to the review questions and objectives.	10-18
**DISCUSSION**			
Summary of evidence	19	Summarize the main results (including an overview of concepts, themes, and types of evidence available), link to the review questions and objectives, and consider the relevance to key groups.	18-22
Limitations	20	Discuss the limitations of the scoping review process.	22-23
Conclusions	21	Provide a general interpretation of the results with respect to the review questions and objectives, as well as potential implications and/or next steps.	23-24
**FUNDING**			
Funding	22	Describe sources of funding for the included sources of evidence, as well as sources of funding for the scoping review. Describe the role of the funders of the scoping review.	25

JBI = Joanna Briggs Institute; PRISMA-ScR = Preferred Reporting Items for Systematic reviews and Meta-Analyses extension for Scoping Reviews.
* Where *sources of evidence* (see second footnote) are compiled from, such as bibliographic databases, social media platforms, and Web sites.
† A more inclusive/heterogeneous term used to account for the different types of evidence or data sources (e.g., quantitative and/or qualitative research, expert opinion, and policy documents) that may be eligible in a scoping review as opposed to only studies. This is not to be confused with *information sources* (see first footnote).
‡ The frameworks by Arksey and O'Malley (6) and Levac and colleagues (7) and the JBI guidance (4, 5) refer to the process of data extraction in a scoping review as data charting.
§ The process of systematically examining research evidence to assess its validity, results, and relevance before using it to inform a decision. This term is used for items 12 and 19 instead of "risk of bias" (which is more applicable to systematic reviews of interventions) to include and acknowledge the various sources of evidence that may be used in a scoping review (e.g., quantitative and/or qualitative research, expert opinion, and policy document).

From: Tricco AC, Lillie E, Zarin W, O'Brien KK, Colquhoun H, Levac D, et al. PRISMA Extension for Scoping Reviews (PRISMAScR): Checklist and Explanation. Ann Intern Med. 2018;169:467–473. doi: 10.7326/M18-0850.

For peer review only - http://bmjopen.bmj.com/site/about/guidelines.xhtml
